# Uncertainty in model‐based treatment decision support: Applied to aortic valve stenosis

**DOI:** 10.1002/cnm.3388

**Published:** 2020-08-05

**Authors:** Roel Meiburg, Wouter Huberts, Marcel C. M. Rutten, Frans N. van de Vosse

**Affiliations:** ^1^ Department of Biomedical Engineering Eindhoven University of Technology Eindhoven the Netherlands; ^2^ School for Cardiovascular Disease Maastricht University Maastricht the Netherlands

**Keywords:** aortic stenosis, Monte Carlo Markov chain, parameter estimation, patient specific, prediction uncertainty, unscented Kalman filter

## Abstract

Patient outcome in trans‐aortic valve implantation (TAVI) therapy partly relies on a patient's haemodynamic properties that cannot be determined from current diagnostic methods alone. In this study, we predict changes in haemodynamic parameters (as a part of patient outcome) after valve replacement treatment in aortic stenosis patients. A framework to incorporate uncertainty in patient‐specific model predictions for decision support is presented. A 0D lumped parameter model including the left ventricle, a stenotic valve and systemic circulatory system has been developed, based on models published earlier. The unscented Kalman filter (UKF) is used to optimize model input parameters to fit measured data pre‐intervention. After optimization, the valve treatment is simulated by significantly reducing valve resistance. Uncertain model parameters are then propagated using a polynomial chaos expansion approach. To test the proposed framework, three in silico test cases are developed with clinically feasible measurements. Quality and availability of simulated measured patient data are decreased in each case. The UKF approach is compared to a Monte Carlo Markov Chain (MCMC) approach, a well‐known approach in modelling predictions with uncertainty. Both methods show increased confidence intervals as measurement quality decreases. By considering three in silico test‐cases we were able to show that the proposed framework is able to incorporate optimization uncertainty in model predictions and is faster and the MCMC approach, although it is more sensitive to noise in flow measurements. To conclude, this work shows that the proposed framework is ready to be applied to real patient data.

## Graphical abstract

In this study, we aim to provide a framework to predict changes in haemodynamic parameters (as a part of patient outcome) after valve replacement treatment in aortic stenosis. The framework involves uncertain input parameter optimization using an unscented Kalman filter approach. Valve replacement treatment is then simulated, and uncertainties are propagated via polynomial chaos expansions. The framework is able to capture measurement quality in prediction confidence intervals and is ready to be applied to patient data.

**Novelty Statement**

- We developed a lumped parameter model to predict changes in haemodynamic parameters after valve replacement therapy in aortic stenosis
- We showed a data assimilation approach (Unscented Kalman Filter) is suitable for parameter optimization for this model based on available clinical data, with estimated optimization uncertainty
- We expanded parameter optimization via Unscented Kalman Filtering via sensitivity‐based weighting
- We coupled uncertain parameter optimization with a polynomial chaos expansion approach to propagate uncertain parameters in treatment prediction

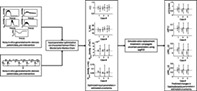

## 1 INTRODUCTION

In aortic stenosis (AS), calcifications and thickening of the leaflets of the aortic valve restrict leaflet motion, placing an additional haemodynamic load on the heart and reducing cardiac output. AS is mainly a disease of the elderly, with a prevalence of approximately 2.5% in the population ≥65 years of age.^1^ The most effective form of treatment for AS is valve replacement, where the surgical option is currently the gold standard.^2^ However, the transcatheter aortic valve implantation (TAVI) procedure provides a minimally invasive alternative that is gaining traction.^3^ During the TAVI procedure a stented valve is placed over a balloon on the end of a catheter and inserted via the groin. Once inside the stenotic valve, the balloon is inflated and the valve is placed. TAVI is considered when invasive surgery is not an option, for example, due to patient frailty.

Current treatment criteria are mainly based on the evaluation of the peak jet velocity and mean pressure gradient across the valve, determined via Doppler ultrasound and a simplified Bernoulli equation (2). While sufficient in many cases, these criteria have some drawbacks. First, these criteria assess stenosis severity but suffer from discordant grading in 30% of patients.^4^ Second, AS graded as severe via these criteria could also be asymptomatic.^5^, ^6^ Third, the rise of TAVI procedures has widened the range of patients eligible for valve replacement procedures, meaning that co‐morbidities will play a larger role in treatment planning. Finally, physical health after a clinically successful procedure is reported to remain unchanged or have decreased for approximately 20% of patients at 12‐month follow‐up.^7^ This leads us to assume that alternative diagnostic measures for TAVI treatment should be investigated.

Mathematical physics‐based methods have been applied to gain insight into the pathophysiology of the cardiovascular system since as early as the 1890s,^8^ and are maturing to the point where model‐based clinical decision support may be feasible.^9^ From an engineering perspective, identifying the effect of a restrictive valve replacement on certain haemodynamic parameters which characterize a patient (eg, cardiac work, stroke volume) seems the most intuitive predictor of a successful outcome and could possibly discriminate between a successful and unsuccessful treatment prior to intervention. This could be done by creating a patient‐specific circulation model which includes the heart with a stenotic valve and the systemic circulatory system. The total circulation model is formulated by selecting a model that is able to describe the underlying change in haemodynamics observed in literature, namely increased stroke volume, increased cardiac output,^10^ decreased left ventricular pressure and increased aortic pressure.^11^ Model input parameters are then adapted such that the model reflects the patient in the pre‐intervention state. Then, a virtual “surgery,” that is, reducing the valvular resistance to a healthy level, is performed and the model is used to calculate the post‐intervention relevant haemodynamic parameters. The difference can then be quantified, and used to inform the clinician on the impact of the intended treatment. For example, if the difference in these relevant haemodynamic parameters is deemed insufficient, a clinician could choose to refrain from intervening invasively and instead focus on medical treatment. However, model‐based, patient specific predictions still face a major challenge: how to handle accuracy in model predictions.

**FIGURE 1:**
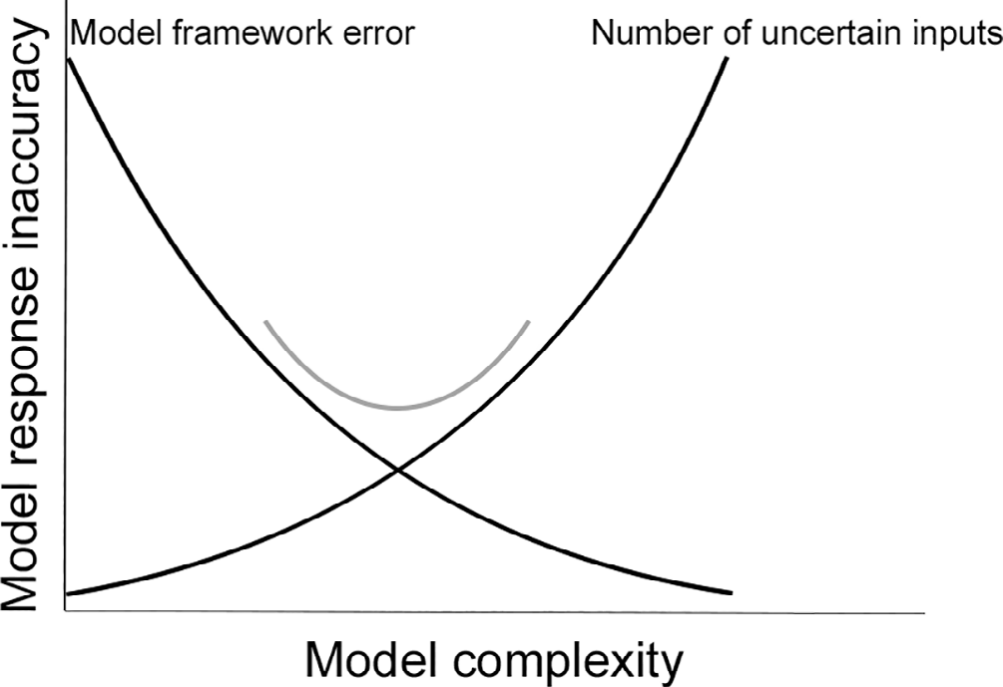
Model complexity vs model response accuracy. Simple models suffer from a high model framework error, whereas complex models suffer from a high epistemic uncertainty due to the large number of input parameters required, so there exists a trade‐off (adapted from Hanna^12^)

The response accuracy of a model prediction depends on model error and model uncertainty, represented graphically in Figure 1. Briefly, model error pertains to the known discrepancy between a model and the physical reality, for instance due to choosing a reduced form of governing equations or simplifying boundary conditions. Model error can be reduced by increasing the complexity of the model, at the cost of increasing the number of model input parameters. Model uncertainty describes the fact that any physical problem is not completely predictable due to uncertainties within the system we are trying to model. We distinguish between two types of model uncertainty, namely aleatory and epistemic uncertainty.^13^ Aleatory uncertainty is the natural randomness in the system we are trying to model. Epistemic uncertainty describes the uncertainty due to information which is theoretically knowable, but unavailable Oberkampf et al.^13^ provide the following examples:

“…*little or no experimental data for a fixed (but unknown) physical parameter, a range of possible values of a physical quantity provided by expert opinion, limited understanding of complex physical processes, and the existence of fault sequences or environmental conditions not identified for inclusion in the analysis of a system.”*

Aleatory and epistemic uncertainties are handled by quantifying input parameters not as scalar values, but as probability density functions or frequency distributions, and propagating them through the model to produce an uncertain outcome, via Monte Carlo,^14^ or more sophisticated methods such as stochastic collocation^15^, ^16^ or polynomial chaos expansion.^17^, ^18^, ^19^

A more complex model reduces the model error but the output uncertainty increases due to the increased number of model input parameters needed. In clinical practice, it is difficult (if not impossible) to assess all model input parameters for a patient, as the number and types of measurements are limited. Therefore, it is wise to prioritize those parameters which have the highest impact on the model output of interest. This is usually done using variance‐based sensitivity analysis, using Saltelli's Monte Carlo method^14^ or polynomial chaos expansions.^17^, ^18^, ^19^ The total set of model input parameters ***θ***_tot_ can then be divided into two subsets: the set of parameters to be optimized ***θ***_opt_ and the remaining parameters ***θ***_rem_. The parameters to be optimized have the highest impact on the model output of interest and its uncertainty should therefore be assessed patient‐specifically. The remaining unimportant parameters ***θ***_rem_ ⊂ ***θ***_tot_ can then be set to values within their uncertainty range, obtained from literature or heuristically.

However, not all model parameters can be measured directly. This requires us to solve an inverse problem or to utilize some numerical optimisation scheme. Generally, parameters are learned via model calibration, that is, minimizing some objective function which relates model outputs to measured data. This will result in a set of optimal parameters where the cost is minimal, while parameters near these optimal parameters will likely perform similarly in reproducing the measured data.^20^ Obtaining probability distributions of these optimized parameters requires more sophisticated methods, such as Bayesian estimation^21^, ^22^ or Monte Carlo‐Markov Chain (MCMC) methods.^23^, ^24^ The main drawback for these methods is the computational cost, as they require many evaluations of the forward problem. Recently, data assimilation (DA) techniques have been applied to the personalisation of cardiac models.^25^, ^26^ The advantage of DA techniques is that they provide a framework for explicitly accounting for all sources of uncertainty.^27^ DA techniques also offer extensions for identifiability analyses.^28^ In this work, we propose to use a data assimilation technique called the unscented Kalman filter (UKF) to quantify the uncertainty of estimated parameters that are important for the output of interest but cannot be measured. The UKF is a state estimator, which uses a system model and multiple sequential measurements to form an estimate of the system's varying quantities, or states.^29^ Due to its formulation, the UKF will produce an estimate of each input parameter for each time‐step, which can be recombined to generate probability densities for each parameter. The UKF requires significantly fewer model evaluations and therefore computation time than traditional optimization schemes. Furthermore, it can be used to perform a sensitivity analysis of the optimization, showing which input parameters are most sensitive to which measurements. This can inform clinicians on which measurement would need to be improved to reduce any prediction uncertainty.

We apply the UKF to valve replacement therapy for aortic stenosis. Together with two intervention cardiologists from the Catharina Hospital in Eindhoven, a list of 15 clinically relevant haemodynamic parameters was compiled (Table 1). These parameters pertain to the general cardiac performance of the patient (eg, cardiac output, ventricular volume/ejection fraction and aortic pressures), to the current criteria of a successful intervention (eg, pressure gradients) and new theoretical parameters (eg, stroke work^30^). In the event of a successful intervention, the load placed on the heart due to the restrictive valve is removed. The heart is then able to eject more blood during each beat, leading to a decreased systolic left ventricular volume and therefore increased cardiac output, stroke volume and ejection fraction. Due to the increased flow, pressures in the aorta should increase. There should be a significant reduction in pressure gradient across the aortic valve, and therefore a reduction in stroke work loss. Ventricular stroke work should decrease as the valve is no longer restrictive, while the cardiac work that remains is transferred better to the circulatory system.

**TABLE 1:** The 15 haemodynamic changes of interest, determined in cooperation with intervention cardiologists from the Catharina Hospital in Eindhoven

Haemodynamic parameter	Abbreviation	Units	Expected change
Cardiac output	*q*_CO_	L/min	Increase
Ventricular end diastolic volume	*V*_LV, diastole_	mL	Unchanged
Ventricular end systolic volume	*V*_LV, systole_	mL	Decrease
Stroke volume	*V*_stroke_	mL	Increase
Ejection fraction	EF	‐	Increase
Maximum ventricular pressure derivative	(*dp*_LV_/*dt*)_max_	mm Hg/s	Decrease
Systolic aortic pressure	*p*_Ao, systole_	mm Hg	Increase
Diastolic aortic pressure	*p*_Ao, diastole_	mm Hg	Increase
Mean aortic pressure	*p*_Ao, mean_	mm Hg	Increase
Maximum aortic valve pressure gradient	*dp*_AV, max_	mm Hg	Decrease
Mean aortic valve pressure gradient	*dp*_AV, mean_	mm Hg	Decrease
Ventricular stroke work	*W*_LV_	J	Decrease
Circulatory stroke work	*W*_Circ_	J	Increase
Stroke work loss	*W*_lost_	J	Decrease
Stroke work loss ratio	*W*_lost_/*W*_LV_	‐	Decrease

*Note*: These parameters were chosen as they describe the general cardiac performance of the patient, the current criteria of a successful intervention and new theoretical parameters. The change in these parameters is determined by significantly reducing the aortic valvular resistance and propagating the obtained uncertain input parameters.

To predict the change in haemodynamics post‐intervention we formulate a simple 0D model, adapted from several sources in literature. It consists of a left ventricular model where pressure and volume are related to myofibre stresses and strains,^31^ which has already been applied to modelling of a change in cardiac afterload.^32^ The afterload is a transmission line of three‐element Windkessel models, based on Laskey et al.^33^ The pre‐load is described as a fixed pulmonary venous pressure. Finally, the valve is modelled as a Bernoulli‐type resistance, similar to the model of Mynard et al^34^ without leaflet dynamics. This model is able to replicate to qualitatively replicate the haemodynamic changes that are observed directly post‐intervention, that is, increased stroke volume, increased cardiac output,^10^ decreased left ventricular pressure and increased aortic pressure.^11^

We will compare the probability distributions generated using the UKF to those generated using another suitable approach, namely the MCMC method, specifically the Metropolis‐Hastings algorithm (MH).^35^ The MH algorithm is extensively used in computer modelling applications due to its simplicity and generality. It utilizes a “random walk” to propose new input parameter value and uses the model to calculate the objective function, that is, the function to be minimized. If the proposed set of input parameters produces a lower objective function, the new parameters are accepted. Otherwise, the proposed set of input parameters is accepted/rejected using rejection sampling.^36^

We define three in silico test cases. All measurements are clinically feasible and are based on the work of Johnson et al.^37^ An overview of the cases is shown in Figure 5. The first case is the ideal case: it has pressure measurements in the left ventricle and aorta obtained via pressure wires, high quality left ventricular volume obtained from ultrasound images, and mitral and aortic flow readings using Doppler ultrasound. The case has twice the input parameter noise during the generation, and temporal resolutions of the pressure readings are reduced. Finally, the third case has the same input noise as the second case, but lacks direct left ventricular pressure measurements. Instead, ventricular pressure is estimated based on Doppler measurements and the Bernoulli equation. For each case, parameters are optimized with varying degrees of uncertainty, using both the UKF and MCMC method. The valve replacement is simulated in silico, and uncertain parameters obtained are propagated using adaptive sparse generalized polynomial chaos expansion (asgPCE). Finally, the change in 15 relevant haemodynamic parameters (Table 1) and their respective uncertainties are compared.

## 2 METHODS

### 2.1 Mathematical model

**FIGURE 2:**
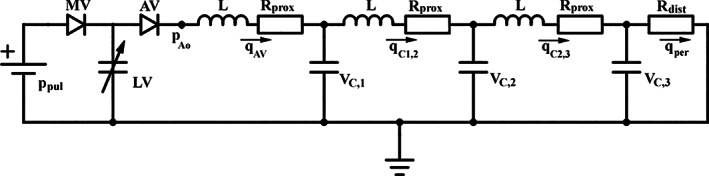
Lumped circulation model used in this study. The pre‐load is described as a fixed pulmonary venous pressure. The heart model relates ventricular pressure and volume via myofibre stress and strain.^31^ The valve model is based on Mynard et al,^34^ omitting valve leaflet dynamics. The systemic circulation is based on Laskey et al,^33^ as it has already been applied to aortic stenosis severity assessment

Figure 2 shows the lumped circulation model used in this study. In this model the left ventricle is modelled as a thick‐walled sphere, with muscle fibres running in the circumferential direction.^31^ By assuming that stretch and stress are homogeneous inside the ventricular wall, global properties (volume and pressure) can be related to myofibre stress and strain. Specifically, the pressure inside the ventricle is related to the stress experienced by the muscle fibres and the ratio of cavity and wall volume, that is,

(1) $$p_{\mathrm{LV}}=\frac{1}{3}\left(\sigma_{f}-2\sigma_{r}\right)\;\ln\;\left(1+\frac{V_{W}}{V_{\mathrm{LV}}}\right).$$

Here, *σ*_*f*_ and *σ*_*r*_ are the stresses in the fibre and radial direction, respectively, while *V*_W_ and *V*_LV_ represent the ventricular wall and cavity volume. Fibre stretch *λ*_*f*_ can be derived from the cavity volume *V*_LV_, unloaded cavity volume *V*_LV, 0_ and wall volume *V*_W_, as given by

(2) $$\lambda_{f}={\left(\frac{V_{\mathrm{LV}}+\frac{V_{W}}{3}}{V_{\mathrm{LV},0}+\frac{V_{W}}{3}}\right)}^{\frac{1}{3}}.$$

After the assumption of incompressibility, it holds that

(3) $$\lambda_{r}=\lambda_{f}^{-2}.$$

The stress experienced by the fibres are split into an active and passive component:

(4) $$\sigma_{f}=\sigma_{a,f}+\sigma_{p,f}.$$

A simple, non‐linear constitutive law is used to describe the passive stress‐stretch behaviour in the fibre and radial direction:

(5) $$\sigma_{i}=\left\{\begin{array}{ll} \sigma_{0,i}\;\left(\exp\left(c_{i}\left(\lambda_{i}-1\right)\right)-1\right) & \text{for}\;\lambda \geq 1\\ 0 & \text{for}\;\lambda <0 \end{array}\right..$$

Subscript *i* denotes the passive fibre or radial component, since material parameters *σ*_0, *i*_ and *c*_*i*_ differ for each component, due to anisotropy. The active component *σ*_*a*, *f*_ is dependent on contractility *c*, sarcomere length *l*_*s*_, time since activation *t*_*a*_ and sarcomere shortening velocity *v*_*s*_:

(6) $$\sigma_{a,f}=c\sigma_{\mathrm{ar}}f\left(l_{s}\right)g\left(t_{a}\right)h\left(v_{s}\right),$$

with

(7) $$f\left(l_{s}\right)=\left\{\begin{array}{ll} 0 & \text{for}\;l_{s}<l_{s,a0}\\ \frac{l_{s}-l_{s,a0}}{l_{s,\mathrm{ar}}-l_{s,a0}} & \text{for}\;l_{s}\geq l_{s,a0} \end{array}\right.,$$

(8) $$g\left(t_{a}\right)=\left\{\begin{array}{ll} 1-\frac{\cosh\left(t_{\text{sharp}}\left(\frac{2t_{a}}{t_{\max}}-1\right)-1\right)}{\cosh\left(t_{\text{sharp}}\right)-1} & \text{for}\;0\leq t_{a}\leq t_{\max}\\ 0 & \text{for}\;t_{\max}<t_{a}<1 \end{array}\right.,$$

with

(9) $$h\left(v_{s}\right)=1-\frac{v_{s}}{v_{0}}.$$

**FIGURE 3:**
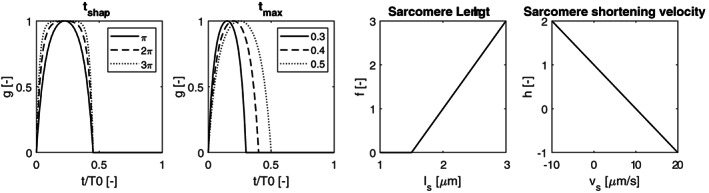
Active contraction functions. *t*_sharp_ governs the contractile shape, *t*_max_ describes the ratio between contraction duration and cycle time, both represented in function *g*. Finally the effect of sarcomere length *l*_*s*_ and sarcomere shortening velocity *v* are represented via functions *f* and *h*

Here, *l*_*s*, *a*0_ represents the sarcomere length in the unloaded state (*V*_LV_ = *V*_LV, 0_), while *l*_*s*, *ar*_ and *σ*_*ar*_ denote the fibre stress and sarcomere length in the reference state. Time *t*_max_ denotes the ratio between contraction duration and duration of a single heart cycle *T*_0_, while *t*_sharp_ governs the shape of the contraction curve. Note that the description of the activation curve differs from that of Bovendeerd et al,^31^ as this formulation allows for a better approximation of the physiological LV pressure signal shape as described in Guyton and Hall.^38^ Finally, *v*_*s*_ is the fibre shortening velocity in the unloaded state, while *c*_*v*_ describes the shape of the relationship between fibre stress and sarcomere shortening velocity. Figure 3 shows the general shapes of the contractile functions *f*, *h*, as well as the influence of *t*_sharp_, *t*_max_ on *g*.

The complex arterial network is reduced to a transmission line of 3 identical three‐element windkessel models, which describe vascular segments. They each consist of a viscous tube resistance (R), a capacitor (C), and an inductor (L). The pressure drop across each component is given by

(10) $$\Delta p_{R}=\mathrm{Rq},$$

(11) $$\Delta p_{L}=L\frac{\mathrm{dq}}{\mathrm{dt}},$$

(12) $$p_{C}=\frac{V}{C}.$$

The contribution of the pulmonary circulation is given as a constant pulmonary venous pressure *p*_pul_. Finally, the valves are considered as non‐linear Bernoulli resistors,^34^ where the pressure drop is given by

(13) $$\Delta P_{V}=\left\{\begin{array}{ll} B_{f}q|q| & \text{for}\;q\geq 0\\ B_{r}q|q| & \text{for}\;q<0 \end{array}\right..$$

with *B*_*f*_ and *B*_*r*_ the resistance for forward and regurgitant flow, respectively. Finally, this leads to the following total set of equations to be solved:

LV pressure:

(14) $$p_{\mathrm{LV}}=f_{\mathrm{sf}}\left(V_{\mathrm{LV}},\frac{{\mathrm{dV}}_{\mathrm{LV}}}{\mathrm{dt}},t,c,t_{\max},t_{\text{sharp}},V_{W}\right),$$

LV Volume change:

(15) $$\frac{{\mathrm{dV}}_{\mathrm{LV}}}{\mathrm{dt}}=q_{\mathrm{MV}}-q_{\mathrm{AV}},$$

Mitral valve flow:

(16) $$p_{\mathrm{pul}}-p_{\mathrm{LV}}=B_{\mathrm{MV}}q_{\mathrm{MV}}|q_{\mathrm{MV}}|,$$

Aortic valve flow:

(17) $$p_{\mathrm{LV}}-\frac{V_{C,1}}{C}=B_{\mathrm{AV}}q_{\mathrm{AV}}|q_{\mathrm{AV}}|+R_{\text{prox}}q_{\mathrm{AV}}+L_{\mathrm{art}}\frac{{\mathrm{dq}}_{\mathrm{AV}}}{\mathrm{dt}},$$

Aortic Pressure:

(18) $$p_{\mathrm{Ao}}=\frac{V_{C,1}}{C_{\mathrm{art}}}+R_{\text{prox}}q_{\mathrm{AV}},$$

Vessel segment 1 volume change:

(19) $$\frac{{\mathrm{dV}}_{C,1}}{\mathrm{dt}}=q_{\mathrm{AV}}-q_{C1,2},$$

Flow from vessel segment 1 to 2:

(20) $$\frac{V_{C1}-V_{C2}}{C}=R_{\text{prox}}q_{C1,2}+L_{\mathrm{art}}\frac{{\mathrm{dq}}_{C1,2}}{\mathrm{dt}},$$

Vessel segment 2 volume change:

(21) $$\frac{{\mathrm{dV}}_{C,2}}{\mathrm{dt}}=q_{C1,2}-q_{C2,3},$$

Flow from vessel segment 2 to 3:

(22) $$\frac{V_{C,2}-V_{C,3}}{C}=R_{\text{prox}}q_{C2,3}+L_{\mathrm{art}}\frac{{\mathrm{dq}}_{C2,3}}{\mathrm{dt}},$$

Vessel segment 3 volume change:

(23) $$\frac{{\mathrm{dV}}_{C,3}}{\mathrm{dt}}=q_{C2,3}-q_{\mathrm{per}},$$

and finally, flow to peripheral circulation:

(24) $$\frac{V_{C3}}{C}-0=R_{\text{dist}}q_{\mathrm{per}}.$$

Here, we refer to the total set of Equations (1) and (2) of the single fibre model as *f*_*sf*_ due to their cumbersome formulation. They are solved using the implicit Euler method, combined with Newton‐Raphson method with a fixed time‐step of 1 ms.

### 2.2 Parameter optimisation

Table 2 shows all model inputs, and how they were assessed. The first column contains all parameters that are estimated using the optimisation algorithm. In the second column all parameters that are assumed to be measurable or can be derived directly from the input signals themselves are listed. It should be noted that during the development phase we observed that non‐unique and non‐physiological combinations of *c*, *V*_0_, *V*_*W*_ were often obtained during the estimation process. However, it would be theoretically and clinically feasible to estimate the wall volume *V*_*W*_ directly from cardiac CT images with acceptable accuracy. Therefore, we assumed in further analyses that we also have a patient‐specific wall volume estimate available. The third column shows all parameter values which were based on literature and were set to population values. Similar to the Johnson et al^37^ cohort, valve regurgitation is not considered. Finally, we assume that the heart at myofibre level still functions as in a normal, healthy person. This assumption was also made by Pant et al^26^ and makes it possible to model cardiac function with the single‐fibre model of Bovendeerd et al,^31^ only making use of model parameters that can be assessed on a macroscopic level.

**TABLE 2:** All model parameters and their reference values of the proposed 0D model of this study, categorized by their derivation

Optimized	Value	Units	Measured	Value	Units	From Literature	Value	Units
*c*	1	[−]	*V*_*W*_	200	[*mL*]	*B*_*MV*, *r*_	10	[*mmHg* ⋅ *s*^2^/*mL*^2^]
*V*_0_	80	[*mL*]	*t*_*max*_	0.45	[−]	*B*_*AV*, *r*_	10	[*mmHg* ⋅ *s*^2^/*mL*^2^]
*t*_sharp_	3.14	[−]	*T*_0_	0.9	[*s*]	*σ*_*ar*_	410	[*mmHg*]
*B*_MV, f_	1 ⋅ 10^−2^	[*mmHg* ⋅ *s*^2^/*mL*^2^]				*l*_*s*, 0_	1.9	[*μm*]
*B*_AV, f_	5 ⋅ 10^−4^	[*mmHg* ⋅ *s*^2^/*mL*^2^]				*l*_*s*, *a*0_	1.5	[*μm*]
*L*_art_	1 ⋅ 10^−3^	[*mmHg* ⋅ *s*^2^/*mL*]				*l*_*s*, *ar*_	2.0	[*μm*]
*C*_art_	1	[*mL*/*mmHg*]				*v*_0_	10	[*μm*/*s*]
*R*_prox_	0.05	[*mmHg* ⋅ *s*/*mL*]				*c*_*v*_	0	[−]
*R*_dist_	1	[*mmHg* ⋅ *s*/*mL*]				*c*_*f*_	12	[−]
*p*_pul_	8	[*mmHg*]				*σ*_0, *f*_	6.75	[*mmHg*]
						*c*_*r*_	9	[−]
						*σ*_0, *r*_	1.5	[*mmHg*]

*Note*: We assume no valvular regurgitation, and no functional change of the cardiac tissue on a micro‐scale. Reference parameters for the left ventricle were kept at the values used by Cox et al,^39^ as were the parameters for the micro‐mechanical model. Windkessel parameters were set at the mean values reported by Laskey et al^33^ and pulmonary venous pressure was set to a physiological value.^38^ Finally, the reference value for the aortic valve resistance is approximately equal to an effective orifice area of 1 cm^2^_,_ ^34^ which is classified as severe AS.

#### 2.2.1 Unscented Kalman Filter

Parameter optimisation is performed using the Unscented Kalman Filter (UKF). The UKF is a state estimator, used in dynamic systems where observations suffer from white noise.^40^ The dynamic system is described by a model, which is the set of equations that describe its behaviour, that is,

(25) $$x_{k+1}=F\left(x_{k},\theta \right).$$

Here, *F* is the forward operator, which propagates the state vector **x**_*k*_ at time *t*_*k*_ forward to time *t*_*k* + 1_, resulting in states **x**_*k* + 1_. In this case, the states refer to the pressures *p*, volumes *V* and flows *Q* in the system:

(26) $$x_{k}={\left[p_{\mathrm{LV}},p_{\mathrm{Ao}},V_{C,1},V_{C,2},V_{C,3},V_{\mathrm{LV}},q_{\mathrm{MV}},q_{\mathrm{AV}},q_{C1,2},q_{C2,3},q_{\mathrm{per}}\right]}^{T}$$

and *F* is the time‐discretised form of the set of equations that make up the mathematical model, with ***θ*** the set of model input parameters which determine the behaviour of the model,

(27) $$\theta ={\left[c_{\mathrm{LV}},V_{\mathrm{LV},0},t_{\text{sharp}},B_{\mathrm{MV},f},B_{\mathrm{MV},r},B_{\mathrm{AV},f},B_{\mathrm{AV},r},L_{\mathrm{art}},C_{\mathrm{art}},R_{\text{prox}},R_{\text{dist}},p_{\mathrm{pul}}\right]}^{T}.$$

Note that, in this notation, ***θ*** only represents the set of input parameters which require optimization and omits input parameters that are measured directly or are kept at literature values (see Table 2).

In a Kalman filter, states are represented as a mean vector **x** and covariance matrix **P**, where **P** describes the uncertainty of each state in the diagonal, as well as the (non‐normalized) correlations between each of the states. The states of the model are coupled to the available measurements via a linear observation operator *H*,

(28) $$z_{k}=H\left(x_{k}\right)+\varepsilon .$$

where **z**_*k*_ is the set of measurements at time *t*_*k*_ and ***ε*** the (assumed) measurement noise.

**FIGURE 4:**
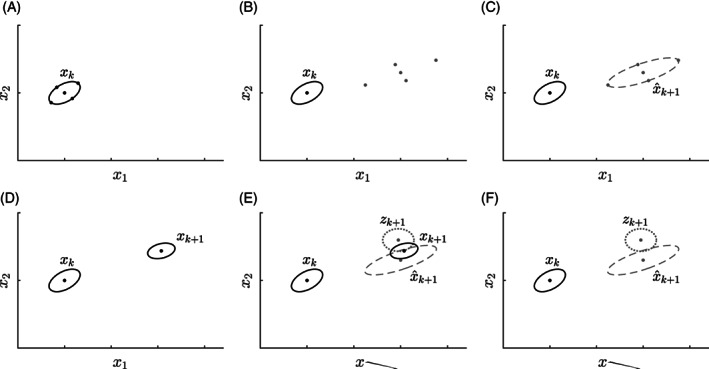
Visualization of the UKF for a two‐dimensional problem. The states are described as a mean vector **x** and a covariance matrix **P**, here represented as an ellipsoid. A, The mean vector is decomposed in to 2*L* + 1 sigma‐points. B, The decomposed sigma‐points are propagated to the next time‐step *t*_*k* + 1_ using the dynamic model. C, The propagated sigma‐points are recomposed to an a priori estimate of the propagated mean vector ${\hat{x}}_{k+1}$ and covariance matrix ${\hat{P}}_{k+1}$. D, The measurements of the states **z**_*k* + 1_ with noise estimate *ε* at time *t*_*k* + 1_ are introduced. E, Weighted by their relative uncertainties as described via **P**_*k* + 1_ and **R**, an a posteriori mean vector **x**_*k* + 1_ and covariance **P**_*k* + 1_ are produced. F, The final estimated **x**_*k* + 1_ and **P**_*k* + 1_

Kalman filters estimate the value and uncertainty of each state by repeating two steps, an a priori prediction and an a posteriori update. The prediction utilizes the dynamic model to propagate the estimated states **x**_*k*_ and covariance matrix **P**_*k*_ to the next time step *t*_*k* + 1_. Figure 4 shows a graphical representation for a 2D problem, consisting of the states *x*_1_ and *x*_2_.

In the UKF, the propagation is performed using a deterministic sampling technique known as the unscented transform.^41^ First, the mean estimate **x**_*k*_ and covariance matrix **P**_*k*_ are decomposed into a set of 2*L* + 1 representative points, where *L* is the number of states in **x**, also known as sigma‐points. These points are then individually propagated using the forward operator *F* [Equation (25)]. Finally, these points are then reconstructed into a newly estimated ${\hat{x}}_{k+1}$ and ${\hat{P}}_{k+1}$. Then, the state estimates are corrected using the observation vector **z**_*k* + 1_ weighted by their relative uncertainties, also known as the Kalman gain, leading to the final state estimate **x**_*k* + 1_ and **P**_*k* + 1_.

To perform the optimisation, the set of model parameters ***θ*** to be optimized is regarded as states by extending the state vector **x**_*k*_ and covariance matrix **P**_*k*_:

(29) $$x_{k,\mathrm{ext}}=\left[\begin{array}{c} x_{k}\\ \theta_{k} \end{array}\right]$$

(30) $$P_{k,\mathrm{ext}}=\left[\begin{array}{cc} \mathrm{Cov}\left(x_{k},x_{k}\right) & \mathrm{Cov}\left(x_{k},\theta_{k}\right)\\ \mathrm{Cov}\left(x_{k},\theta_{k}\right) & \mathrm{Cov}\left(\theta_{k},\mathrm{red}\theta_{k}\right) \end{array}\right].$$

The forward operator *F* is extended to incorporate the new state vector **x**_*k*, ext_, by adding trivial dynamics:

(31) $$\theta_{k+1}=\theta_{k},$$

meaning that the formulation of forward operator *F* transforms from Equation (25) to

(32) $${\left[x_{k+1},\theta_{k+1}\right]}^{T}=F\left({\left[x_{k},\theta_{k}\right]}^{T}\right).$$

Finally, to constrain the parameters to be estimated to ℝ^+^, for each parameter to be estimated, the following transformation is performed:

(33) $$\theta =\theta_{\mathrm{ref}}2^{\psi },$$

where *θ*_*ref*_ is a reference value for parameter *θ*, and *ψ* the parameter which is estimated via the UKF.^42^ Note that ***θ*** is now log‐normally distributed rather than normally distributed. Furthermore, this alters Equations (29), (30), (31)) to

(34) $$x_{k,\mathrm{ext}}=\left[\begin{array}{c} x_{k}\\ \psi_{k} \end{array}\right]$$

(35) $$P_{k,\mathrm{ext}}=\left[\begin{array}{cc} \mathrm{Cov}\left(x_{k},x_{k}\right) & \mathrm{Cov}\left(x_{k},\psi_{k}\right)\\ \mathrm{Cov}\left(x_{k},\psi_{k}\right) & \mathrm{Cov}\left(\psi_{k},\psi_{k}\right) \end{array}\right]$$

(36) $$\psi_{k+1}=\psi_{k}.$$

The UKF generates a parameter estimate in the form of a mean and variance for each timestep during the cardiac cycle. The probability density *P* of a parameter value *ψ*_*k*_ at time *t*_*k*_ is given by

(37) $$P\left(\psi_{k}|x_{k},\sigma_{k}\right)=\frac{1}{\sqrt{2{\pi \sigma }_{k}^{2}}}e^{-\frac{1}{2}{\left(\frac{\psi_{k}-x_{k}}{\sigma_{k}}\right)}^{2}},$$

where $\sigma_{k}^{2}$ is the variance in the corresponding diagonal element of the covariance matrix **P**_*k*_. We could obtain the probability density by recombining all probability densities, similar to kernel density estimation. However, model parameters do not vary when the observations *z* are insensitive to changes in those parameters. For instance, the UKF will not yield new estimates for contraction duration *t*_max_ during diastole, as changing it has no effect on the predicted observed states. Therefore, parameter estimates at each time‐step should be weighted accordingly. This is done by first computing the traditional non‐dimensionalized system sensitivity function:

(38) $$S_{i,k}=\left(\frac{1}{z_{i,\mathrm{ref}}}\right)\left(\frac{\partial h_{i,k}}{\partial \psi_{k}}\right).$$

Here *h*_*i*, *k*_ is the *i*‐th element of the observed model state vector *h*_*k* + 1_ = *H*(*x*_*k*_), where subscript *k* denotes time *t*_*k*_. The derivative $\frac{\partial h_{i,k}}{\partial \psi_{k}}$ is computed using a finite differences method. Reference value of the measurement *z*_*i*, ref_ is chosen to be max(*z*_*i*_). The weight *w* of the parameter value *ψ*_*k*_ at each time‐step *t*_*k*_ is then computed as the norm of the sensitivity function:

(39) $$w_{k}=\sqrt{\sum_{i}S_{i,k}^{2}}.$$

Then, the final probability distribution is taken as the weighted average of all probabilities across the cardiac cycle:

(40) $$P\left(\psi |x,\sigma \right)=\frac{\sum_{k}w_{k}P\left(\psi_{k}|x_{k},\sigma_{k}\right)}{\sum_{k}w_{k}}.$$

This approach also ensures that parameters which are not identifiable are diagnosed during the estimation procedure, as the sensitivity function will return zero.

### 2.3 Monte Carlo Markov Chain

In Bayesian optimization, we are interested in the posterior probability distribution *p*(*θ*| *z*), that is, the probability of model input parameters *θ*, given the measurements *z*. Since this probability distribution is hard to calculate directly, it is often sampled using a Monte Carlo Markov Chain approach, where the Metropolis Hastings^43^, ^44^ is the most widely and easily applied.^20^ Essentially, a Markov Chain is generated via a “random walk” through the parameter space. New steps are accepted/rejected based on the calculated probability. First, we define a sum of squares error function:

(41) $$\chi^{2}=\sum_{i=1}^{M}\sum_{k=1}^{N}\frac{{\left(z_{i}\left(t_{k}\right)-h_{i}(t_{k}|\theta )\right)}^{2}}{\sigma_{i}^{2}},$$

where we assume the residuals are mutually independent and Gaussian distributed. Here, *z*_*i*_(*t*_*k*_) represents the *i*‐th element of the measurement vector and *h*_*i*_(*t*_*k*_| *θ*) the *i*‐th element of the observed state vector, both at time *t*_*k*_. They are weighted by the measurement noise *σ*_*i*_. Assuming a non‐informative uniform prior, this leads to the following probability density^45^:

(42) $$p\left(\theta |y\right)\propto e^{-\chi^{2}/2}.$$

Obviously, the probability is maximal when the error function is minimal. To sample this distribution, we apply the “random walk” Metropolis Hastings method. It consists of three steps. First, we define an initial value *θ*_0_ and evaluate the likelihood *P*(*θ*_0_). Second, a candidate *θ*^′^ is proposed by adding a degree of Gaussian noise:

(43) $$\theta^{\prime }=\theta_{0}+\varepsilon .$$

Third, *P*(*θ*^′^| *z*) is evaluated, and the acceptance probability *A*(*θ*^′^, *θ*) is determined,

(44) $$A\left(\theta^{\prime },\theta_{0}\right)=\min\left(1,\frac{P\left(\theta^{\prime }\right)}{P\left(\theta \right)}\right).$$

If *θ*^′^ has a lower error *χ*^2^(*θ*^′^) and therefore higher likelihood *P*(*θ*^′^), *θ*^′^ is accepted as the new state, that is, *θ*_1_ = *θ*^′^. If *θ*^′^ has a higher error *χ*^2^(*θ*^′^)and therefore lower likelihood *P*(*θ*^′^), a uniform random number *u* ∈ [0, 1] is generated. If *u* ≤ *A*(*θ*^′^, *θ*_0_), the candidate is also accepted, that is, *θ*_1_ = *θ*^′^. Otherwise, the candidate is rejected and no jump is performed, that is, *θ*_1_ = *θ*_0_. This process repeats until the required amount of iterations is reached. Parameters will not immediately reach their equilibrium distribution, instead this must be inferred by examining parameter traces as well as the trace of *χ*^2^. For the MCMC algorithm to work optimally, the proposed jumps should be wide enough such that they avoid local minima and under mixing, but small enough that proposals are still regularly accepted. We therefore update the proposal perturbation *ε*_*k*_ via

(45) $$\varepsilon_{k}=\varepsilon_{k-1}\sqrt{\frac{1+n_{\mathrm{acc}}}{1+n_{\mathrm{rej}}}}.$$

Here *n*_acc_, *n*_rej_ are the number of accepted and rejected samples since the last update, respectively.^46^ All iterations before equilibrium are often discarded before parameter probability densities are calculated, also known as the *burn in* period. Finally, the parameter traces suffer from autocorrelation, since each set of accepted parameters is heavily correlated to the previous set of accepted parameters. Therefore, probability densities were calculated for every *n*‐th sample, where we varied *n* to be 1, 2, 5 and 10.

### 2.4 Case generation

**FIGURE 5:**
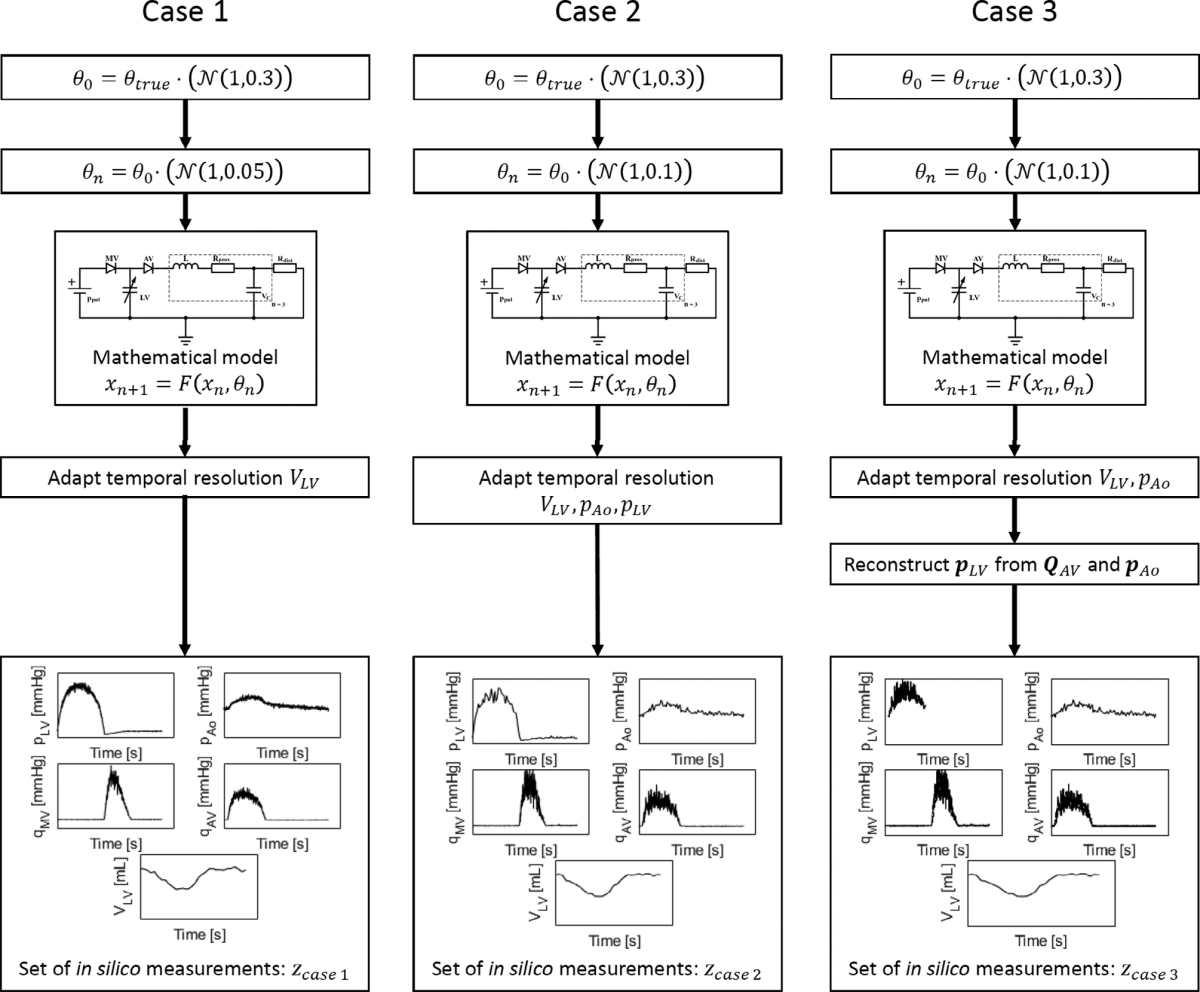
Case generation. We define the true values *θ*_0_. Then each case receives an initial estimate of *θ*_true_ named *θ*_0_, by adding a degree of Gaussian noise. All data are generated using the model as described in Section 2.1, with an additional Gaussian noise added for each time‐step. In Case 1, this parameter noise has a SD of 0.05 *θ*_0_, whereas Cases 2 and 3 this SD is 0.1 *θ*_0_. The only data available are those clinically feasible: ventricular and aortic pressures *p*_LV_, *p*_Ao_, mitral and aortic valve flow *q*_MV_, *q*_AV_ and ventricular volume *V*_LV_. Ventricular volume re‐sampled to 25 Hz, similar to the temporal frequency of ultrasound images. In Cases 2 and 3, the ventricular and aortic resolution is also reduced to 25 Hz. Finally, in Case 3 *p*_LV_ is not directly measured, but reconstructed using *p*_Ao_ and *q*_Ao_

To test the proposed optimization methods, three test‐cases are considered. Figure 5 shows the workflow for the generation of measurement data for the three different cases. Availability of data is based on Johnson et al^37^ and are therefore considered clinically feasible: ventricular and aortic pressures *p*_LV_, *p*_Ao_; ventricular volume *V*_LV_ and mitral and aortic valve flows *q*_MV_, *q*_AV_. Since the initial estimate *θ*_0_ should not be equal to the true estimate *θ*_true_, a certain degree of Gaussian noise is added:

(46) $$\theta_{0}=\theta_{\text{true}}\cdot N\left(1,0.3\right).$$

All data are generated using the model as described in Section 2.1, with an additional Gaussian noise added to input parameters at each time‐step. To be more in line with clinical ultrasound measurements, the temporal resolution of *V*_LV_ and therefore the observation function *H* are adapted from 1 to 40 ms. Case 1 represents a high quality case in the Johnson cohort. All measurement data are available and have with low measurement noise: ±5% for LV and aortic pressure, ±2.5% for LV volume and ±15% for aortic and mitral flow. Case 2 represents a less ideal patient, all data are available but with significantly higher noise: ±10% for LV and aortic pressure, ±2.5% for LV volume and ±25% for mitral and aortic flow. In Cases 2 and 3, the temporal resolution of the pressure measurements is also reduced to 40 ms. Case 3 represents a less‐than‐ideal patient from the cohort: data quality is similar to Case 2 and no ventricular pressure is available directly. Instead, ventricular pressure is reconstructed using *p*_Ao_ and *q*_AV_.

### 2.5 Treatment prediction

The treatment of interest for this work is the TAVI procedure, where a stented valve is placed on the end of a catheter and placed over the old valve. We assume that the immediate change in haemodynamics by placing a TAVI device can be described in the model by significantly reducing the forward resistance *B*_AV, f_. The probability densities obtained from the MCMC and the UKF methods will be propagated to the outputs of interest using asgPCE.^19^, ^47^ In short, asgPCE is a meta‐modelling technique, which utilizes a set of orthogonal polynomials to describe a direct relationship between model input and outputs. It requires significantly fewer model evaluations than traditional Monte Carlo simulations, and allows the user to evaluate the most important parameters after prediction.

### 2.6 Comparative analysis

**FIGURE 6:**
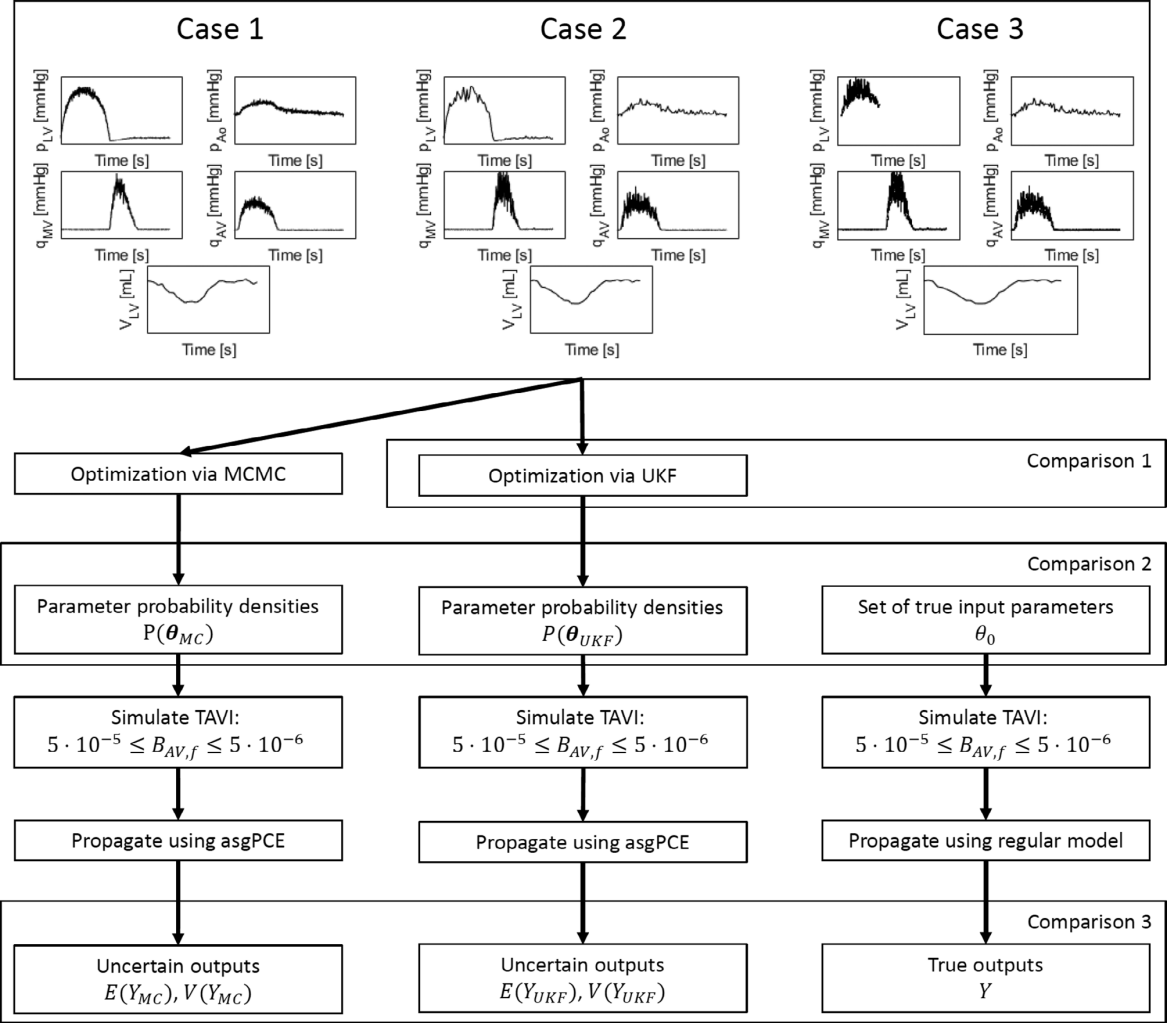
Comparative analyses of the performance of the more traditional MCMC method vs the proposed UKF approach. After optimization, the probability distributions of the input parameters are compared, as well as the “true” set of parameters. Finally, the effect of the different distributions are compared when they are propagated to the outputs of interest

To compare the results of the traditional MCMC to the proposed UKF, there are two moments of comparison, as shown in Figure 6. First, we compare the quality of Kalman optimization to the input data. Then, parameter distributions will be compared to one another, as well as the “true” set of input parameters. Since the relationship between parameter input distributions and model output distributions is not trivial, input distributions are propagated to outputs using the asgPCE method.^47^ Finally, the resulting output distributions are compared to investigate the actual predictive power of each method.

## 3 RESULTS

**FIGURE 7:**
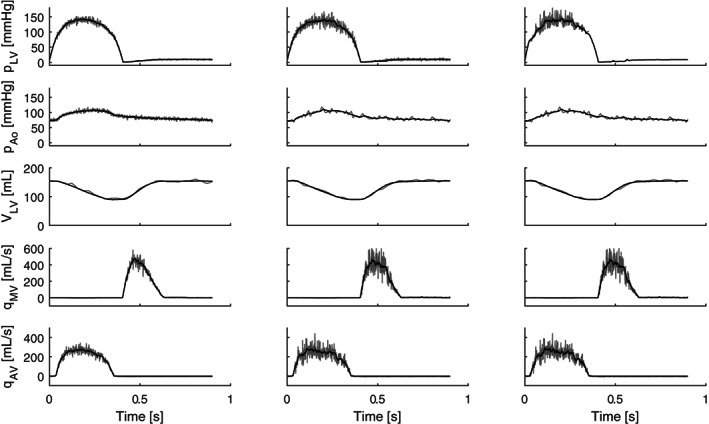
Observed states (grey) and their Kalman estimates (black) for all three cases. The quality of the observed states decreases in signal‐to‐noise as well as availability from Case 1‐3, as described in Section 2.4. The Kalman Filter is able to filter out most measurement noise, although the level of noise in estimated signal increases as measurement quality decreases

Figure 7 shows the observed states generated for each model case (grey), and their corresponding Kalman estimates (black). The quality of the observed states (noise, temporal resolution and availability) decreases from left to right. It also shows that, although the Kalman filter is able to filter out most measurement noise, the difference in estimation noise is already visible in the smoothness of the estimated states.

**FIGURE 8:**
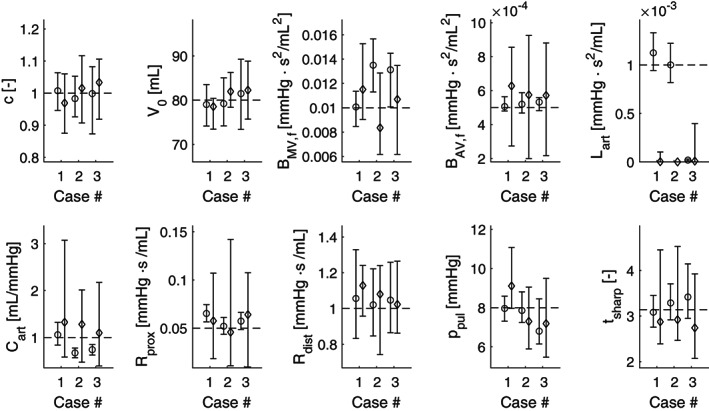
Estimated values of the model input parameters *θ*, described in Equation (27). Results are given as confidence intervals and optimal values after convergence, for the UKF (∘) and MCMC method (⋄), for all three cases

Figure 8 shows the optimal value as well as the 95% confidence intervals for each parameter, grouped per case, using the UKF method (∘) and the MCMC method (⋄). The dashed line represents the true input parameter values. This figure shows that the MCMC often returns wider CIs than the UKF approach, most notably in *B*_MV, f_, *B*_MV, f_, *C*_art_ and *R*_prox_. However, this is not the case for *V*_0_ and *R*_dist_ in Case 1. However, this also means that the UKF estimates differ from the true value more often, most notably in *B*_MV, f_ and *C*_art_. Finally, the MCMC approach is unable to identify *L*_art_, and parameter traces quickly reduce to 0, while this is only true for the UKF approach in Case 3.

**FIGURE 9:**
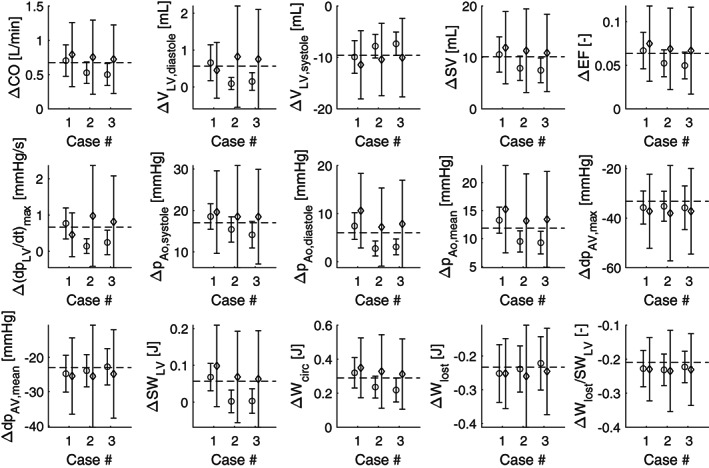
Estimated difference in interesting haemodynamic parameters as given in Table 1, after reducing the forward aortic valve resistance *B*_AV, f_ significantly to simulate a successful TAVI‐procedure and propagating the uncertain input parameters. Results are presented for all three cases, with input parameter confidence intervals determined via UKF (∘) and MCMC (⋄)

Treatment prediction is performed by significantly reducing the forward aortic valve resistance *B*_AV, f_ and propagating the obtained input uncertainty using asgPCE (Figure 6). Figure 9 shows the predicted treatment outcome in the form of a difference in haemodynamic parameters, for the UKF (∘) and the MCMC (⋄) with 95% confidence intervals. The dashed line represents the true difference in haemodynamics. Similar to Figure 8, the MCMC approach produces wider CIs than the UKF approach. However the UKF is therefore again more likely to significantly differ from the true output value, most notably in *V*_LV, diastole_, (*dp*_LV_/*dt*)_max_, *p*_Ao, diastole_ and *W*_LV_.

The sensitivities of the outputs of interest with respect to the input parameter uncertainty can be derived analytically during the uncertainty propagation in the form of (total) Sobol indices.^48^, ^49^ For the UKF approach, these Sobol indices show that the difference in *V*_LV, diastole_ and (*dp*_LV_/*dt*)_max_ are mostly determined by the overestimation of *B*_MV, f_, while the differences in the *p*_Ao, diastole_ and (*dp*_LV_/*dt*)_max_ can be traced back to the underestimation of *C*_art_. In turn, the overestimation of *B*_MV, f_ and underestimation of *C*_art_ are mainly due to the noise levels in mitral and aortic flow, respectively. For the MCMC approach, these Sobol indices show that the main contributors to predicted output uncertainty are mainly due to wide confidence intervals in *B*_MV, f_, *B*_AV, f_ and *C*_art_. The total Sobol indices for all model outputs and optimized inputs are provided in the Data S1.

## 4 DISCUSSION

In recent years, research in cardiovascular modelling is shifting towards patient‐specific (clinical) applications.^50^ Uncertainty quantification of model output is already becoming more prolific in cardiovascular research.^51^, ^52^, ^53^ However, uncertainty is often propagated from assumed input distributions, rather than based on optimisation uncertainty, which is in turn dependent on the quality (noise, temporal resolution, availability) to which the model is optimized. In this work, we have shown that the results of Kalman filter‐based optimisation can be translated into probability density estimates. These probability density estimates reflect the optimisation quality, which in turn can be used for model uncertainty propagation. This approach has several advantages to other approaches. First, as the UKF is a sequential estimator it utilizes each time‐step to steer the parameters towards their final value, significantly reducing computation time which is essential in a clinical setting. UKF‐based optimizations were performed within 90 s, while the MCMC approach took over 3 h, both on the same desktop PC. Secondly, the addition of the sensitivity functions can provide much needed information during the optimization process: which measured states contribute most to the optimization uncertainty.

Immediately, this requires us to address the derivation of the 95% confidence intervals based on the UKF optimization. The UKF provides an estimate for each parameter at each time‐step, ideally converging to a continually improving estimate. Following this logic, Pant et al^26^ chose the estimated values at the last time‐step as the set of optimized parameters. However, we found that parameter traces do not fully converge, but rather “oscillate” around a final value (see Data S1). In a follow‐up paper, Pant et al^54^ show a method to estimate parameters of a cardiovascular model with input data with varying heart rates, by extending the state vectors and propagation model in such a way that multiple cycles can be assimilated simultaneously. Then, the choice of when there is a “final” value becomes arbitrary, as the end of one cardiac cycle need not coincide with the rest. A more fair choice would then be to take into account multiple timesteps across the cardiac cycle. This leads to another issue, as for some parameters, no information is available during parts of the cardiac cycle. By weighting each parameter value according to their sensitivity to the observed states, this problem is mitigated and a fair estimate of each parameter is achieved.

The choice of model is vital for the quality of predictions, and should therefore be made carefully. In this work, we did not include autoregulation mechanisms, such as the baroreflex.^55^ While this will likely not impact the ability of the model to describe the patient in the stenotic state,^33^ it might affect the model predictions of the post‐intervention haemodynamics. However, incorporation of these autoregulation models means additional model complexity, which will significantly increase computation time in both the optimization and uncertainty propagation. Furthermore, the parameters which govern these autoregulation mechanisms are likely strongly correlated to the parameter values of the Windkessel models and likely significantly harder to identify. Additionally, these control mechanisms could be influenced during the valve replacement procedure by the anaesthetist, to stabilize the patient. Other long‐term physiological changes such as the decrease in pulmonary pressure after TAVI^56^ were also not taken into account, due to the lack of available data in these time‐scales.

Although the UKF approach can provide much information during the estimation process, problems such as identifiability and non‐uniqueness of solutions should ideally be examined beforehand. e.g., using sensitivity analyses^17^, ^57^ and identifiability analyses.^58^, ^59^ As minimizing computation time is essential for a clinical setting, we chose the agsPCE approach.^19^ However, it should be noted that this approach assumes statistically independent input parameters, which is (generally) not the case. While we have not investigated the effect of this assumption on the estimated output variance, the PCE approach could be extended to incorporate correlated input parameters.^60^, ^61^ In the formulation of this model, the risk of a non‐unique combination of cardiac parameters *c*_LV_, *V*_W_ and *V*_0_ leads to unlikely and unphysiological results during the optimisation, which is why *V*_W_ was assumed to be measured using CT imaging.

Kalman filter settings such as estimated model noise **Q** and filter noise ***ε*** and their results should always be critically evaluated. Ideally, the noise of a measurement relates directly to the SNR of the modality. However, this approach is not usable for each measurement type. For instance, cavity volumes of the left ventricle estimated via ultrasound images can vary due to assumptions on probe placement and inter−/intra‐operator variability. Quantifying measurement for these measurements can quickly become arbitrary. It is possible to choose **Q** and ***ε*** such that the model and Kalman estimate nearly completely overlap, but estimated parameters may either be unlikely or the probability distributions so wide that model predictions are futile. The reverse is also true, where the optimized parameters converge to a narrow distribution, but measurements and filter show little similarity. However, this should be eliminated if the chosen model suits the underlying mechanics, and the filter is set correctly.

Besides filter settings, this work shows that the initial guess *θ*_0_ and *P*_0_ should be chosen carefully.^42^, ^62^, ^63^ We have chosen an initial guess of $\theta_{0}=\theta_{\text{true}}\cdot N\left(1,\sigma \right)$, and varied 0 ≤ *σ* ≤ 0.5. We found that *σ* = 0.3 was the maximal value for which the UKF and MCMC consistently converged to the true value *θ*_true_. Values of *σ*_0_ > 0.3 often found other local minima or simply did not converge at all, even after more than 50 cardiac cycles.

A particular drawback is the need for time‐series data, which are not always available, especially in a clinical environment. In the case of aortic stenosis, not all of the required data are gathered during regular clinical workflow. All observations were based on the work of Johnson et al,^37^ indicating that they are at least clinically feasible. However, Case 3 shows that even without invasive pressure measurements, meaningful predictions could be made, albeit with a higher degree of uncertainty. Finally, it shows that more research is required to find a model formulation which is more easily optimized without the need for invasive pressure measurements.

## 5 CONCLUSION

Outcome of Trans‐Aortic Valve Implantation therapy in aortic stenosis cannot be determined solely on current diagnostic methods. Model‐based predicted change of important haemodynamic parameters post‐intervention could provide additional information for clinical decision support. However, to fully inform the clinician, uncertainty of model predictions should also be presented. This uncertainty is mostly due to availability and noise of clinical measurements, and should thus be incorporated in the predictive process. We present a framework which utilizes clinically feasible measurements and a lumped parameter model based on literature to predict 15 clinically important haemodynamic parameters in aortic stenosis patients. It employs the Unscented Kalman Filter (UKF) to optimize model parameters with an estimated uncertainty at a low computational cost. TAVI is simulated by significantly reducing valvular resistance, and uncertain input parameters are propagated using a polynomial chaos expansion to produce uncertain model predictions. By considering three in silico test‐cases we were able to show that the proposed framework is able to incorporate optimization uncertainty in model predictions and is faster than the MCMC approach, although it is more sensitive to noise in flow measurements. To conclude, this work shows that the proposed framework is ready to be applied to real patient data.

## CONFLICT OF INTEREST

The authors declare there is no conflict of interest.

## Supporting information


**Data S1.** Supporting information.
