# Supplementary figures and images for: Uncertainty in model‐based treatment decision support: Applied to aortic valve stenosis
Source: Int J Numer Method Biomed Eng. 2020 Aug 5;36(10):e3388. doi: 10.1002/cnm.3388 (PMC7583387; doi:10.1002/cnm.3388)

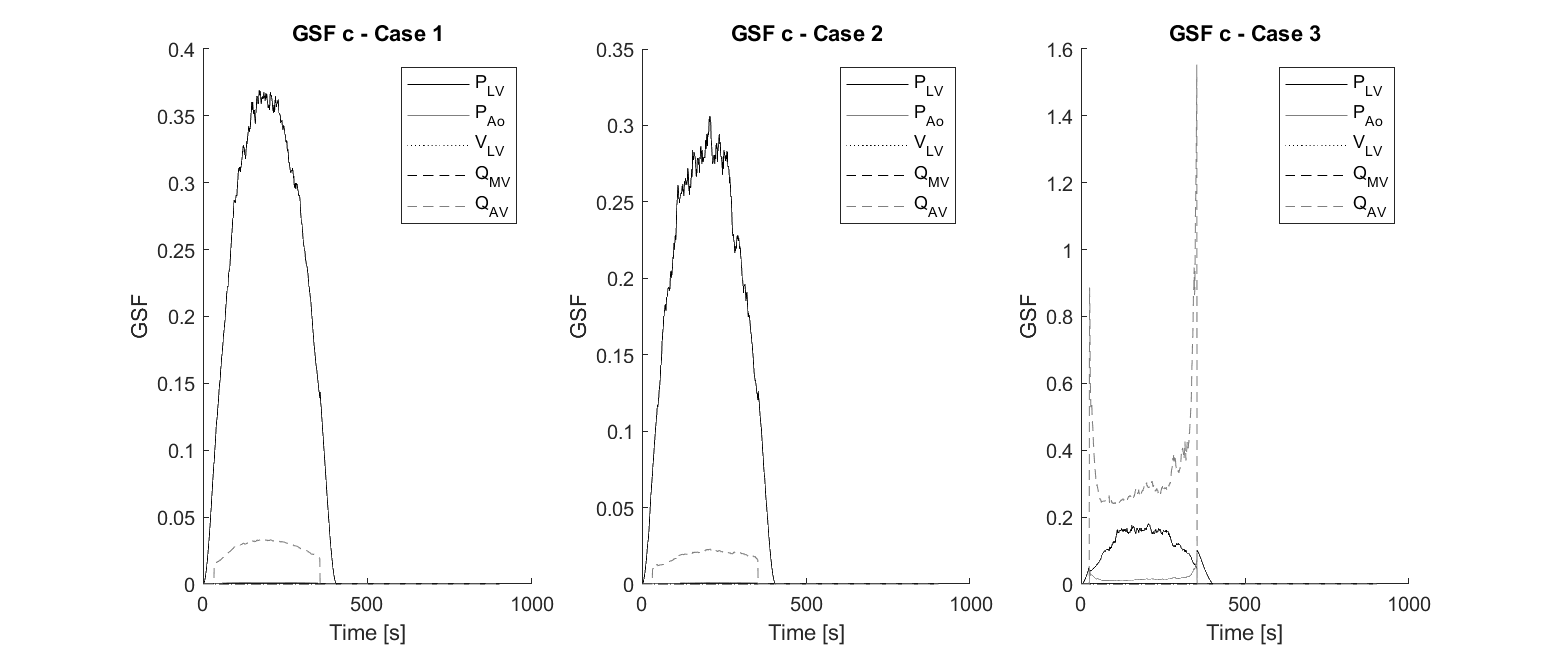

Supplement: Supplementary file 1 — Data S1. Supporting information. [file CNM-36-e3388-s001.zip › Supplementary Images/GSFParam1.png]

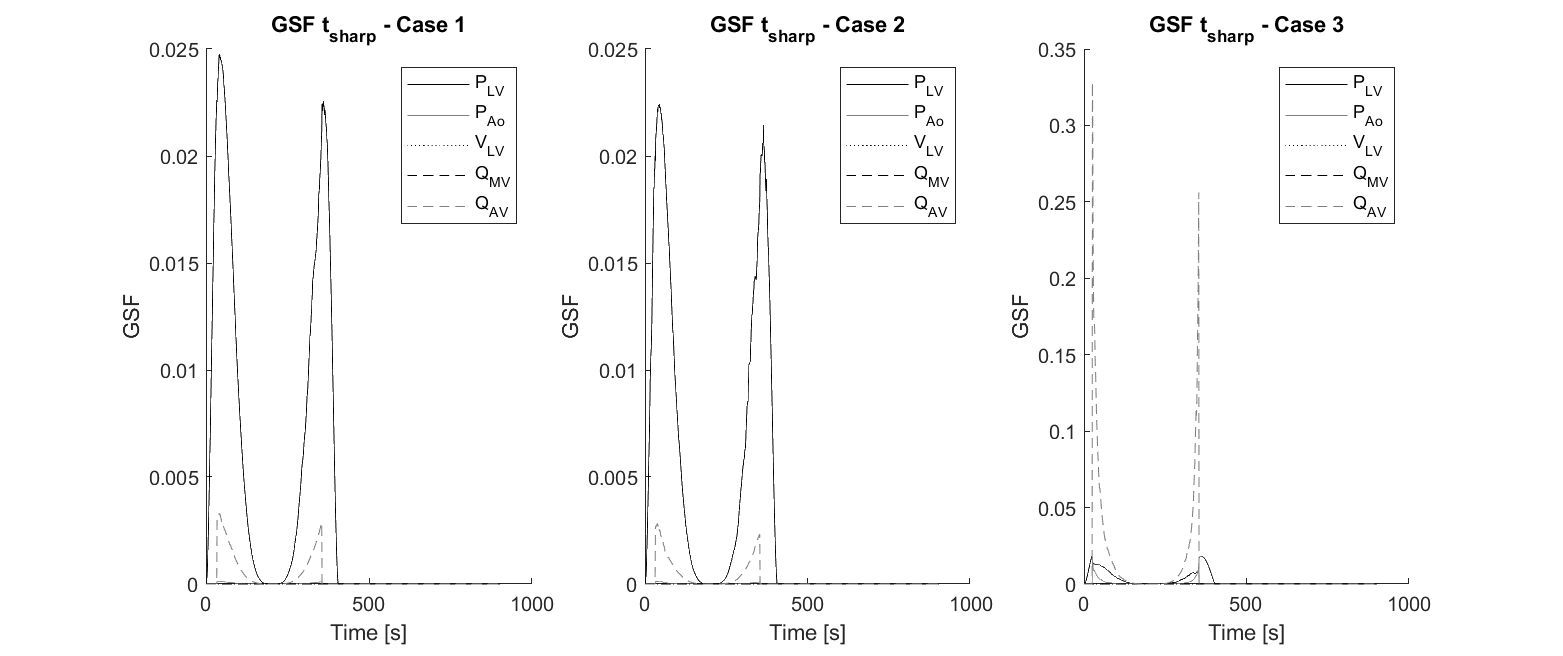

Supplement: Supplementary file 1 — Data S1. Supporting information. [file CNM-36-e3388-s001.zip › Supplementary Images/GSFParam10.png]

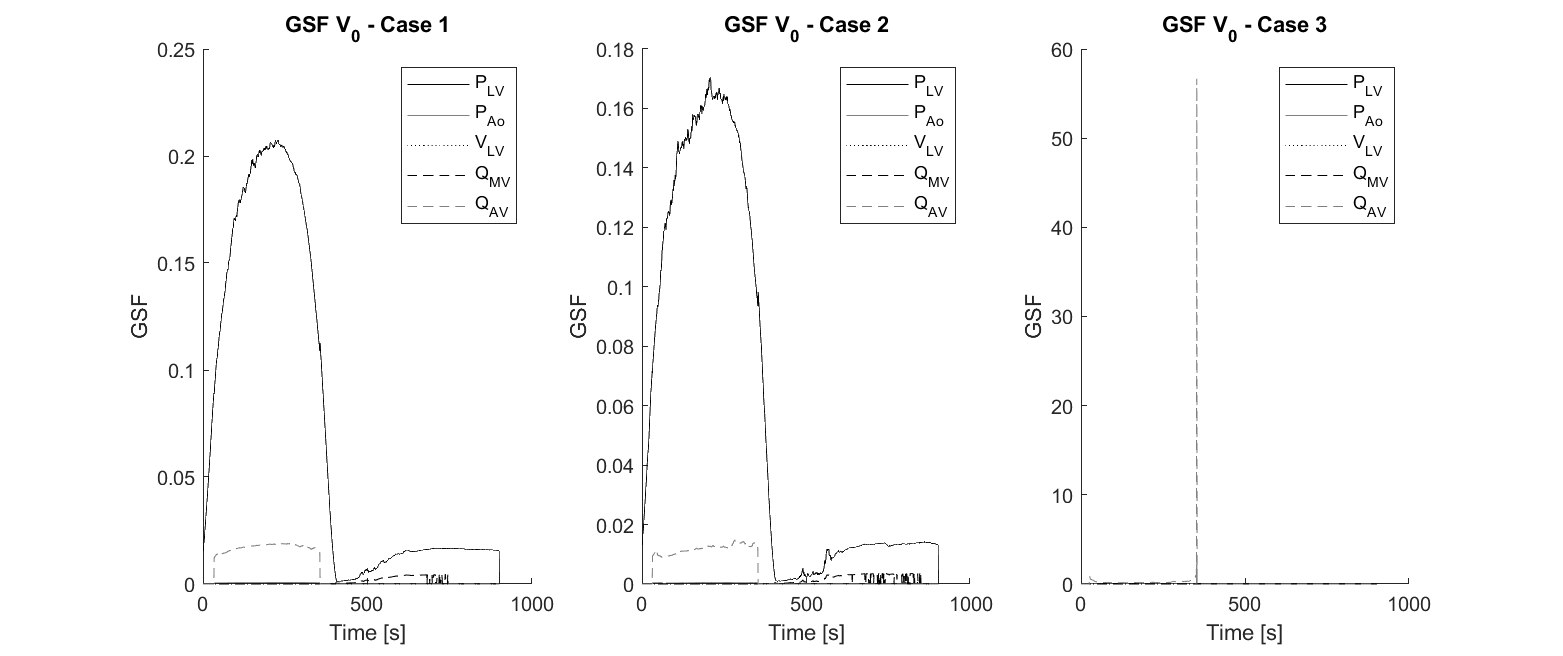

Supplement: Supplementary file 1 — Data S1. Supporting information. [file CNM-36-e3388-s001.zip › Supplementary Images/GSFParam2.png]

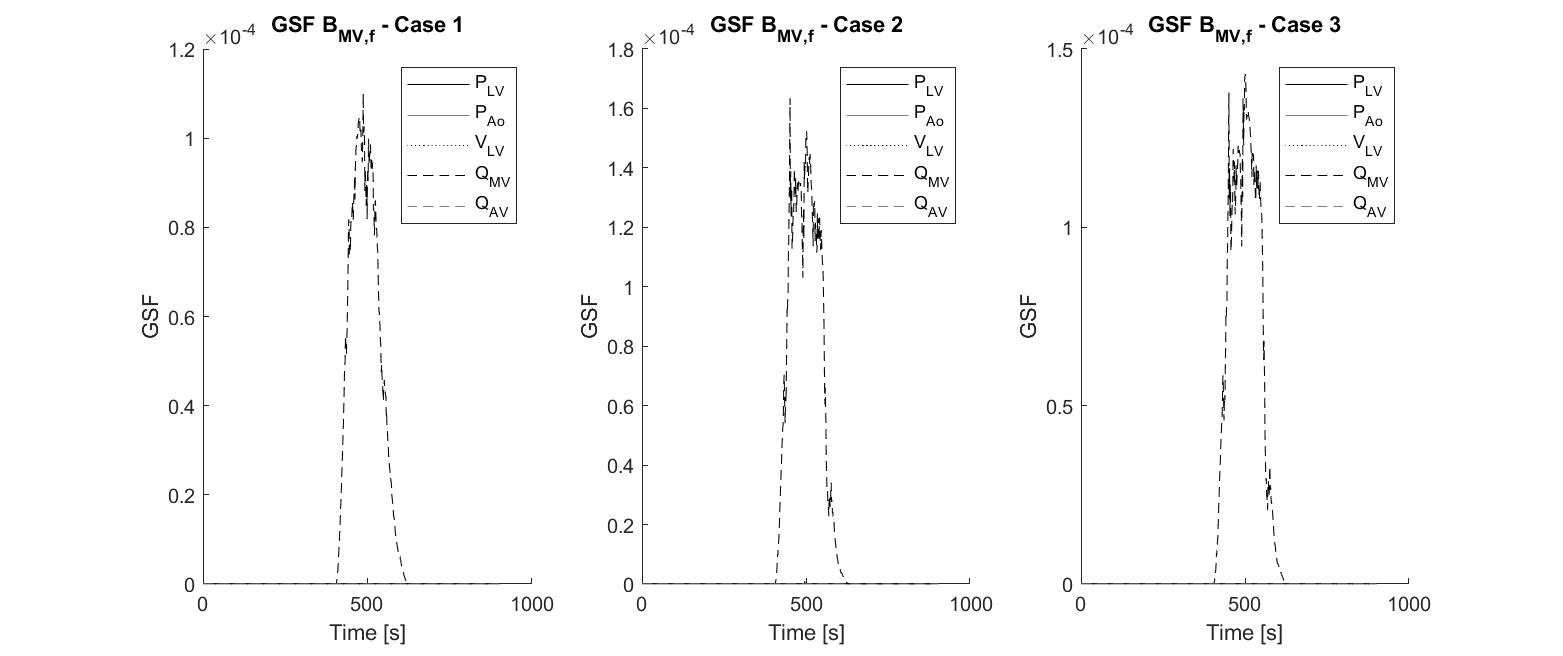

Supplement: Supplementary file 1 — Data S1. Supporting information. [file CNM-36-e3388-s001.zip › Supplementary Images/GSFParam3.png]

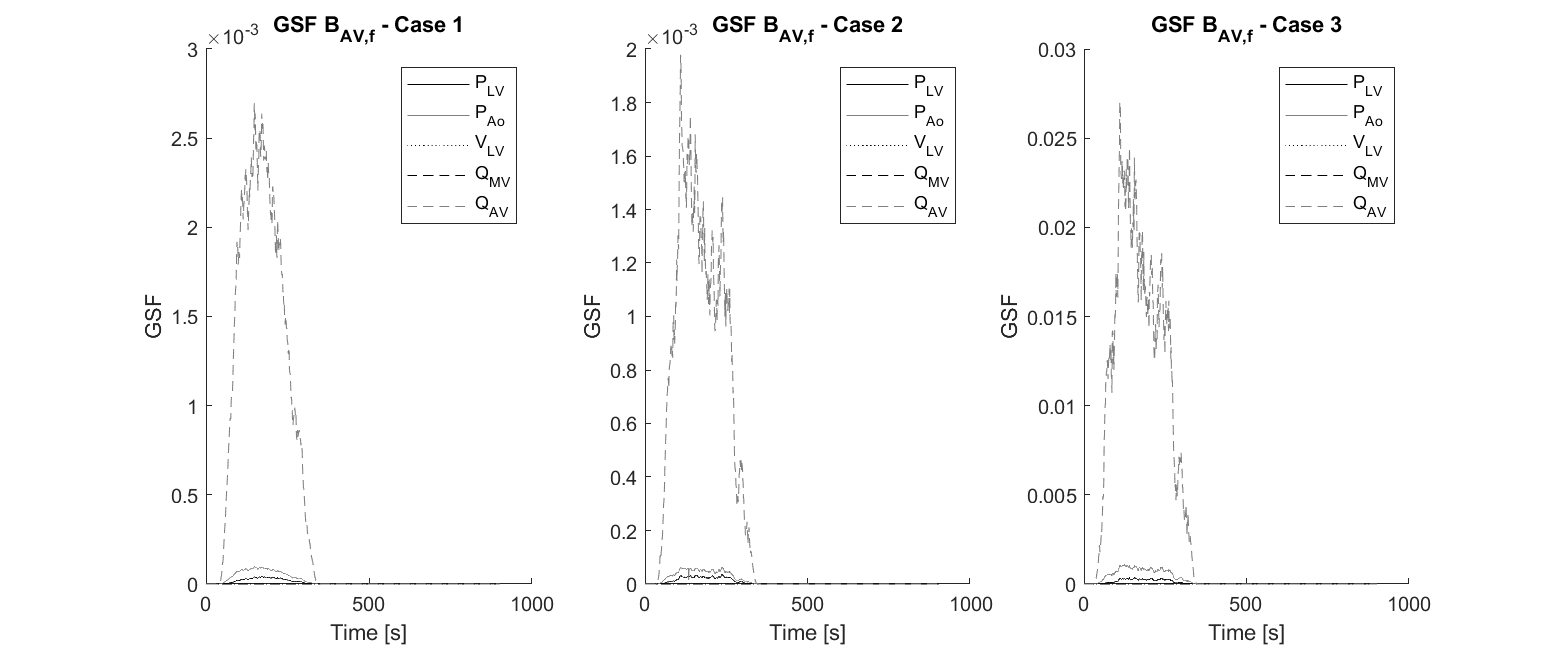

Supplement: Supplementary file 1 — Data S1. Supporting information. [file CNM-36-e3388-s001.zip › Supplementary Images/GSFParam4.png]

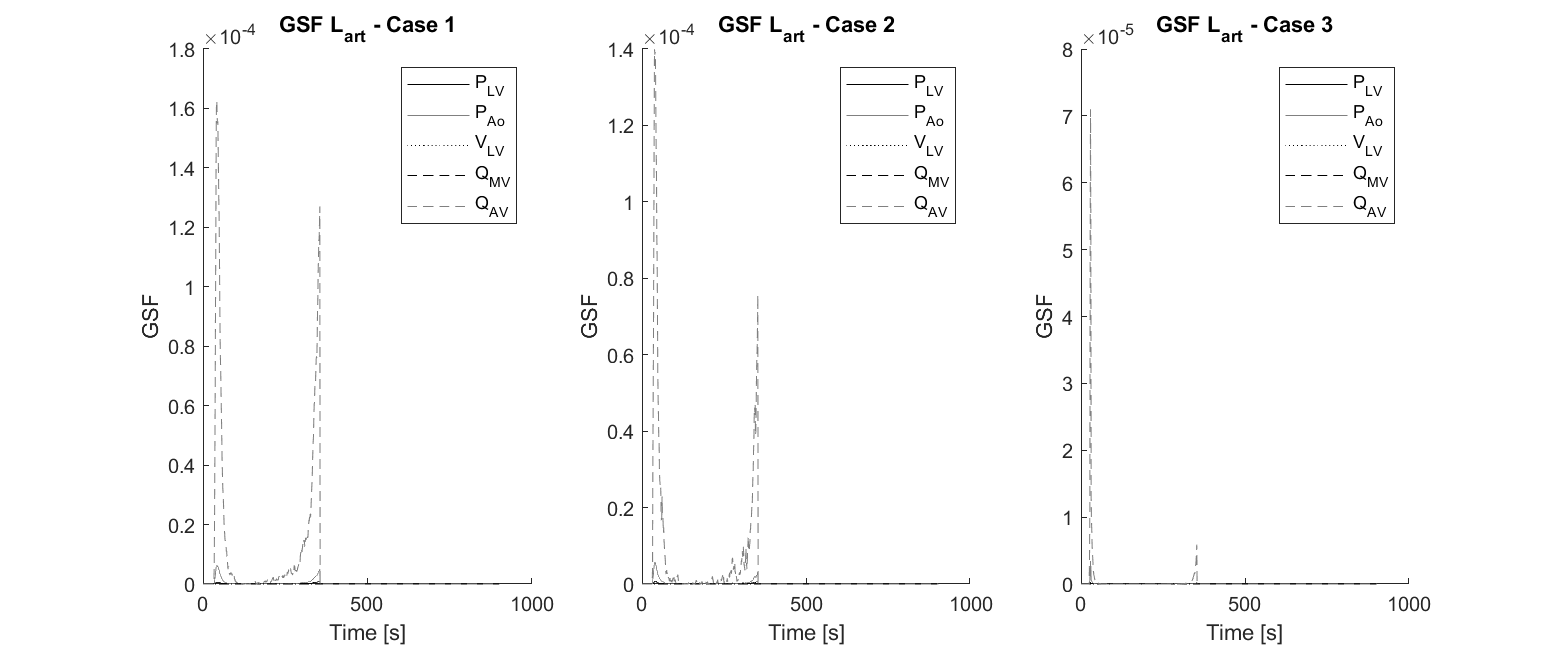

Supplement: Supplementary file 1 — Data S1. Supporting information. [file CNM-36-e3388-s001.zip › Supplementary Images/GSFParam5.png]

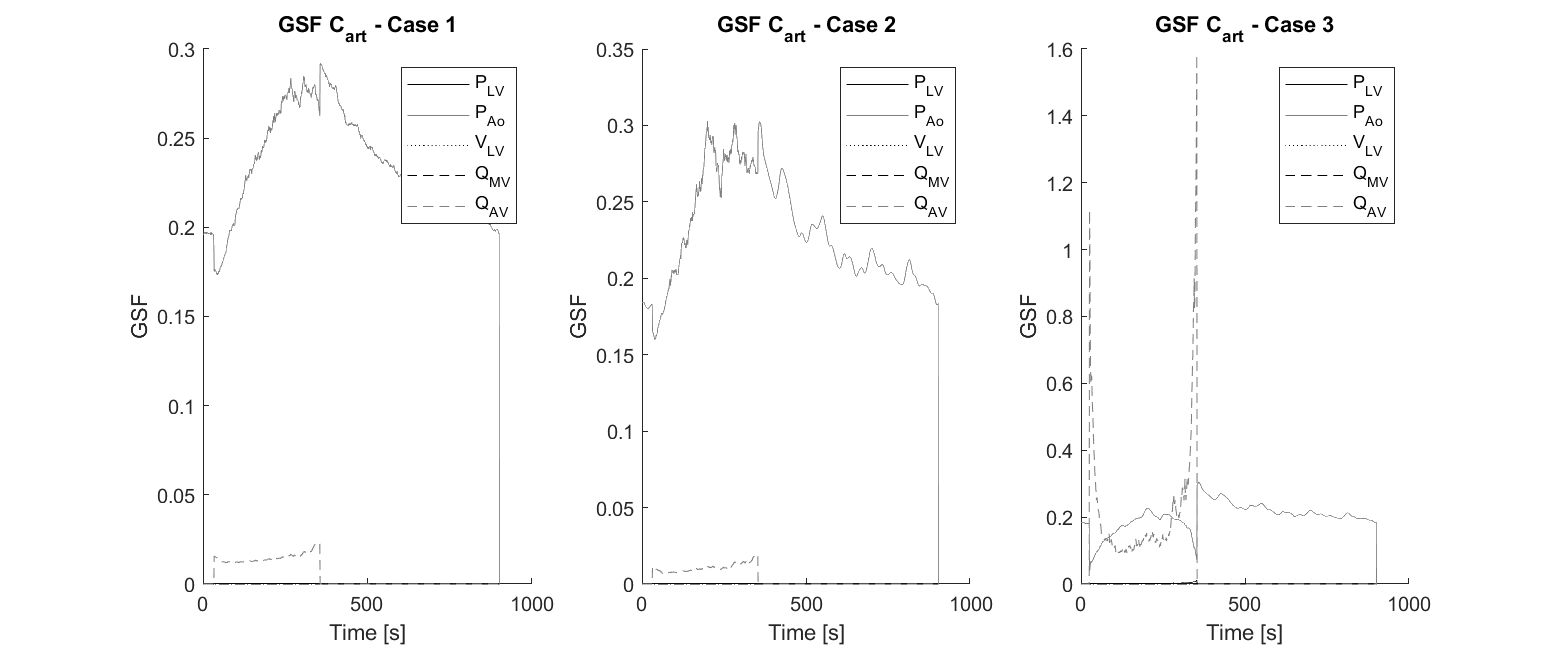

Supplement: Supplementary file 1 — Data S1. Supporting information. [file CNM-36-e3388-s001.zip › Supplementary Images/GSFParam6.png]

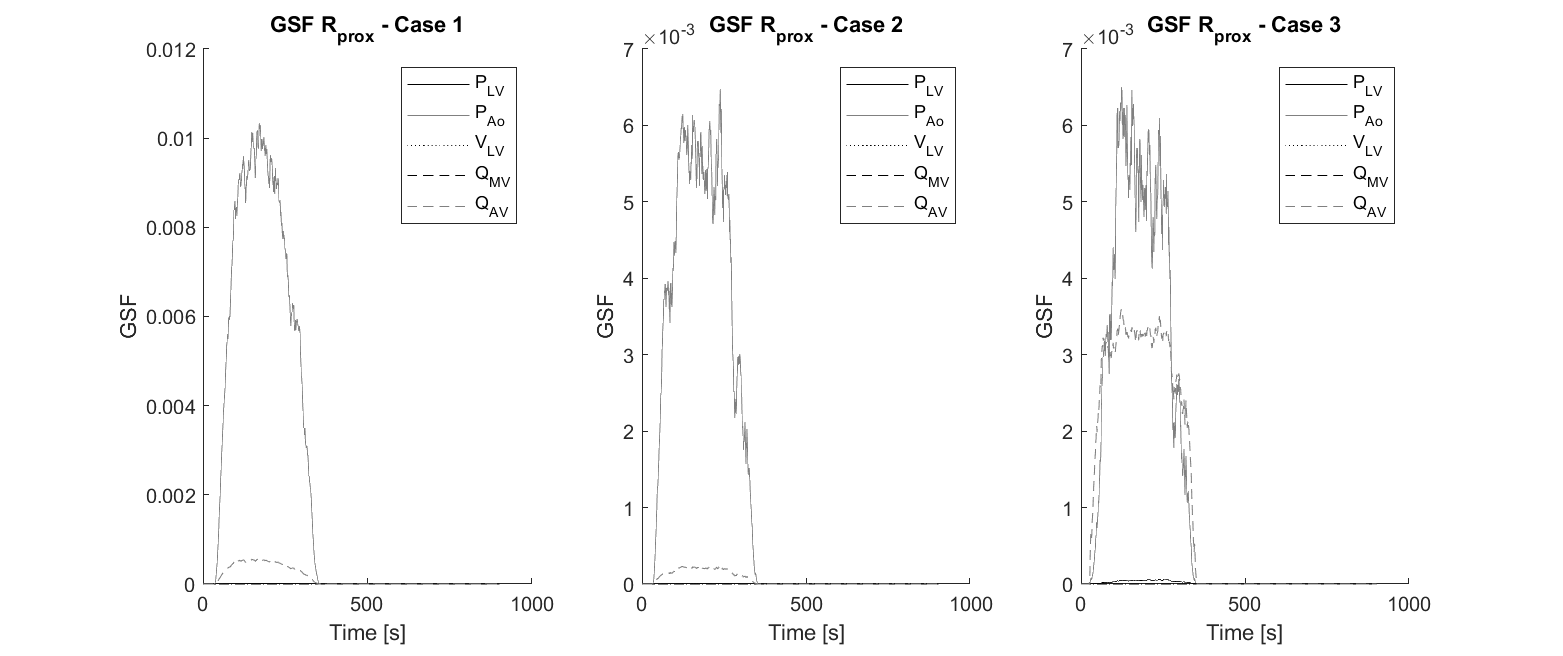

Supplement: Supplementary file 1 — Data S1. Supporting information. [file CNM-36-e3388-s001.zip › Supplementary Images/GSFParam7.png]

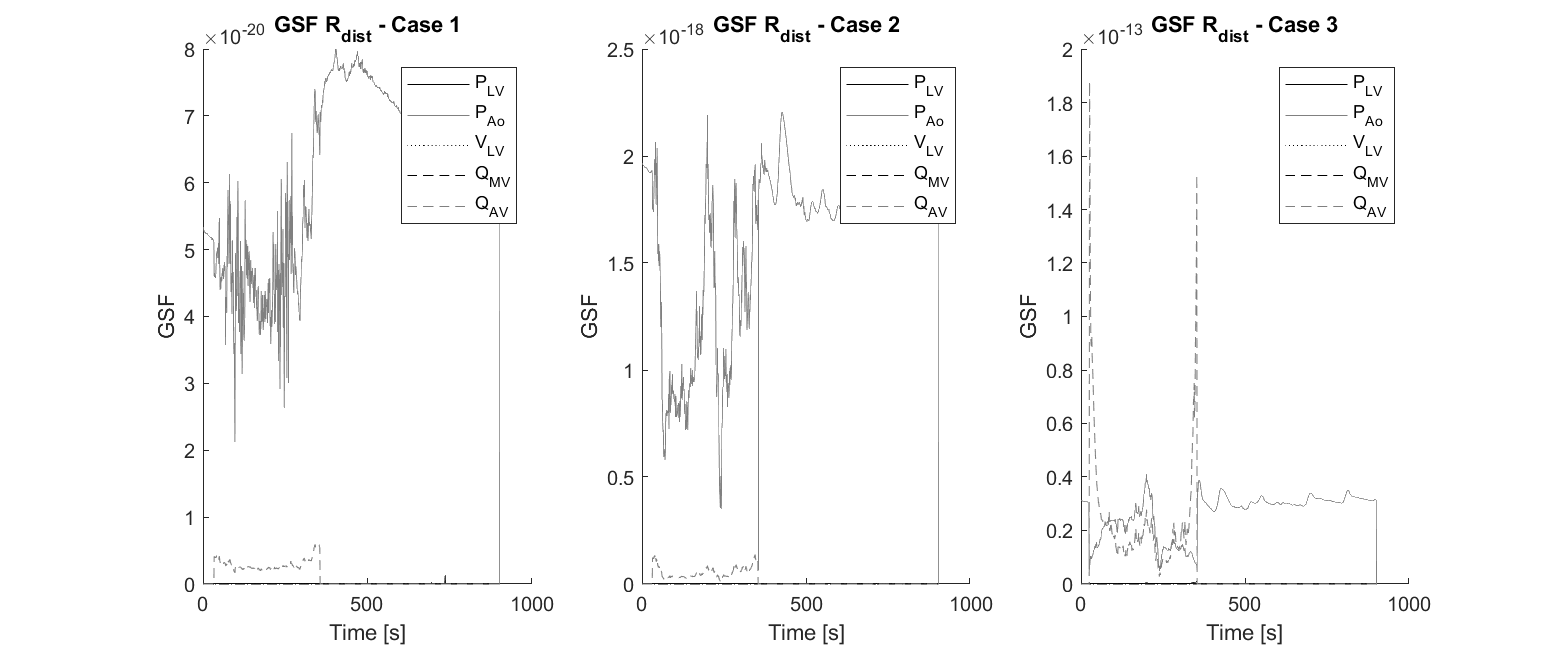

Supplement: Supplementary file 1 — Data S1. Supporting information. [file CNM-36-e3388-s001.zip › Supplementary Images/GSFParam8.png]

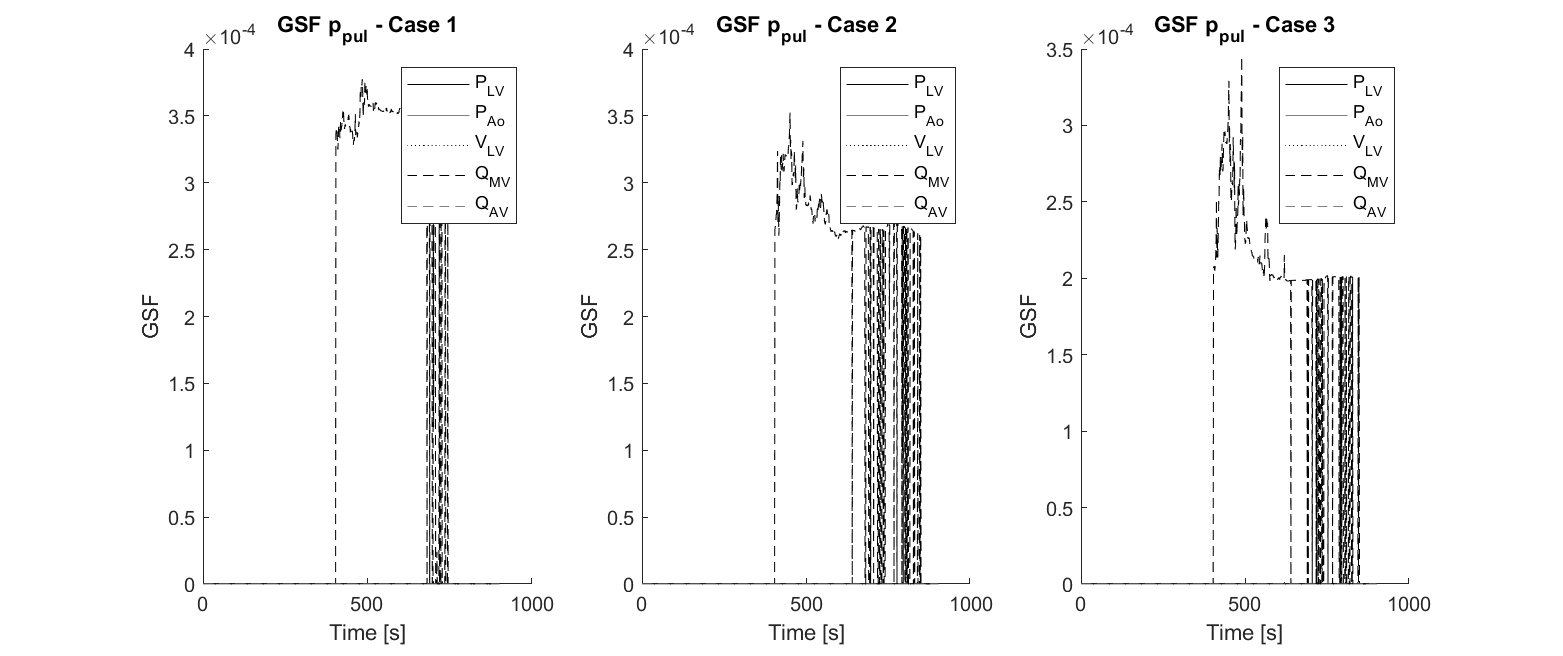

Supplement: Supplementary file 1 — Data S1. Supporting information. [file CNM-36-e3388-s001.zip › Supplementary Images/GSFParam9.png]

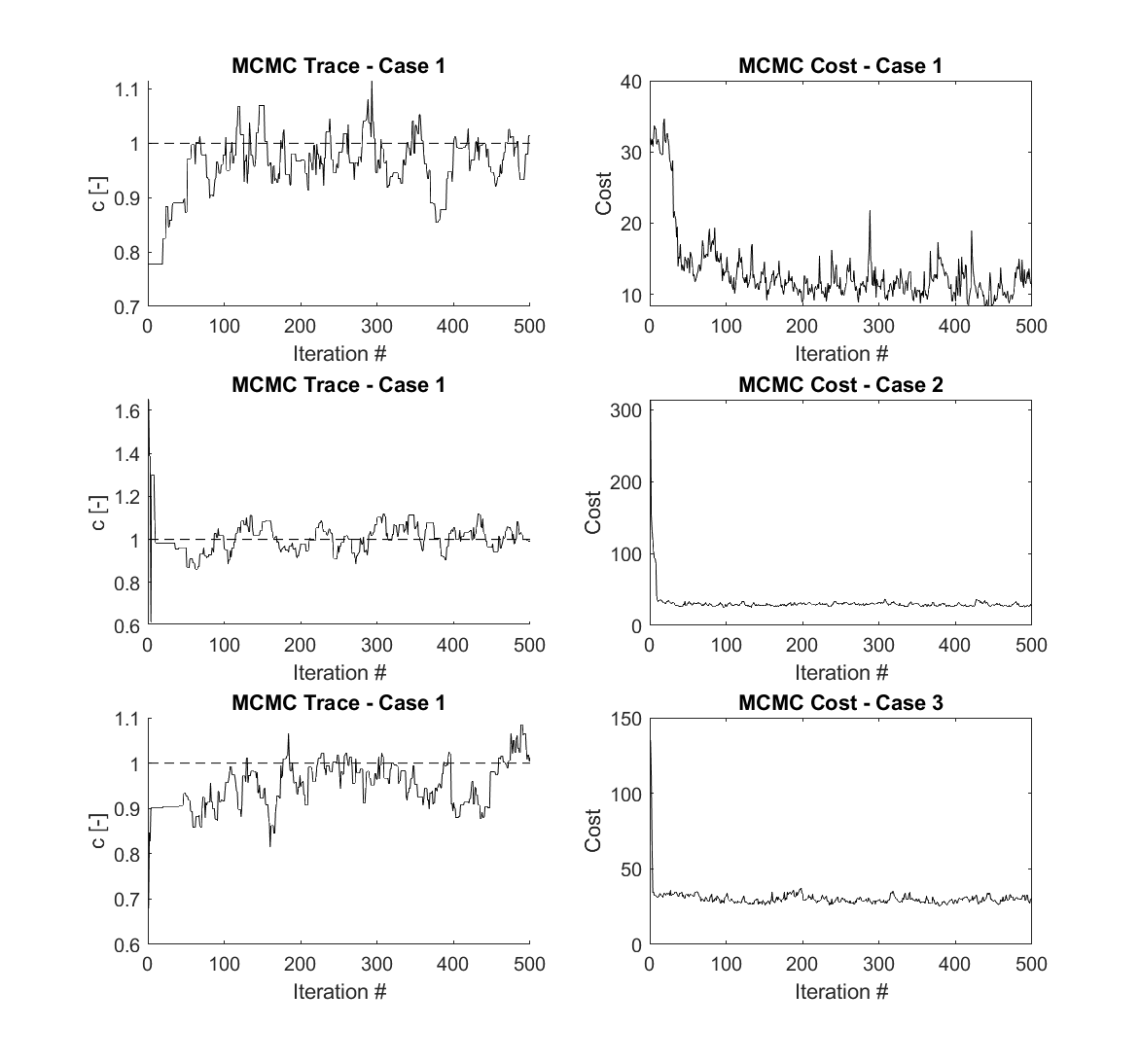

Supplement: Supplementary file 1 — Data S1. Supporting information. [file CNM-36-e3388-s001.zip › Supplementary Images/MCMCParam1.png]

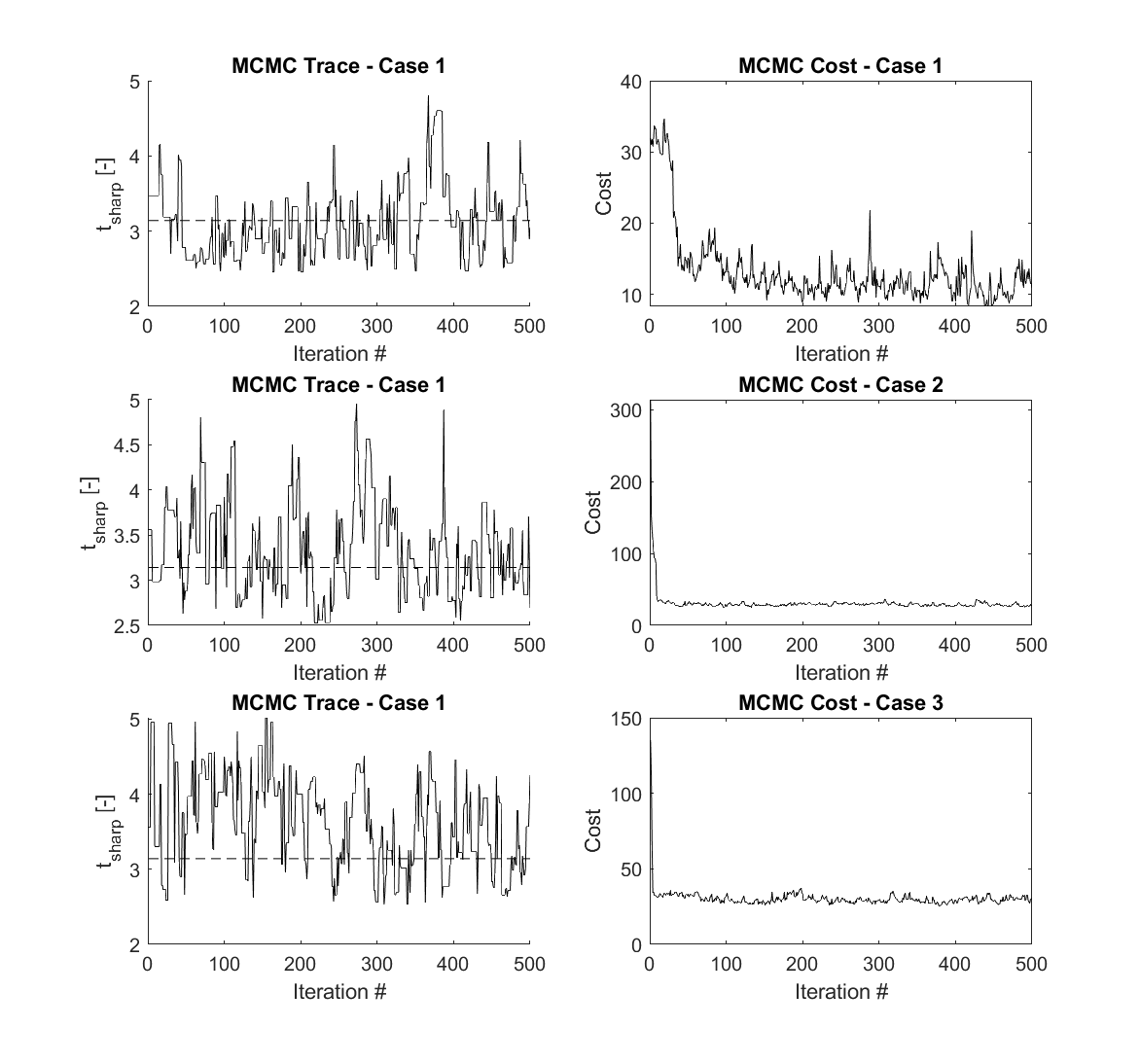

Supplement: Supplementary file 1 — Data S1. Supporting information. [file CNM-36-e3388-s001.zip › Supplementary Images/MCMCParam10.png]

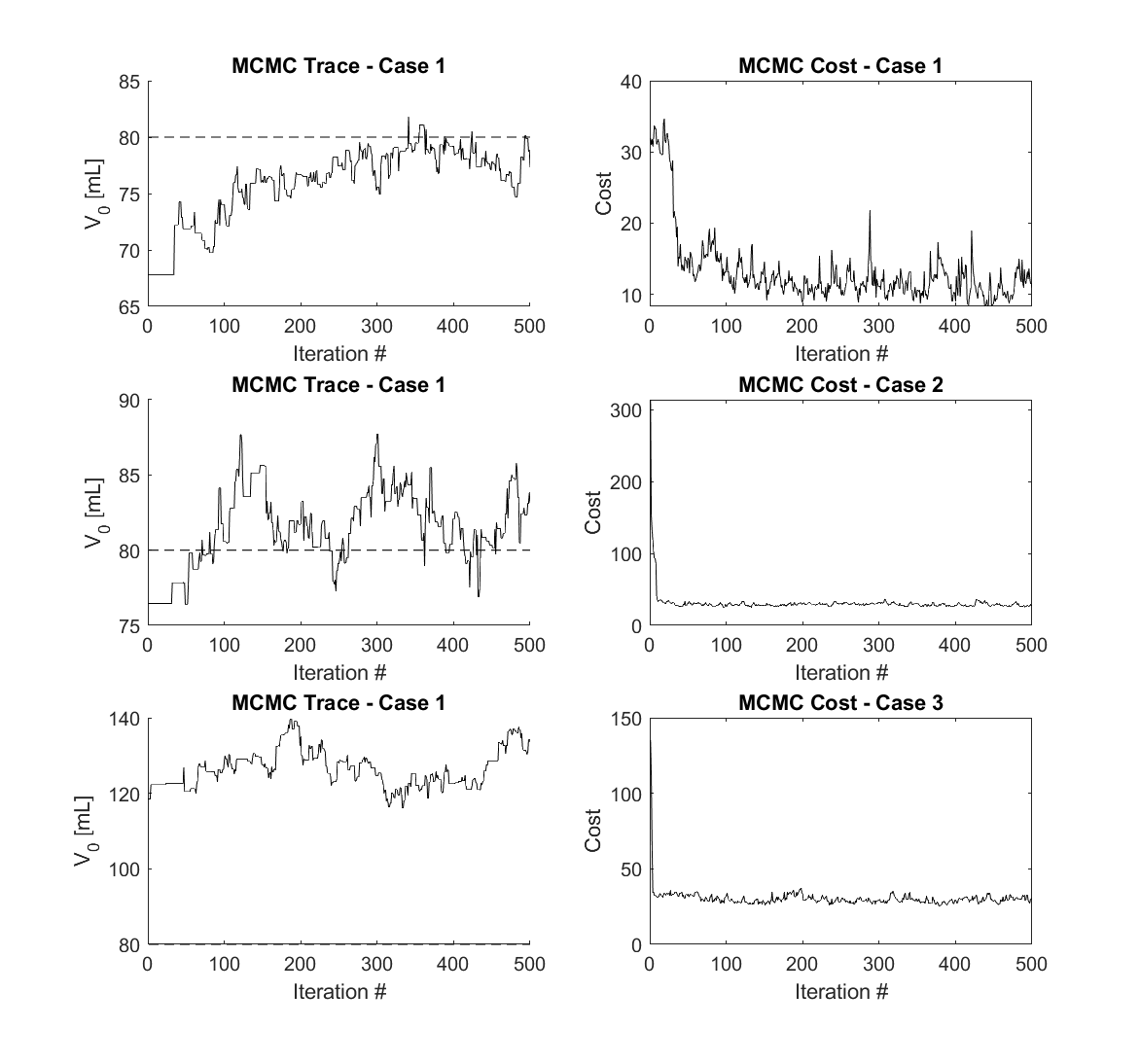

Supplement: Supplementary file 1 — Data S1. Supporting information. [file CNM-36-e3388-s001.zip › Supplementary Images/MCMCParam2.png]

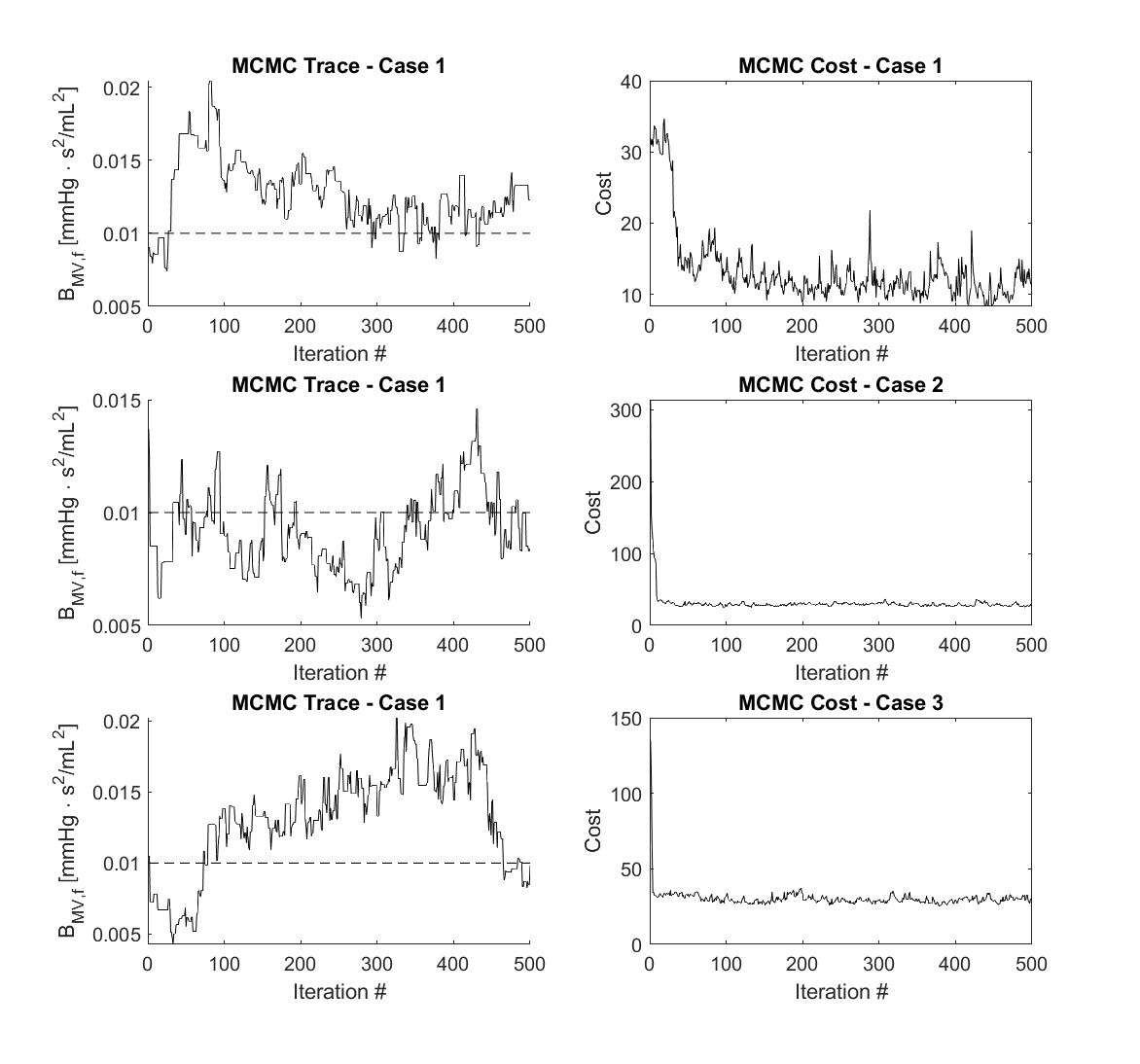

Supplement: Supplementary file 1 — Data S1. Supporting information. [file CNM-36-e3388-s001.zip › Supplementary Images/MCMCParam3.png]

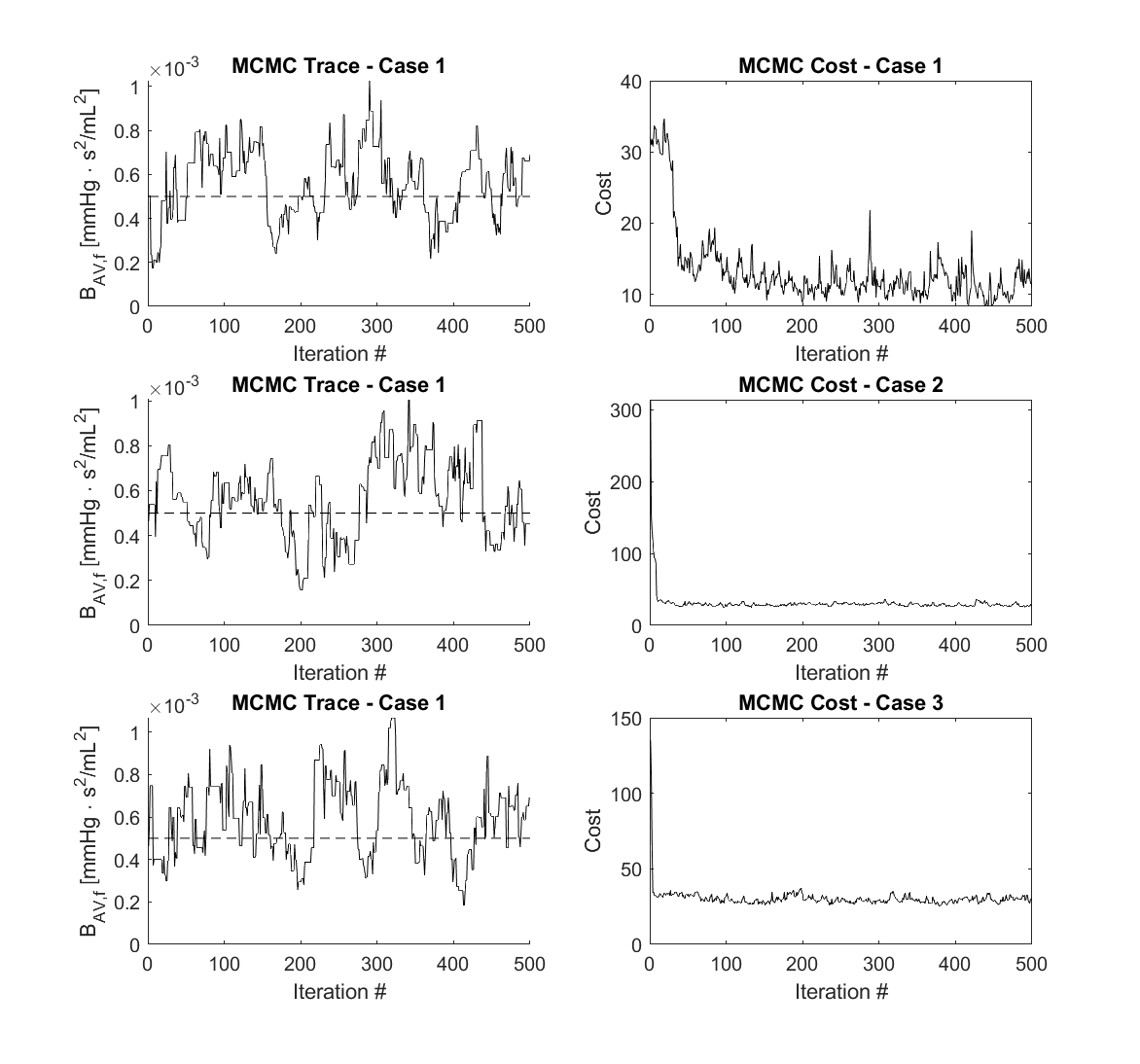

Supplement: Supplementary file 1 — Data S1. Supporting information. [file CNM-36-e3388-s001.zip › Supplementary Images/MCMCParam4.png]

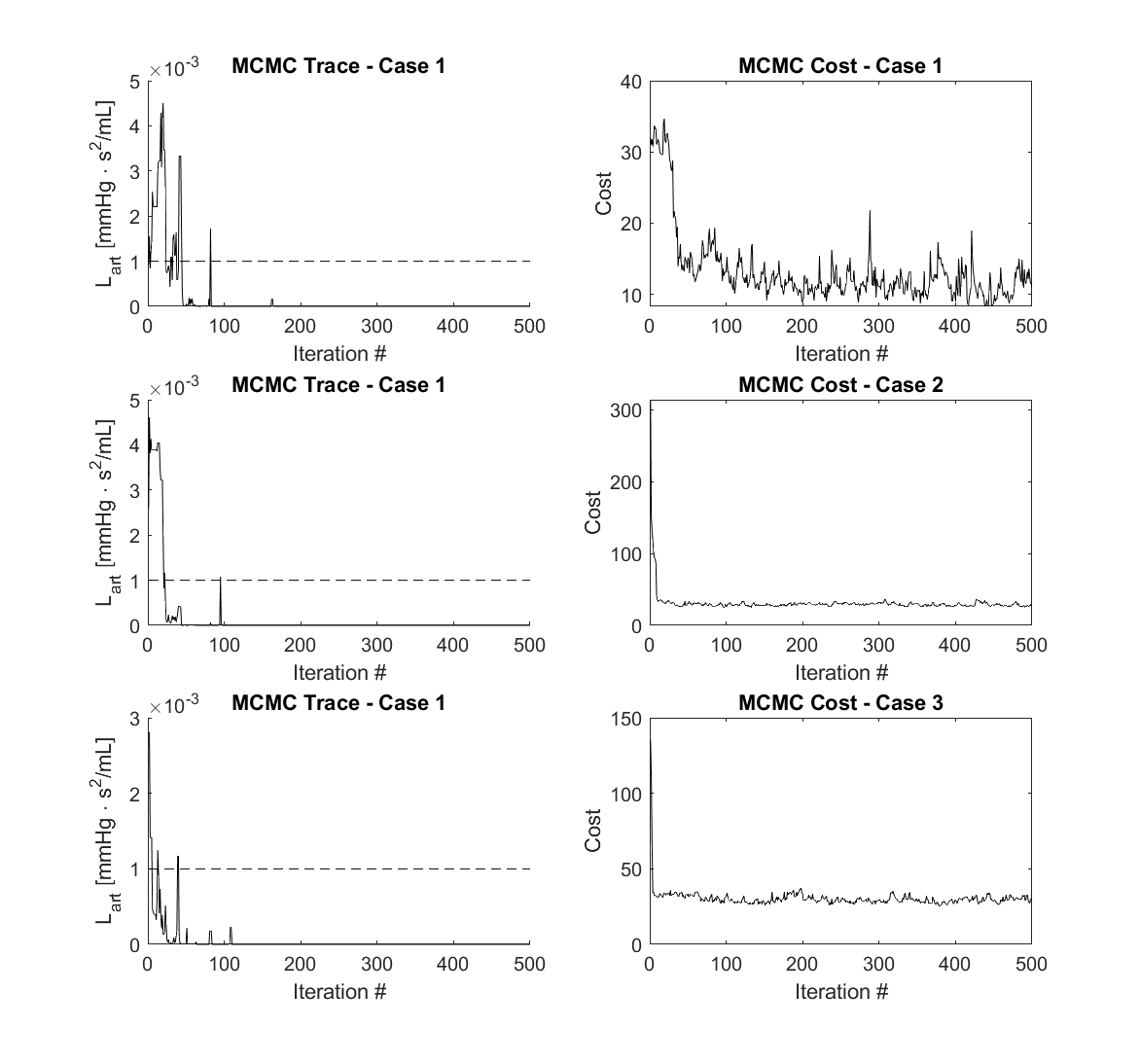

Supplement: Supplementary file 1 — Data S1. Supporting information. [file CNM-36-e3388-s001.zip › Supplementary Images/MCMCParam5.png]

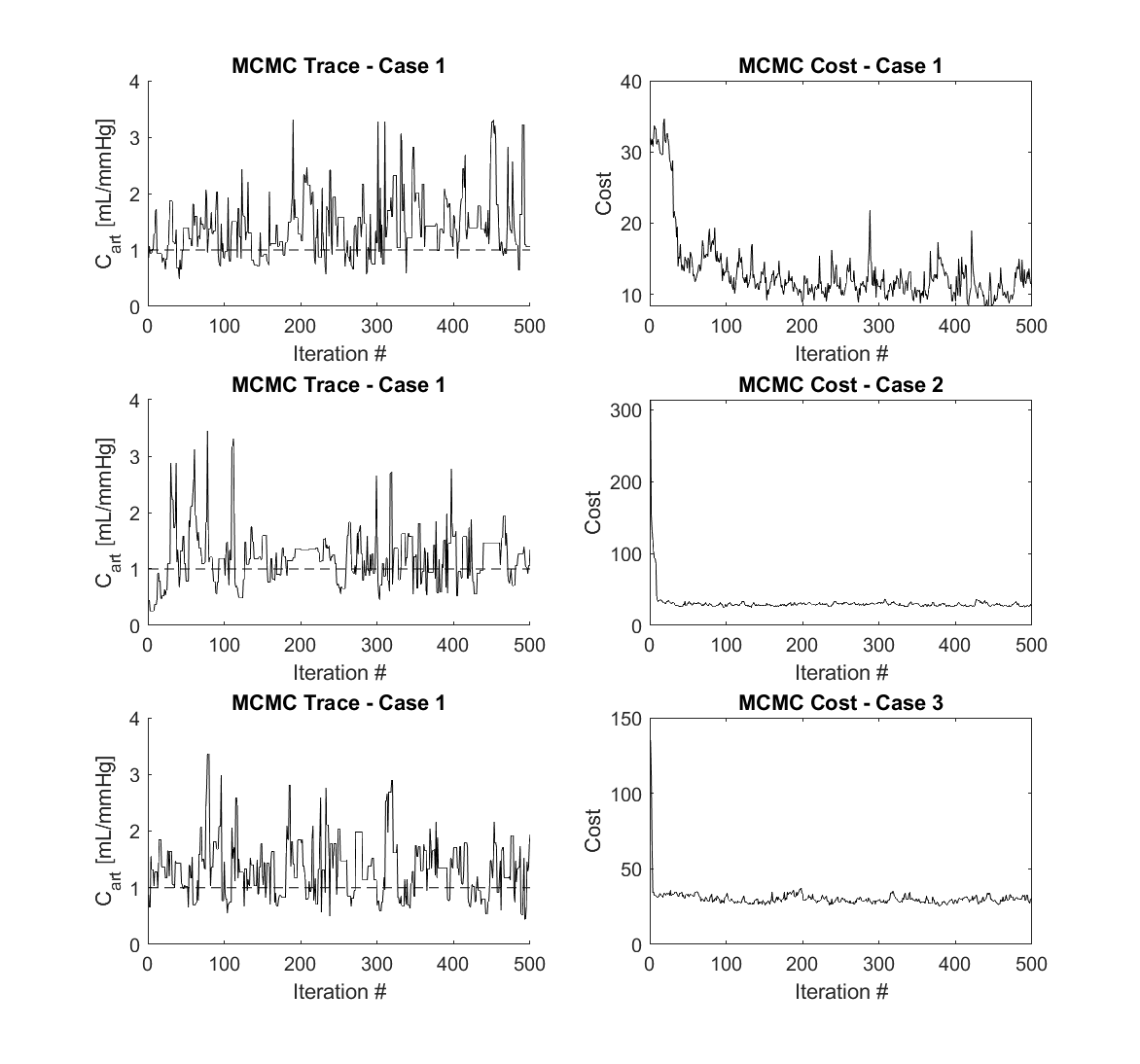

Supplement: Supplementary file 1 — Data S1. Supporting information. [file CNM-36-e3388-s001.zip › Supplementary Images/MCMCParam6.png]

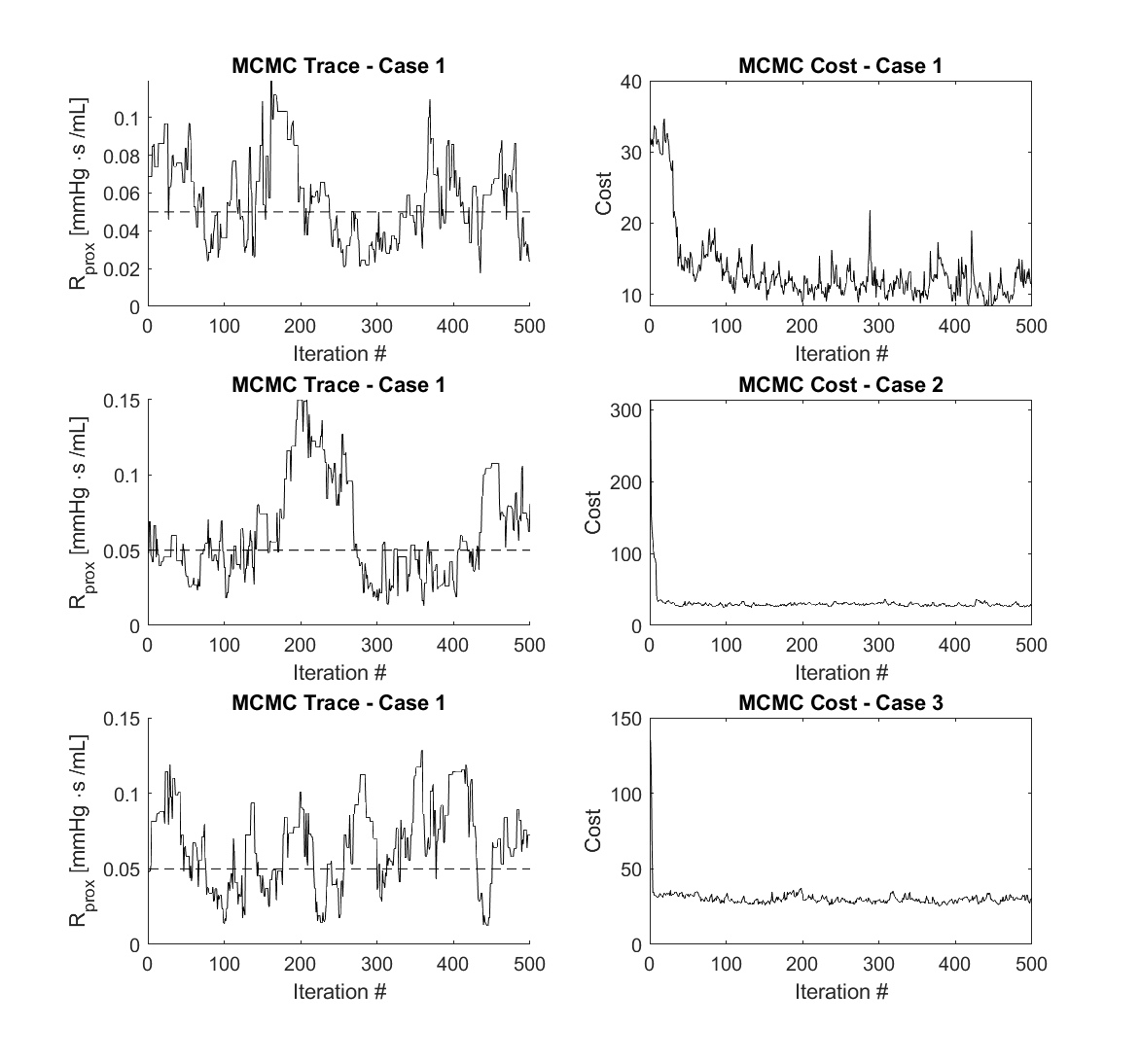

Supplement: Supplementary file 1 — Data S1. Supporting information. [file CNM-36-e3388-s001.zip › Supplementary Images/MCMCParam7.png]

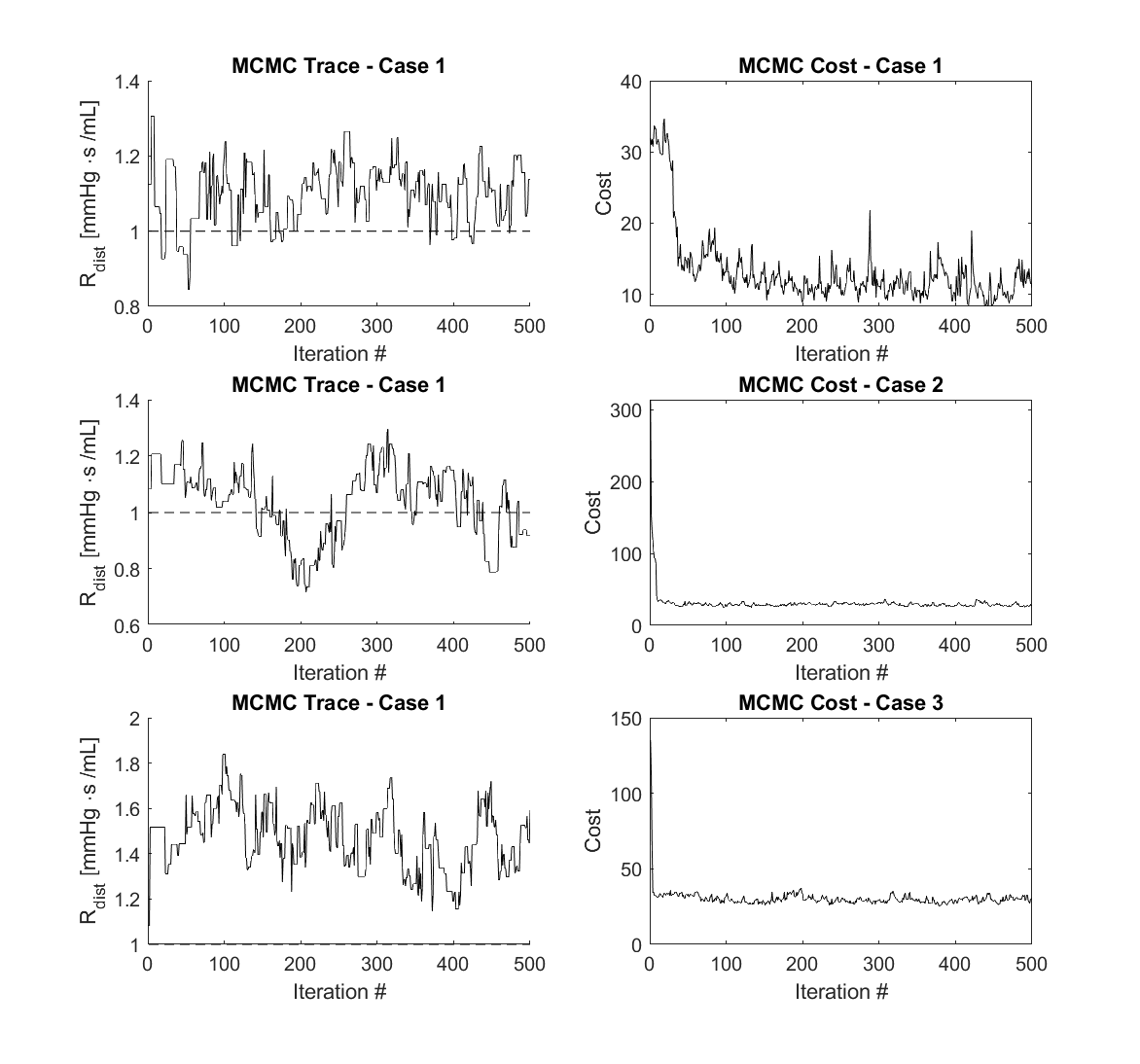

Supplement: Supplementary file 1 — Data S1. Supporting information. [file CNM-36-e3388-s001.zip › Supplementary Images/MCMCParam8.png]

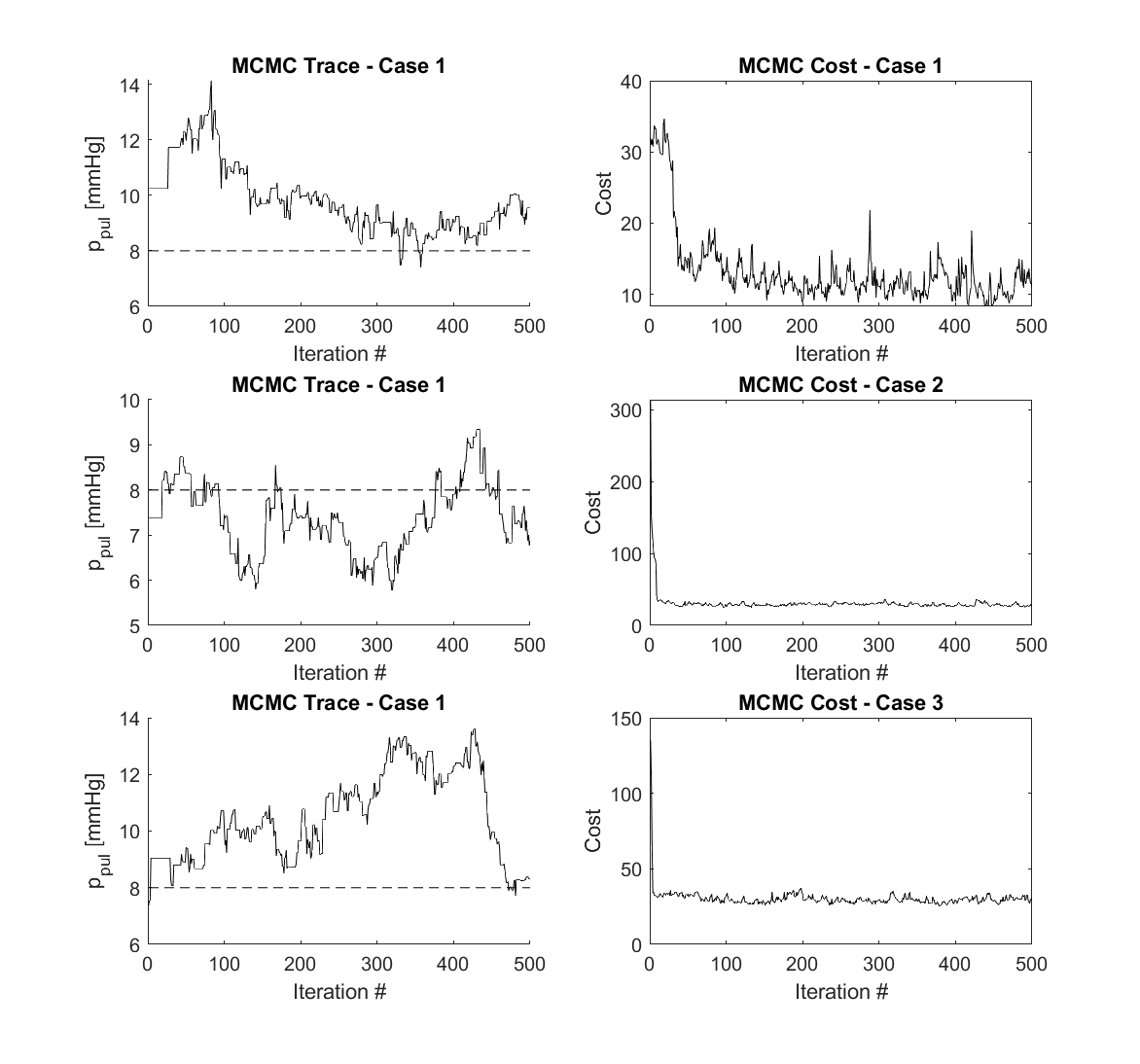

Supplement: Supplementary file 1 — Data S1. Supporting information. [file CNM-36-e3388-s001.zip › Supplementary Images/MCMCParam9.png]

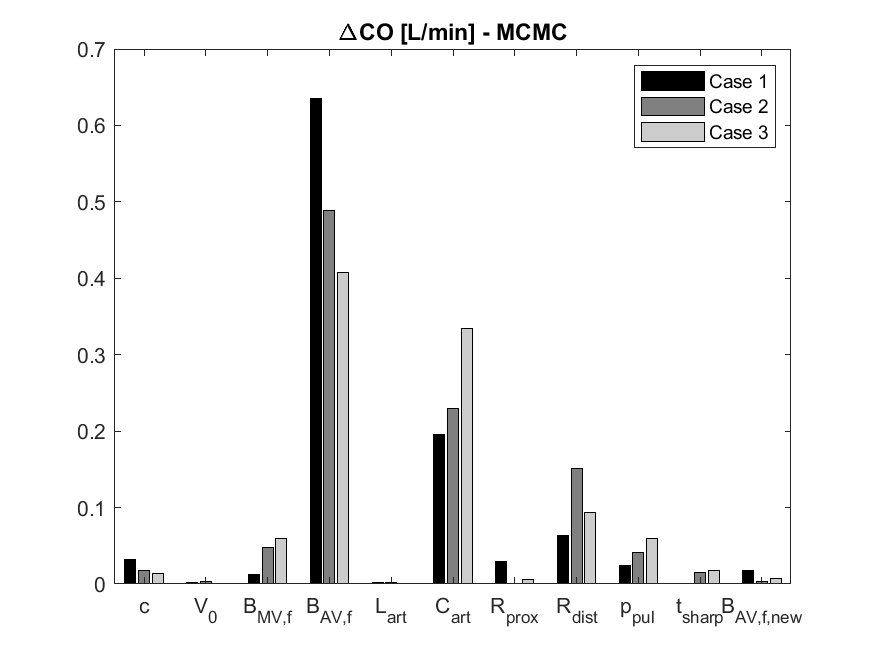

Supplement: Supplementary file 1 — Data S1. Supporting information. [file CNM-36-e3388-s001.zip › Supplementary Images/SobolTotalMCMCOP1.png]

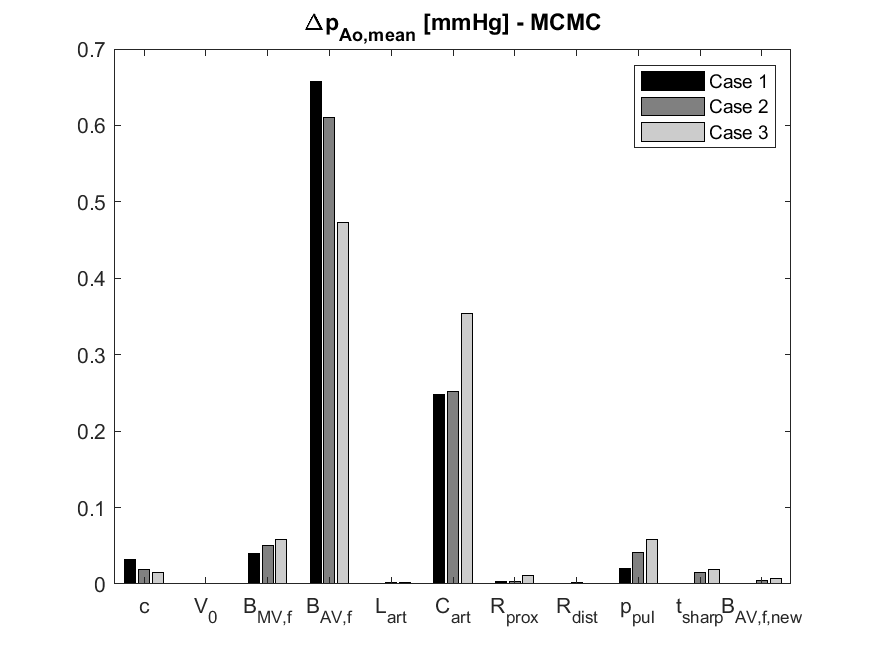

Supplement: Supplementary file 1 — Data S1. Supporting information. [file CNM-36-e3388-s001.zip › Supplementary Images/SobolTotalMCMCOP10.png]

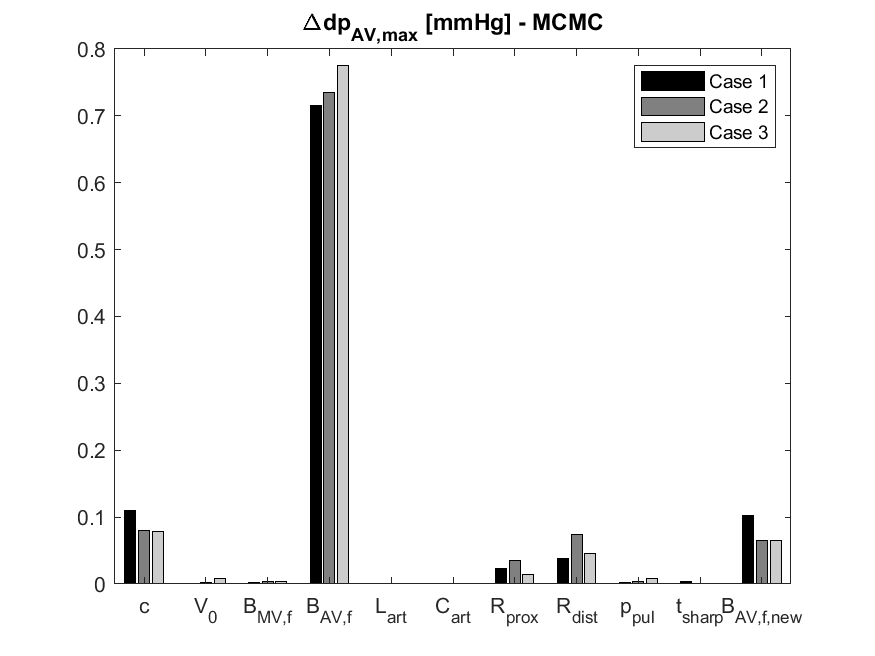

Supplement: Supplementary file 1 — Data S1. Supporting information. [file CNM-36-e3388-s001.zip › Supplementary Images/SobolTotalMCMCOP11.png]

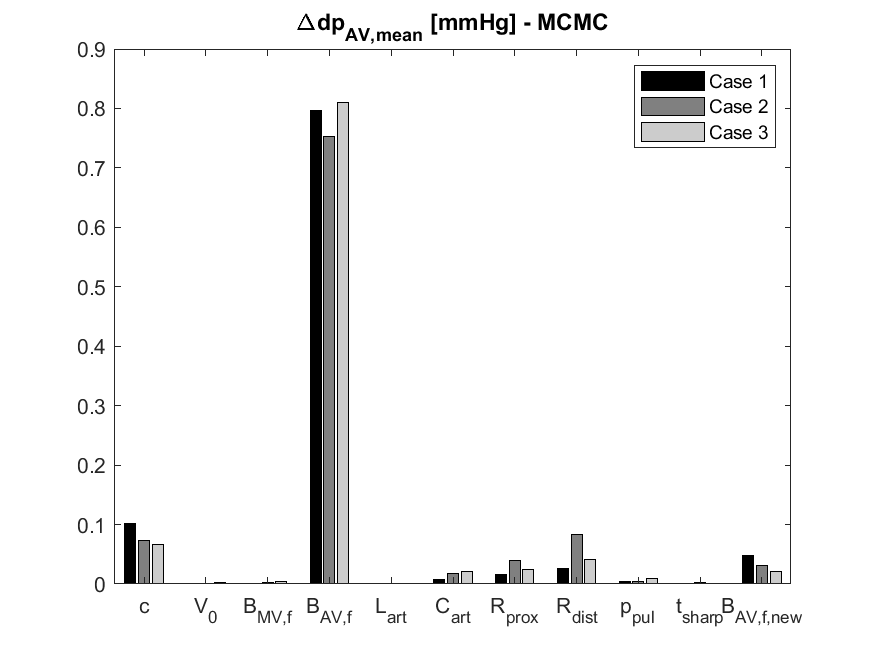

Supplement: Supplementary file 1 — Data S1. Supporting information. [file CNM-36-e3388-s001.zip › Supplementary Images/SobolTotalMCMCOP12.png]

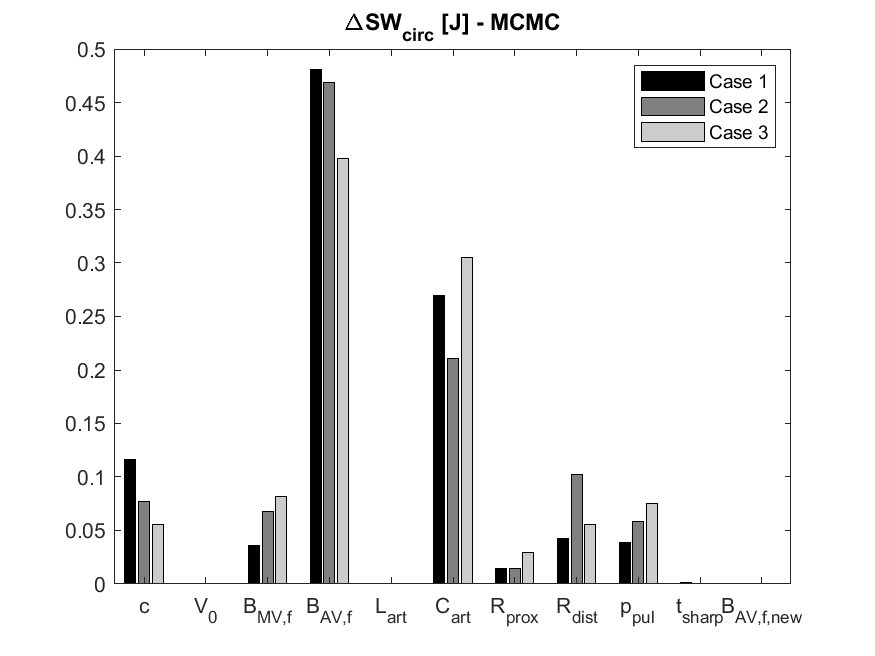

Supplement: Supplementary file 1 — Data S1. Supporting information. [file CNM-36-e3388-s001.zip › Supplementary Images/SobolTotalMCMCOP13.png]

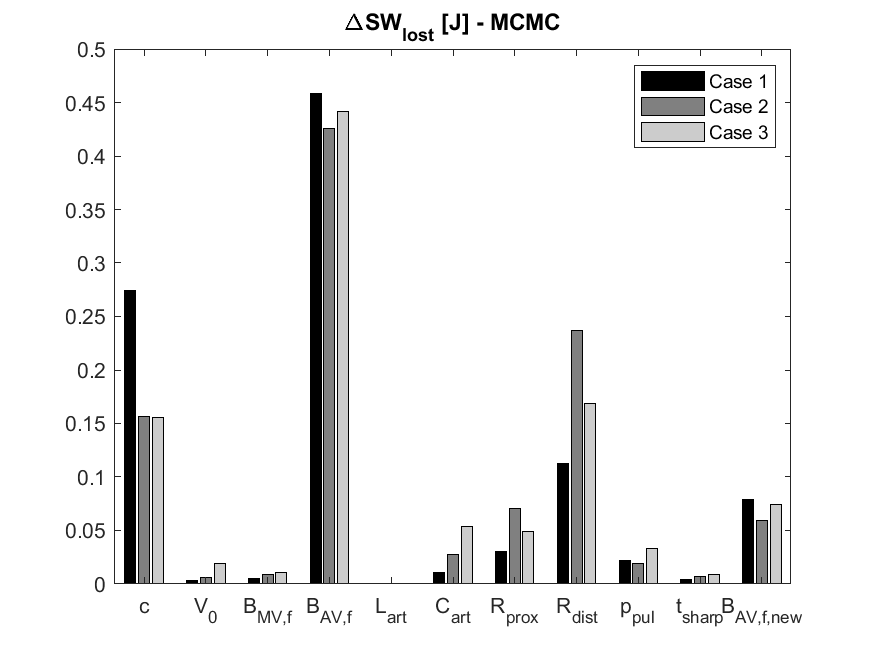

Supplement: Supplementary file 1 — Data S1. Supporting information. [file CNM-36-e3388-s001.zip › Supplementary Images/SobolTotalMCMCOP14.png]

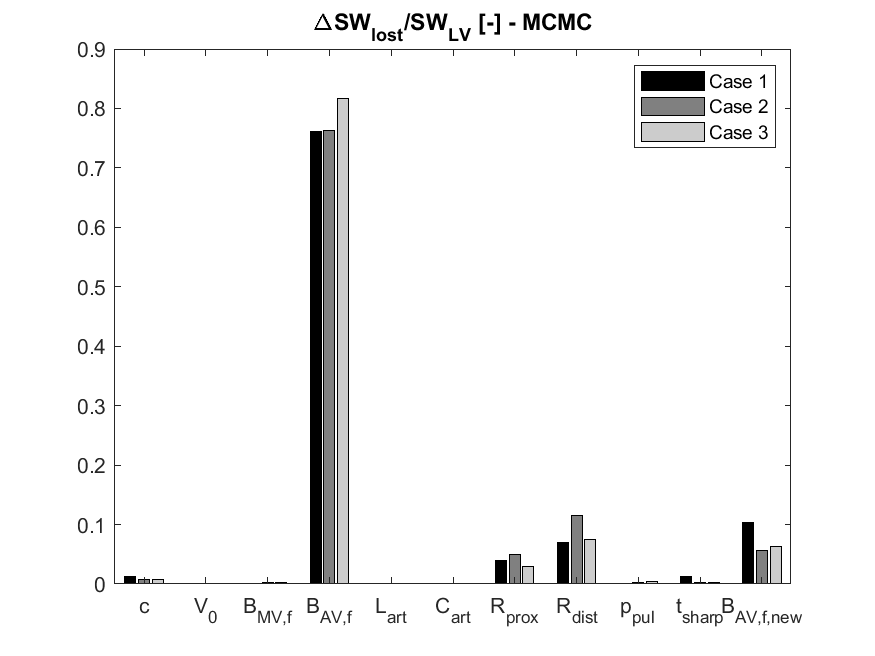

Supplement: Supplementary file 1 — Data S1. Supporting information. [file CNM-36-e3388-s001.zip › Supplementary Images/SobolTotalMCMCOP15.png]

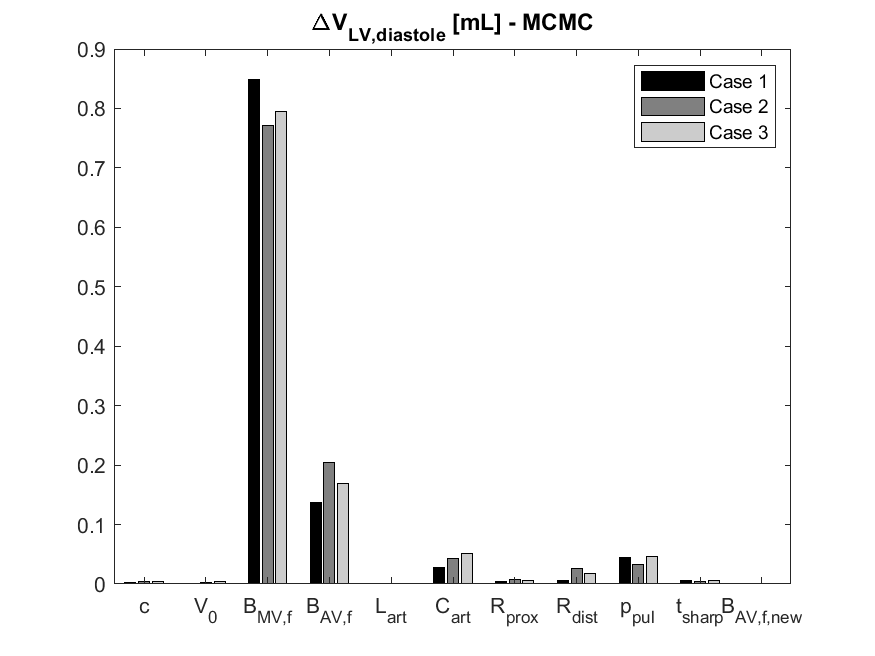

Supplement: Supplementary file 1 — Data S1. Supporting information. [file CNM-36-e3388-s001.zip › Supplementary Images/SobolTotalMCMCOP2.png]

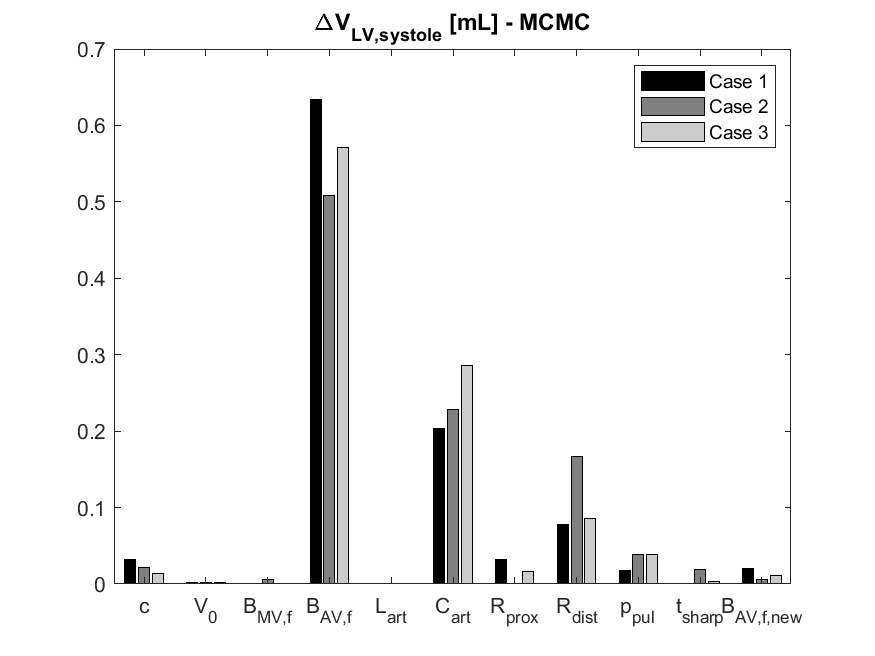

Supplement: Supplementary file 1 — Data S1. Supporting information. [file CNM-36-e3388-s001.zip › Supplementary Images/SobolTotalMCMCOP3.png]

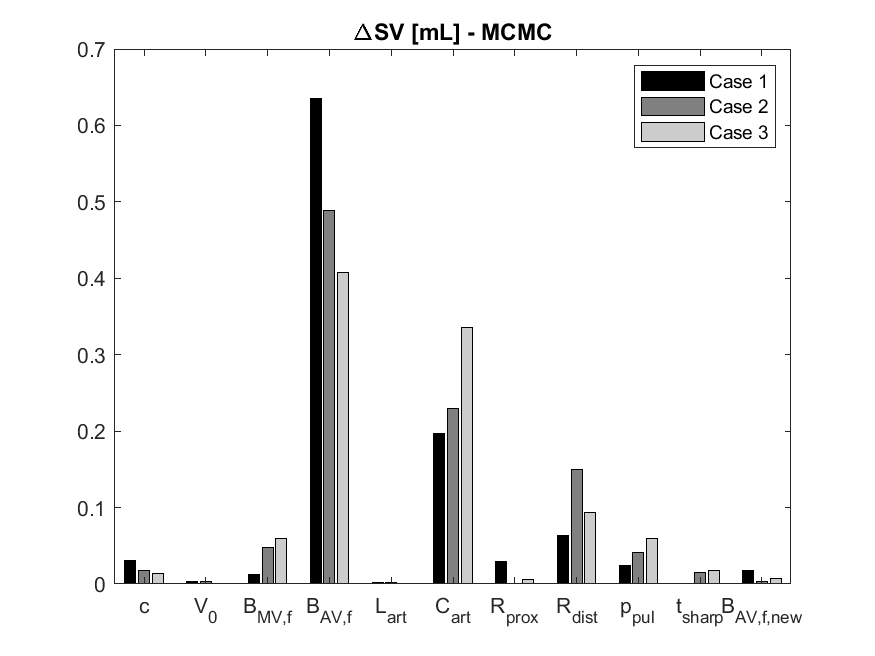

Supplement: Supplementary file 1 — Data S1. Supporting information. [file CNM-36-e3388-s001.zip › Supplementary Images/SobolTotalMCMCOP4.png]

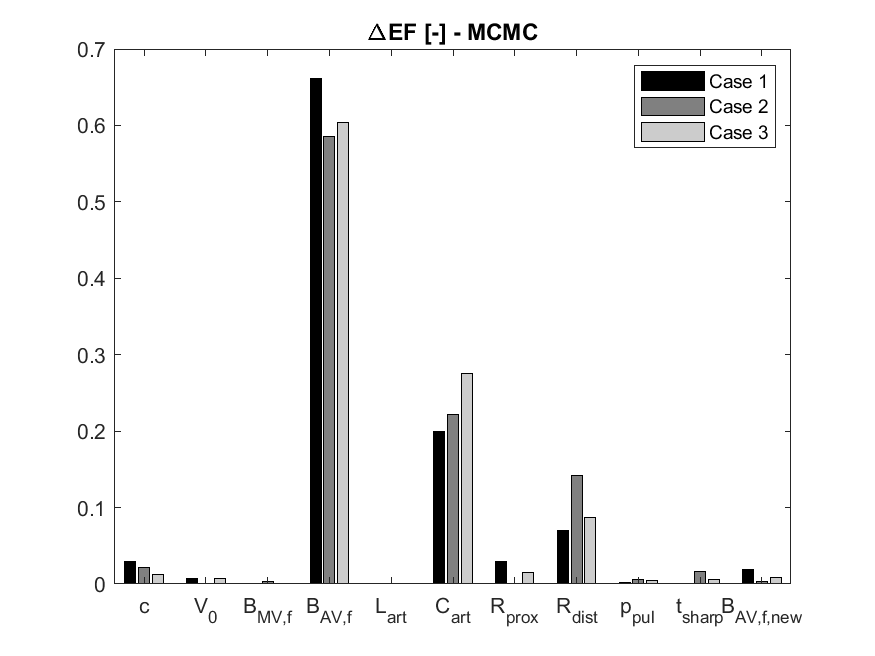

Supplement: Supplementary file 1 — Data S1. Supporting information. [file CNM-36-e3388-s001.zip › Supplementary Images/SobolTotalMCMCOP5.png]

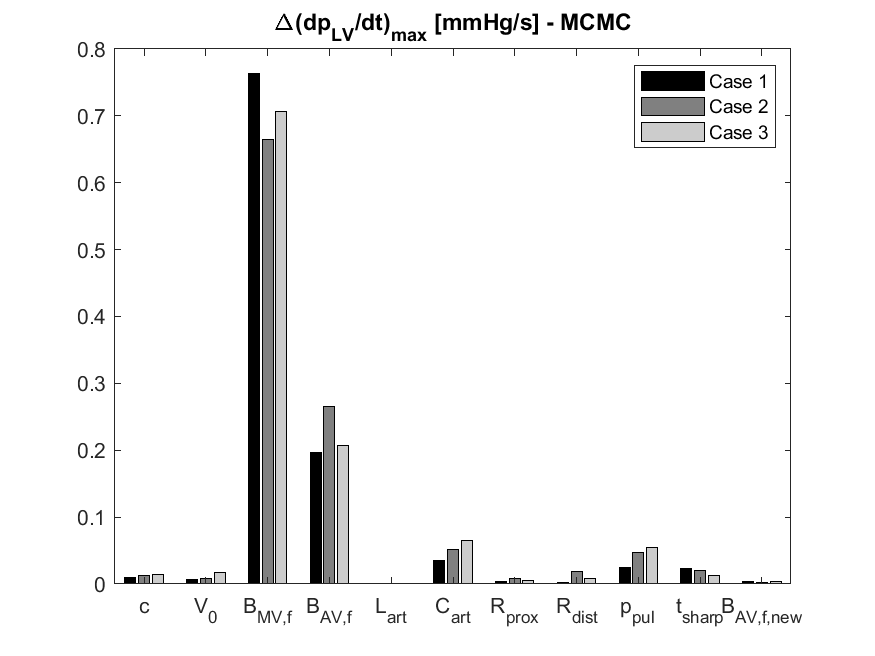

Supplement: Supplementary file 1 — Data S1. Supporting information. [file CNM-36-e3388-s001.zip › Supplementary Images/SobolTotalMCMCOP6.png]

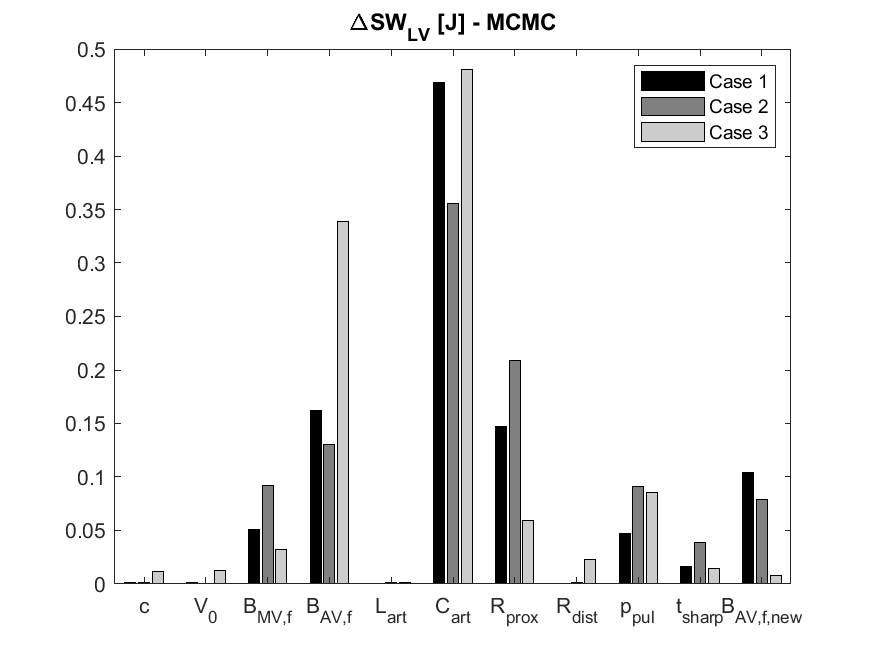

Supplement: Supplementary file 1 — Data S1. Supporting information. [file CNM-36-e3388-s001.zip › Supplementary Images/SobolTotalMCMCOP7.png]

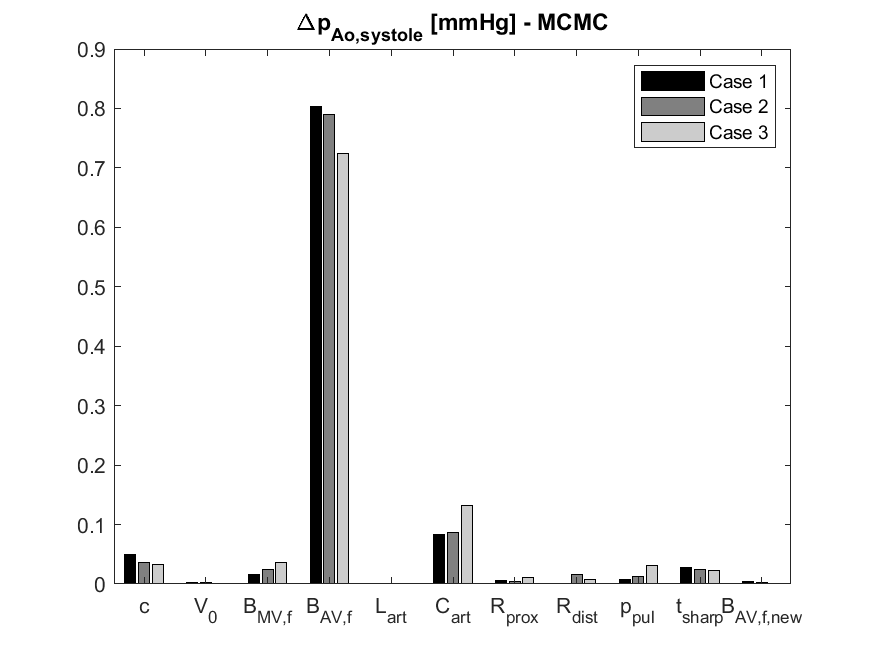

Supplement: Supplementary file 1 — Data S1. Supporting information. [file CNM-36-e3388-s001.zip › Supplementary Images/SobolTotalMCMCOP8.png]

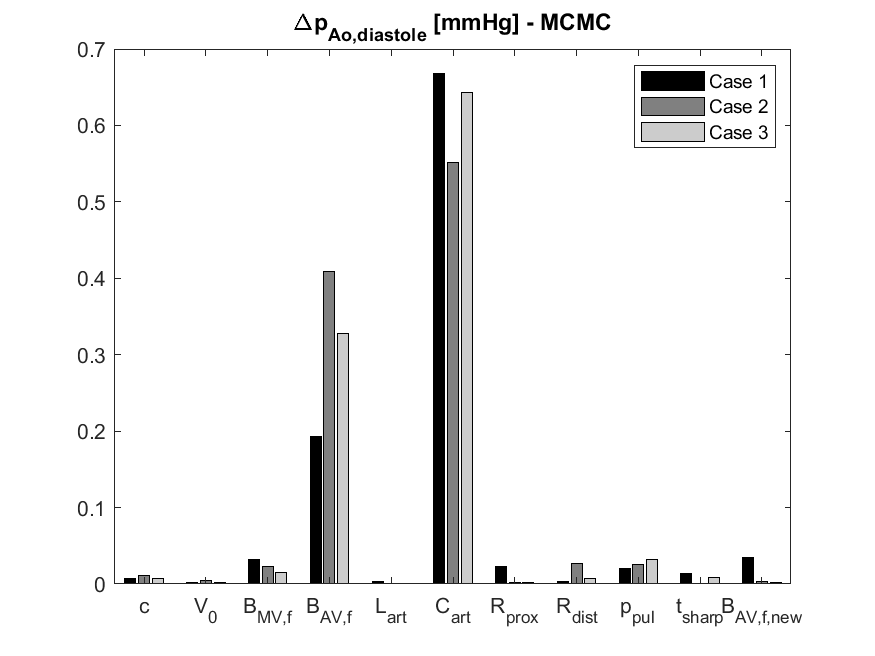

Supplement: Supplementary file 1 — Data S1. Supporting information. [file CNM-36-e3388-s001.zip › Supplementary Images/SobolTotalMCMCOP9.png]

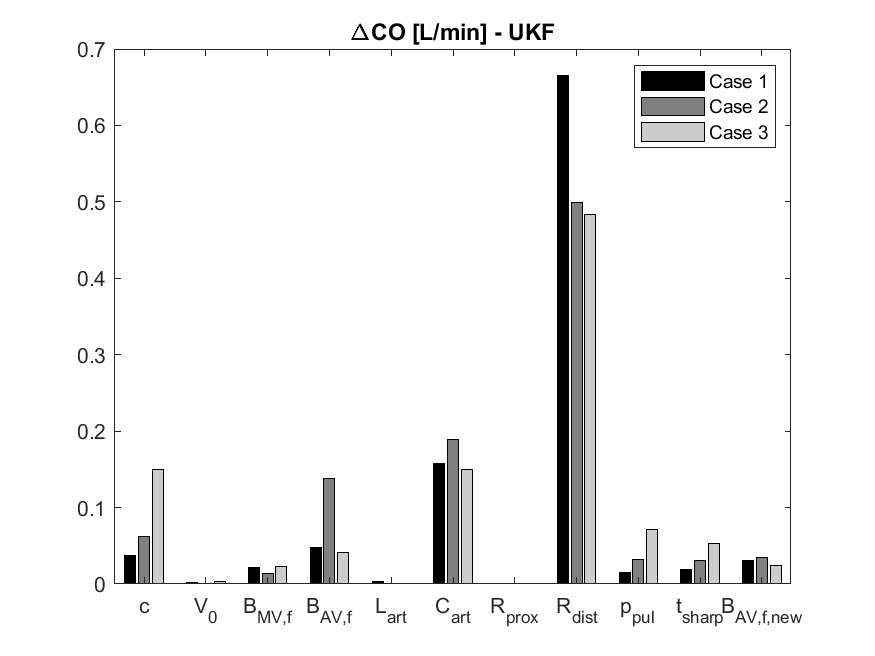

Supplement: Supplementary file 1 — Data S1. Supporting information. [file CNM-36-e3388-s001.zip › Supplementary Images/SobolTotalUKFOP1.png]

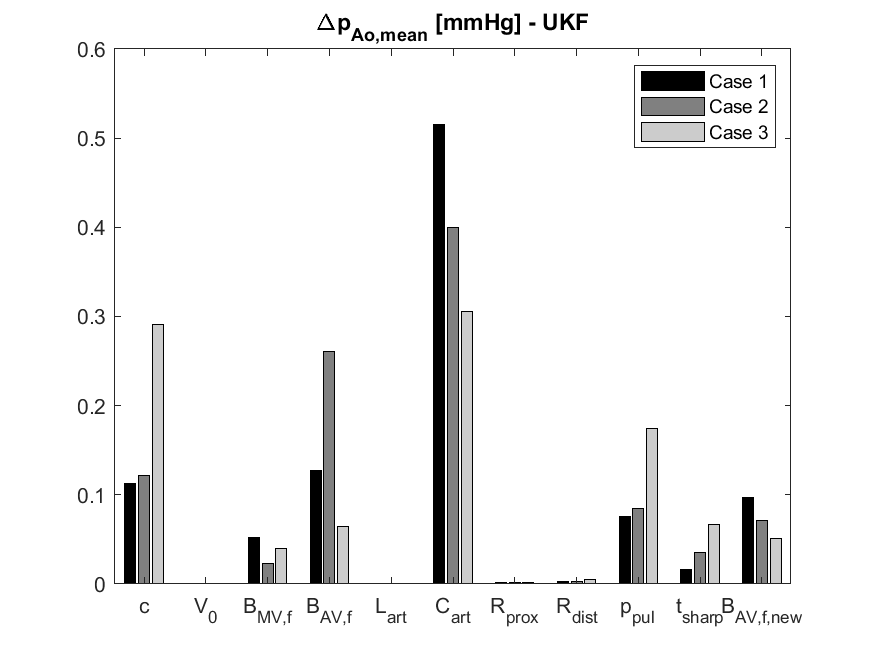

Supplement: Supplementary file 1 — Data S1. Supporting information. [file CNM-36-e3388-s001.zip › Supplementary Images/SobolTotalUKFOP10.png]

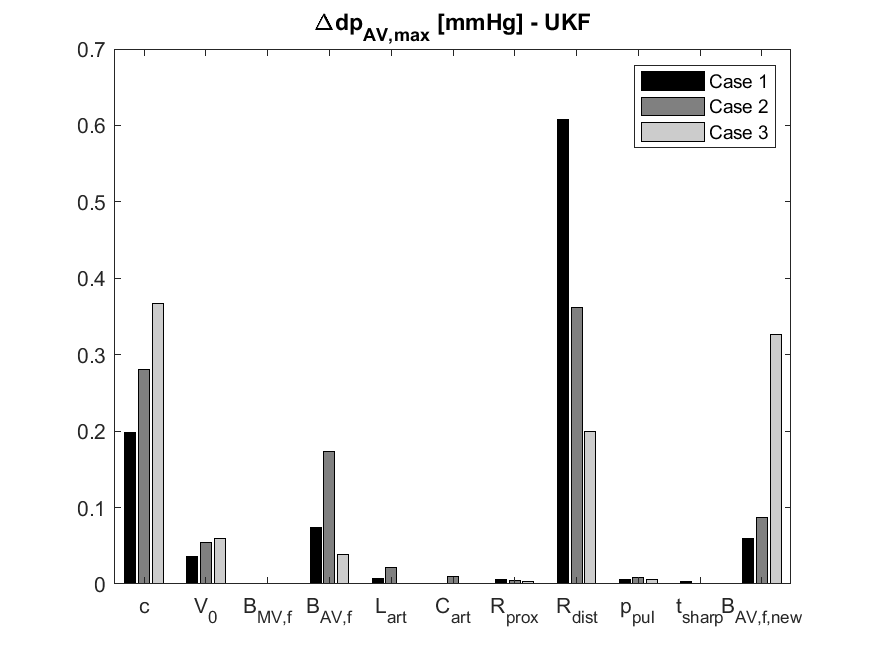

Supplement: Supplementary file 1 — Data S1. Supporting information. [file CNM-36-e3388-s001.zip › Supplementary Images/SobolTotalUKFOP11.png]

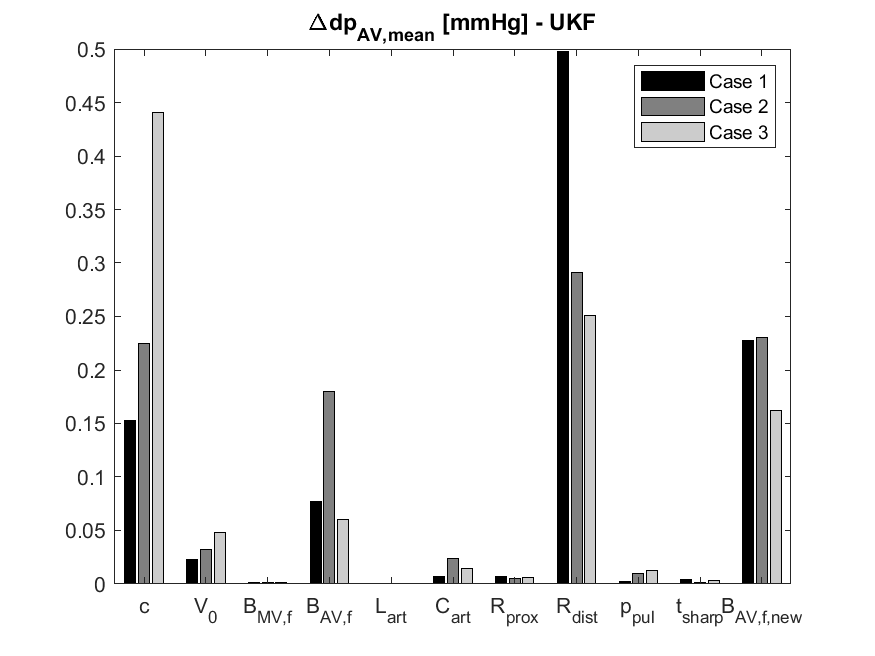

Supplement: Supplementary file 1 — Data S1. Supporting information. [file CNM-36-e3388-s001.zip › Supplementary Images/SobolTotalUKFOP12.png]

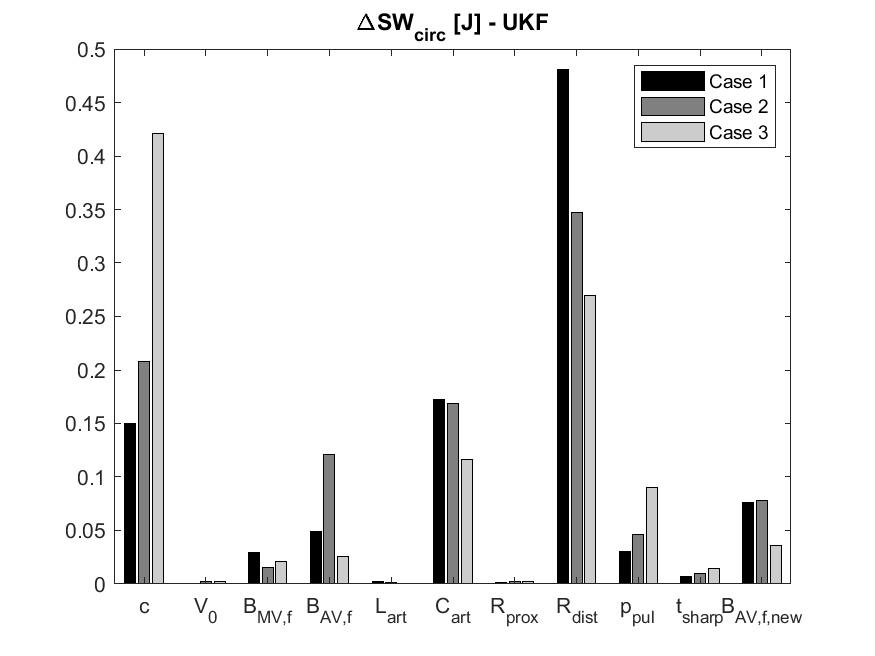

Supplement: Supplementary file 1 — Data S1. Supporting information. [file CNM-36-e3388-s001.zip › Supplementary Images/SobolTotalUKFOP13.png]

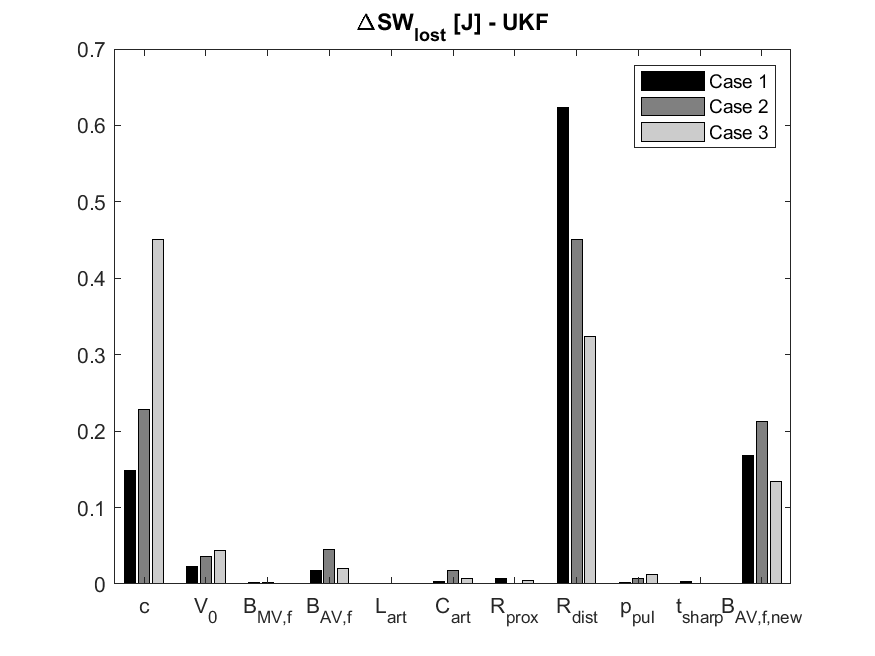

Supplement: Supplementary file 1 — Data S1. Supporting information. [file CNM-36-e3388-s001.zip › Supplementary Images/SobolTotalUKFOP14.png]

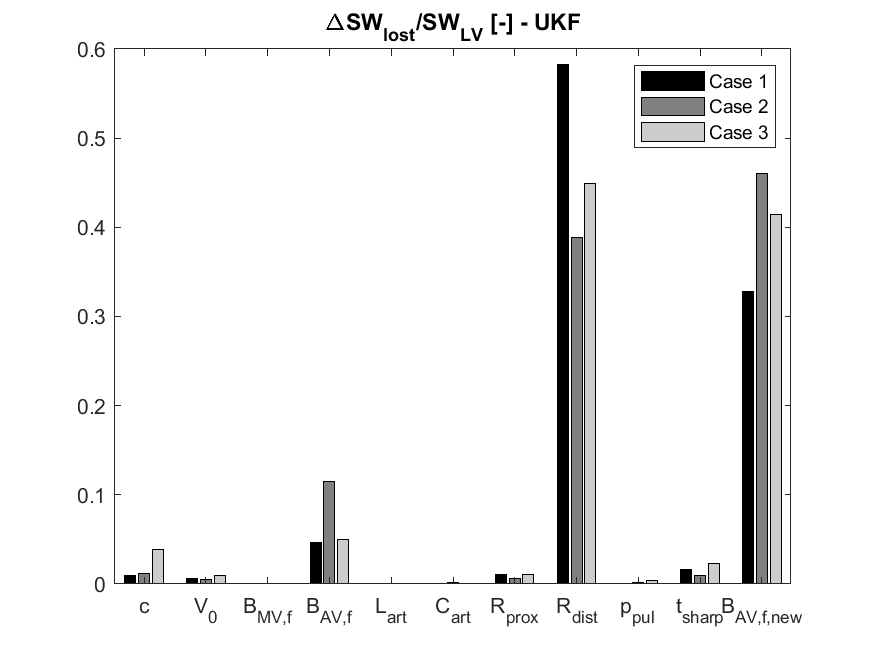

Supplement: Supplementary file 1 — Data S1. Supporting information. [file CNM-36-e3388-s001.zip › Supplementary Images/SobolTotalUKFOP15.png]

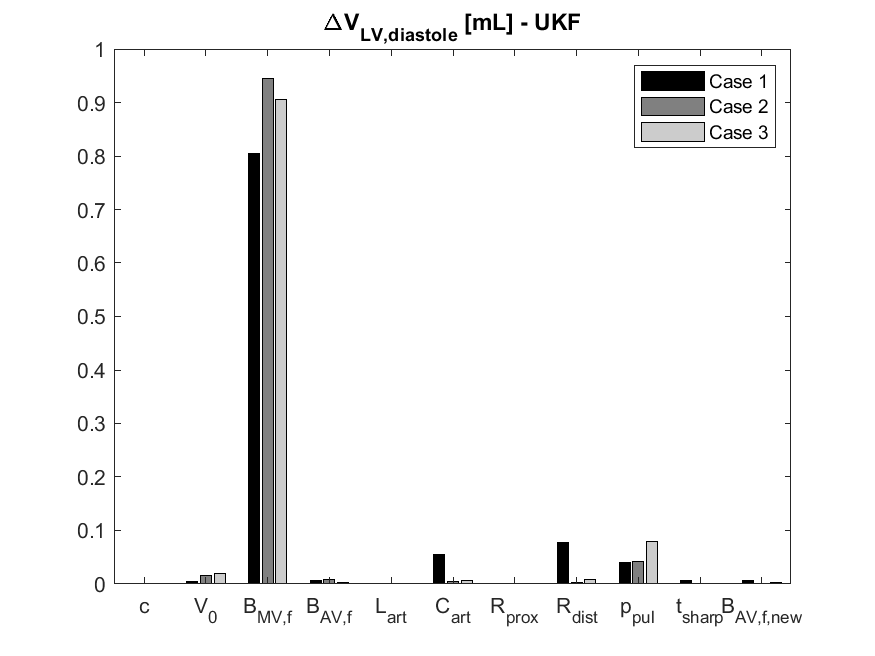

Supplement: Supplementary file 1 — Data S1. Supporting information. [file CNM-36-e3388-s001.zip › Supplementary Images/SobolTotalUKFOP2.png]

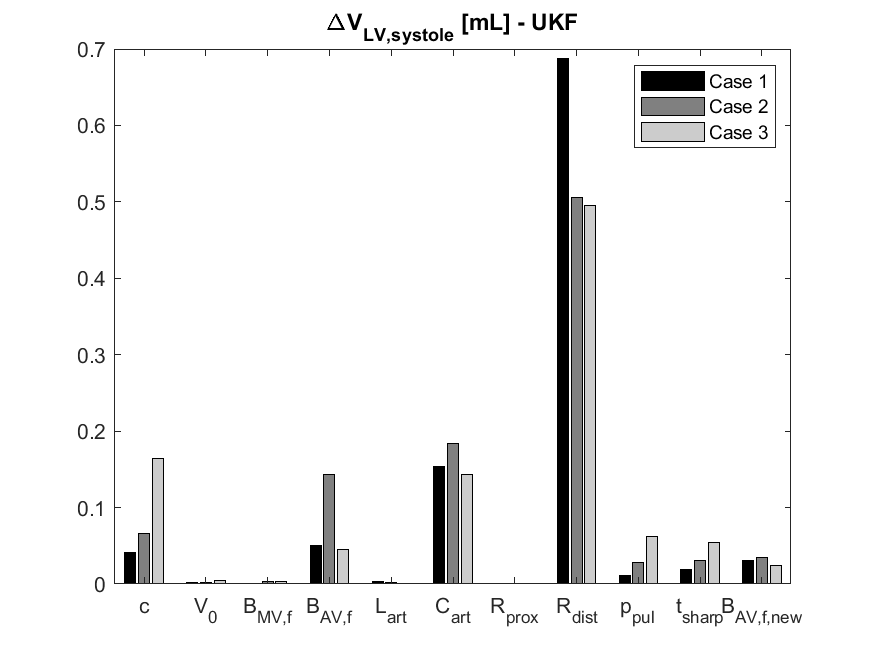

Supplement: Supplementary file 1 — Data S1. Supporting information. [file CNM-36-e3388-s001.zip › Supplementary Images/SobolTotalUKFOP3.png]

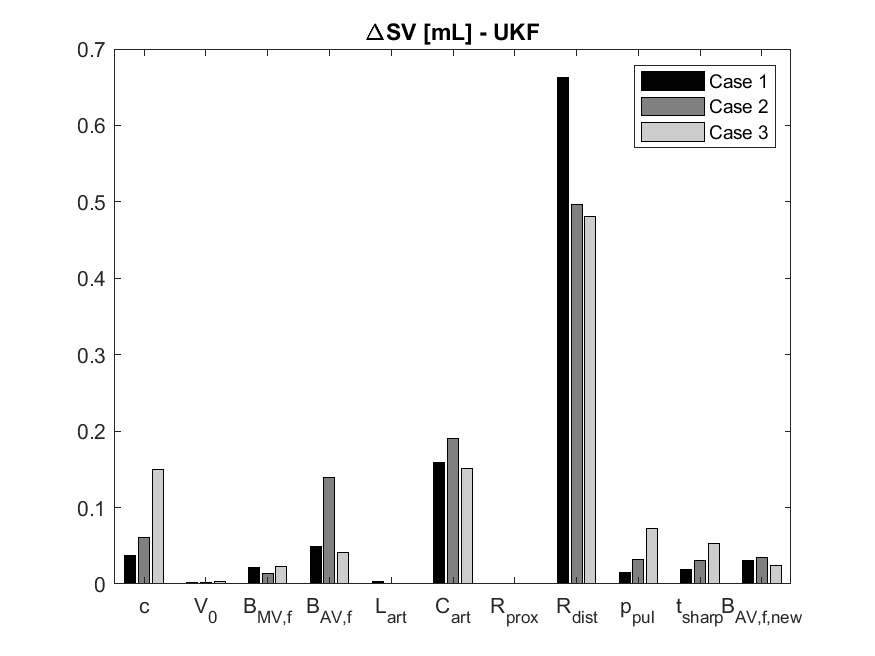

Supplement: Supplementary file 1 — Data S1. Supporting information. [file CNM-36-e3388-s001.zip › Supplementary Images/SobolTotalUKFOP4.png]

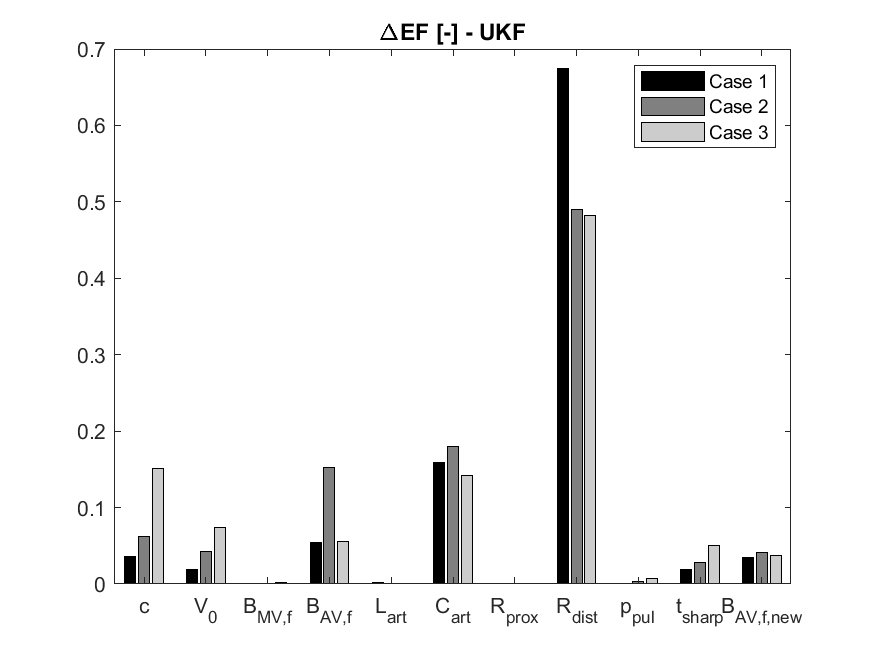

Supplement: Supplementary file 1 — Data S1. Supporting information. [file CNM-36-e3388-s001.zip › Supplementary Images/SobolTotalUKFOP5.png]

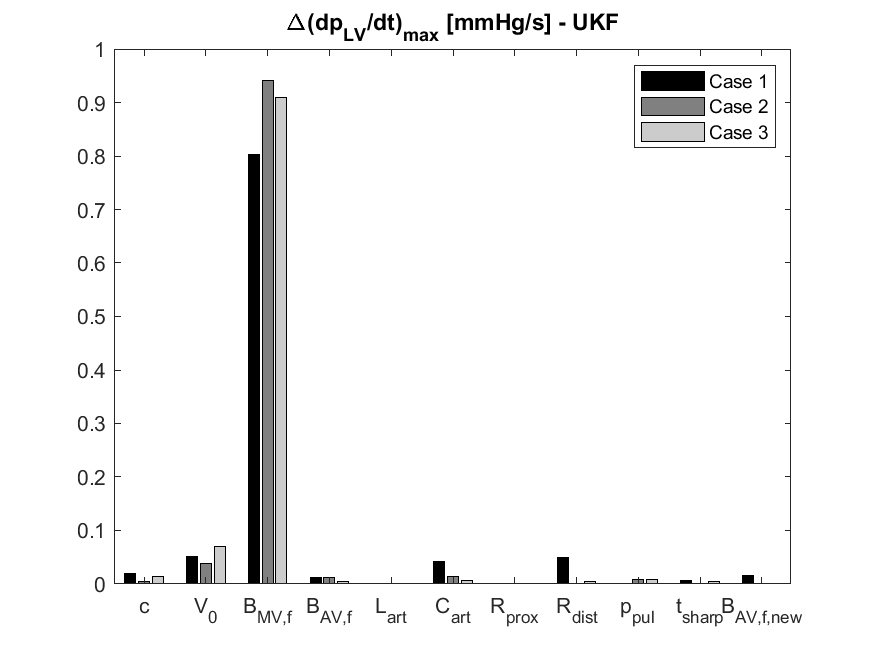

Supplement: Supplementary file 1 — Data S1. Supporting information. [file CNM-36-e3388-s001.zip › Supplementary Images/SobolTotalUKFOP6.png]

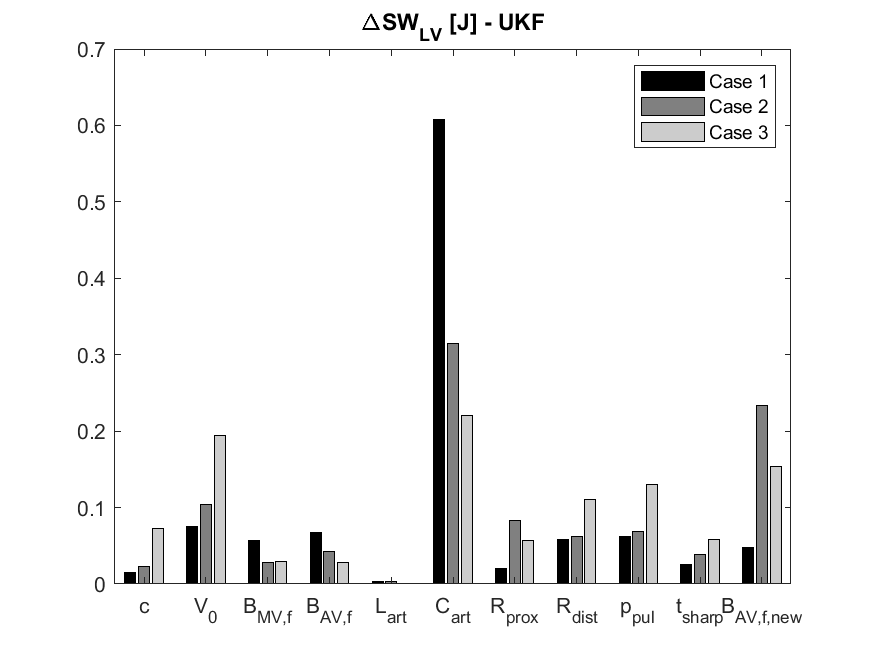

Supplement: Supplementary file 1 — Data S1. Supporting information. [file CNM-36-e3388-s001.zip › Supplementary Images/SobolTotalUKFOP7.png]

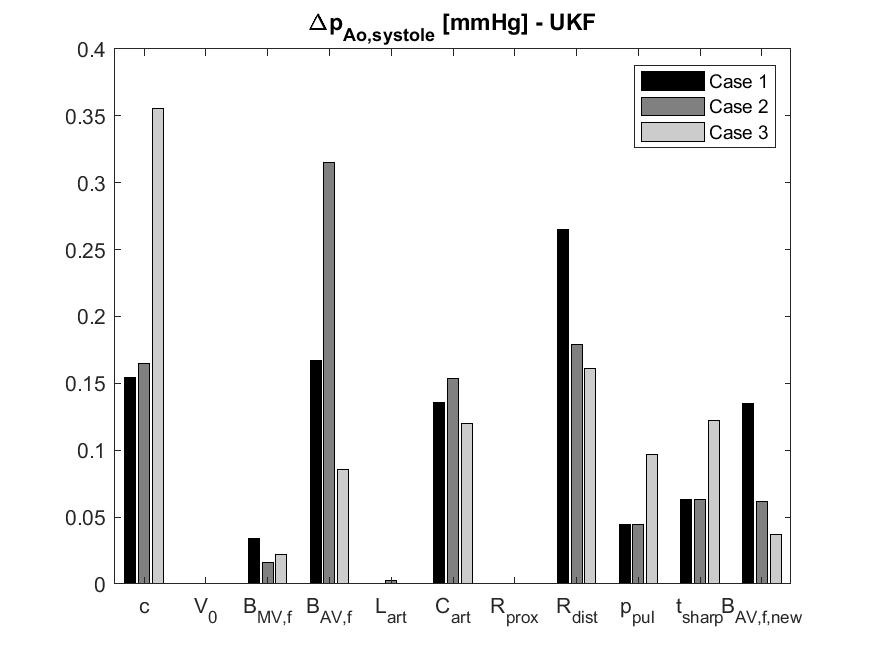

Supplement: Supplementary file 1 — Data S1. Supporting information. [file CNM-36-e3388-s001.zip › Supplementary Images/SobolTotalUKFOP8.png]

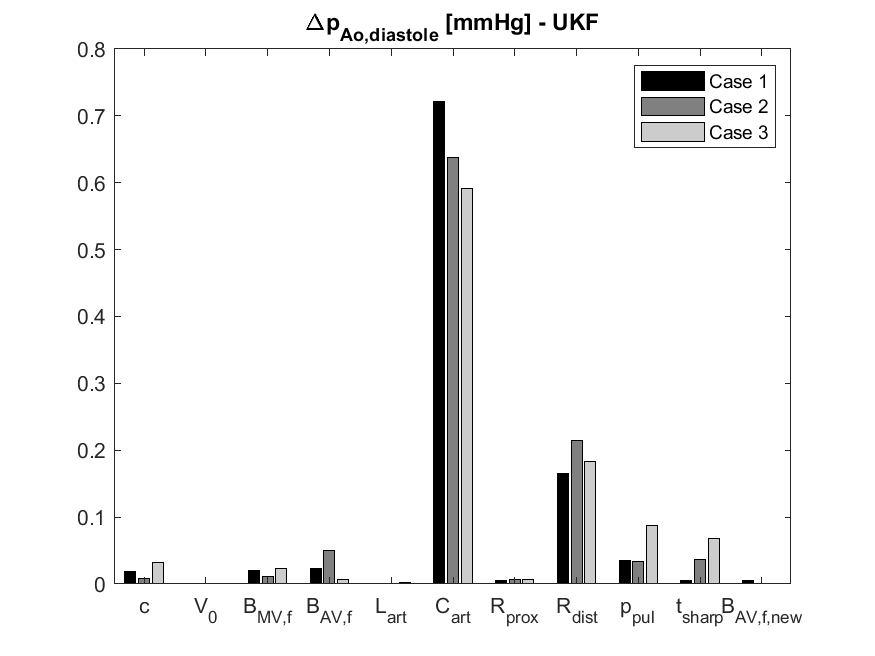

Supplement: Supplementary file 1 — Data S1. Supporting information. [file CNM-36-e3388-s001.zip › Supplementary Images/SobolTotalUKFOP9.png]

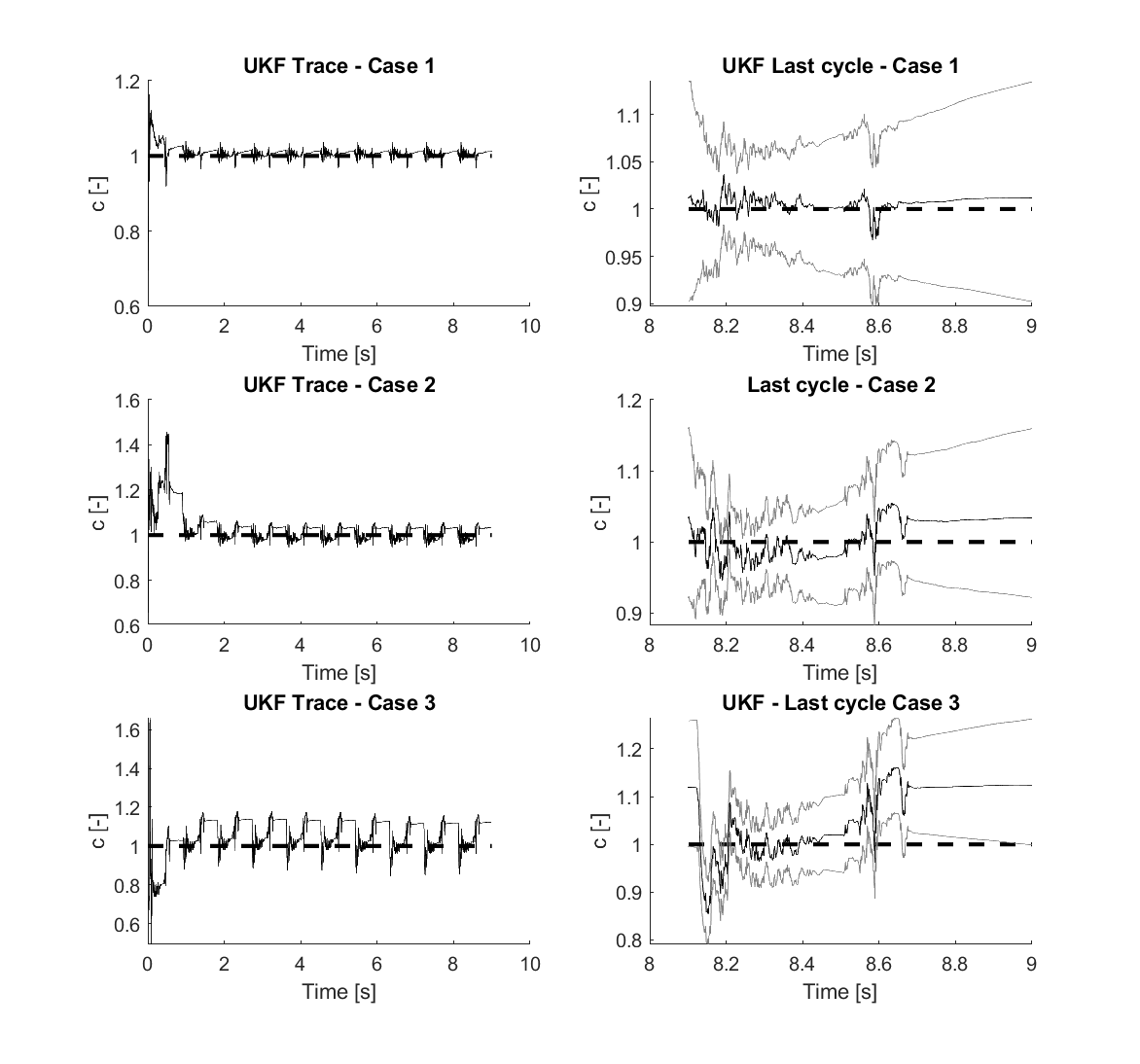

Supplement: Supplementary file 1 — Data S1. Supporting information. [file CNM-36-e3388-s001.zip › Supplementary Images/UKFParam1.png]

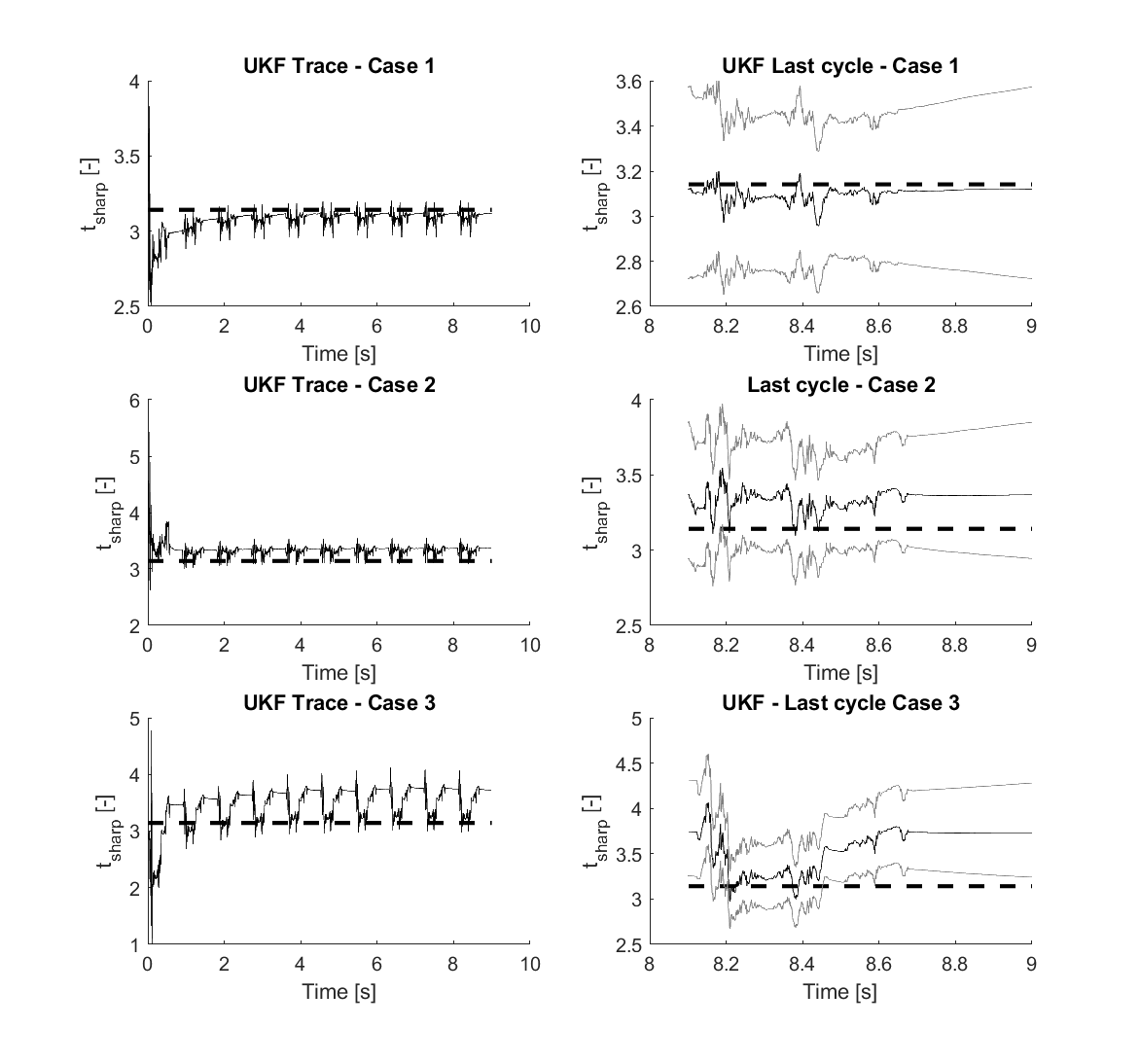

Supplement: Supplementary file 1 — Data S1. Supporting information. [file CNM-36-e3388-s001.zip › Supplementary Images/UKFParam10.png]

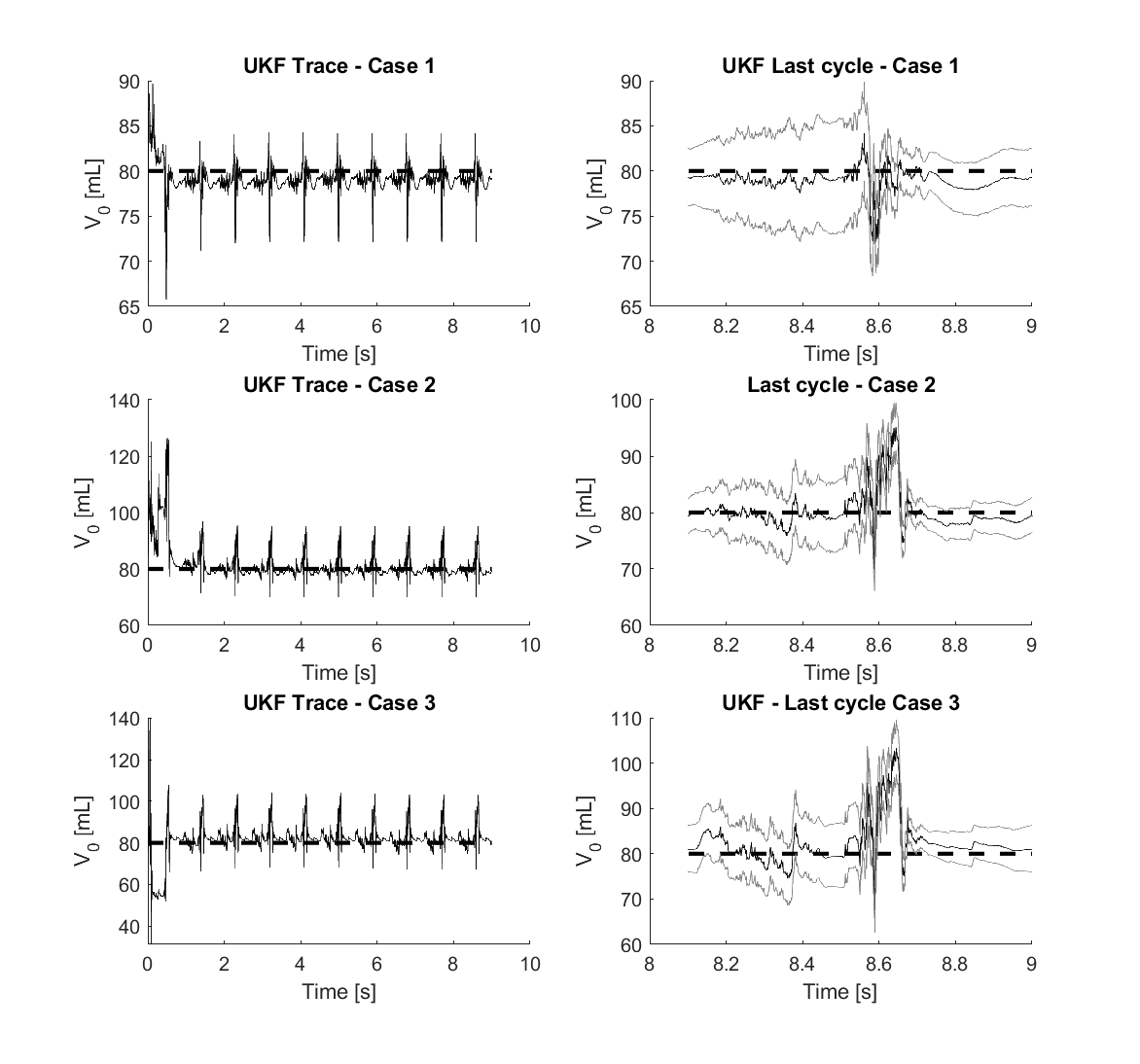

Supplement: Supplementary file 1 — Data S1. Supporting information. [file CNM-36-e3388-s001.zip › Supplementary Images/UKFParam2.png]

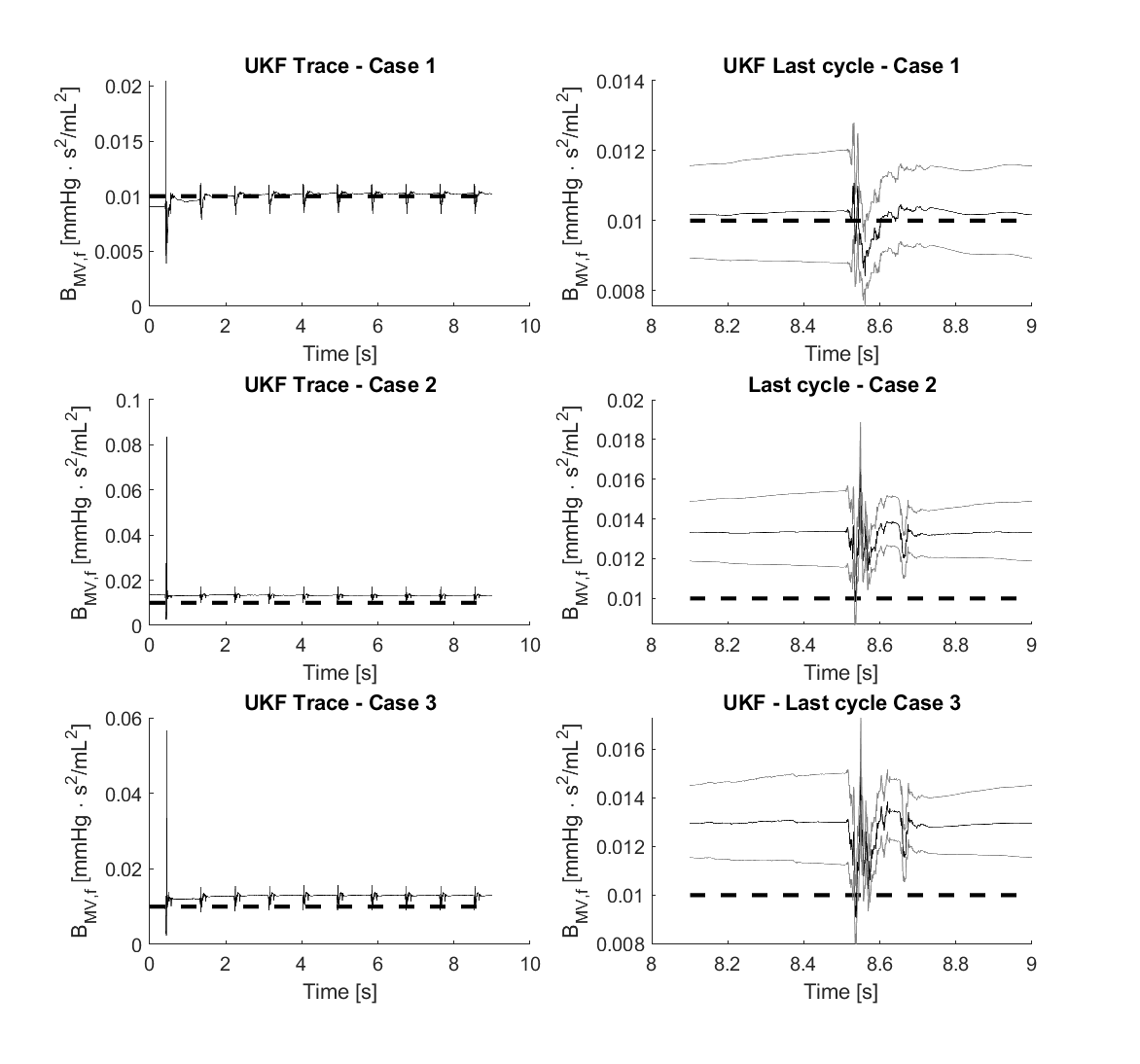

Supplement: Supplementary file 1 — Data S1. Supporting information. [file CNM-36-e3388-s001.zip › Supplementary Images/UKFParam3.png]

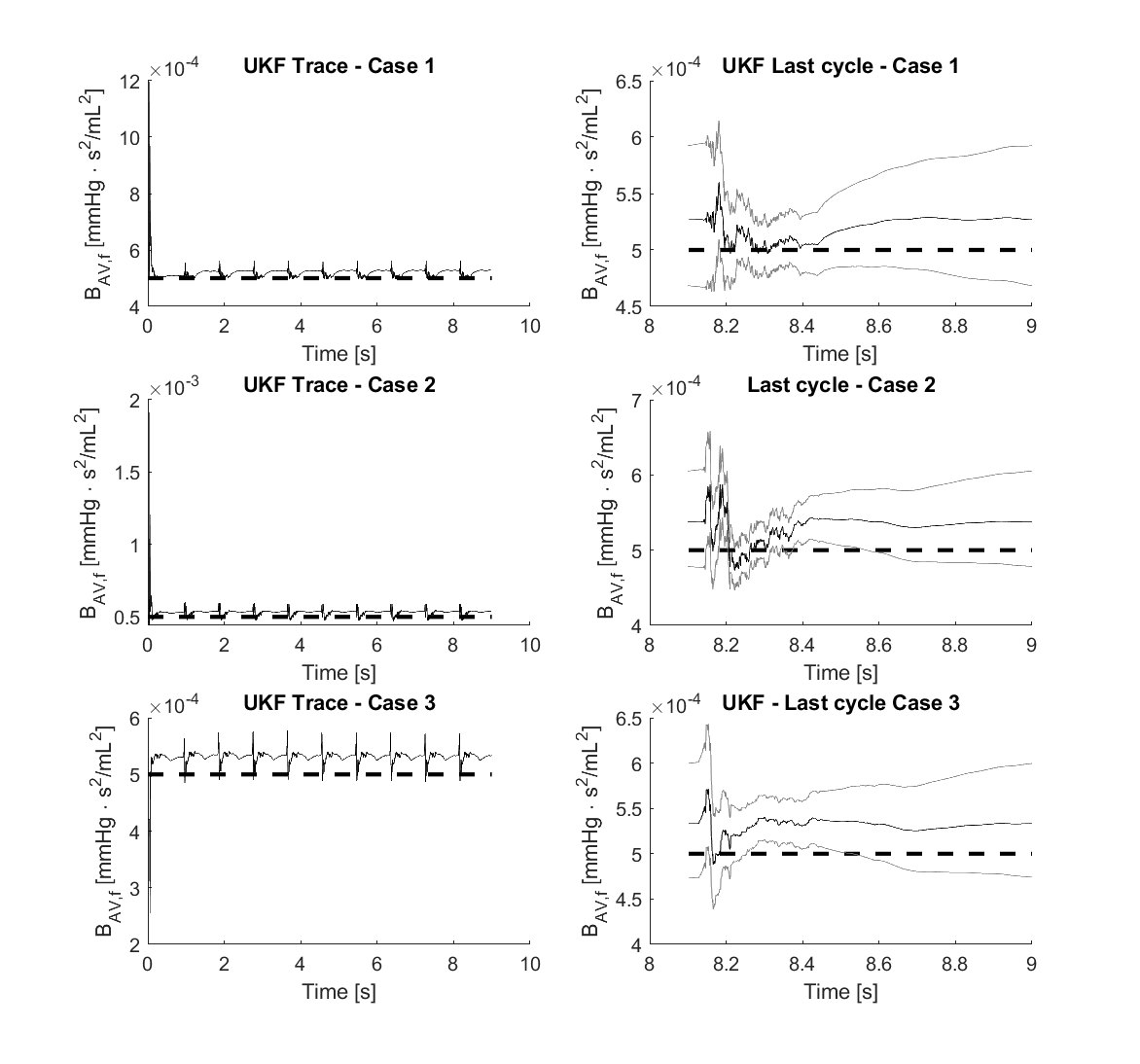

Supplement: Supplementary file 1 — Data S1. Supporting information. [file CNM-36-e3388-s001.zip › Supplementary Images/UKFParam4.png]

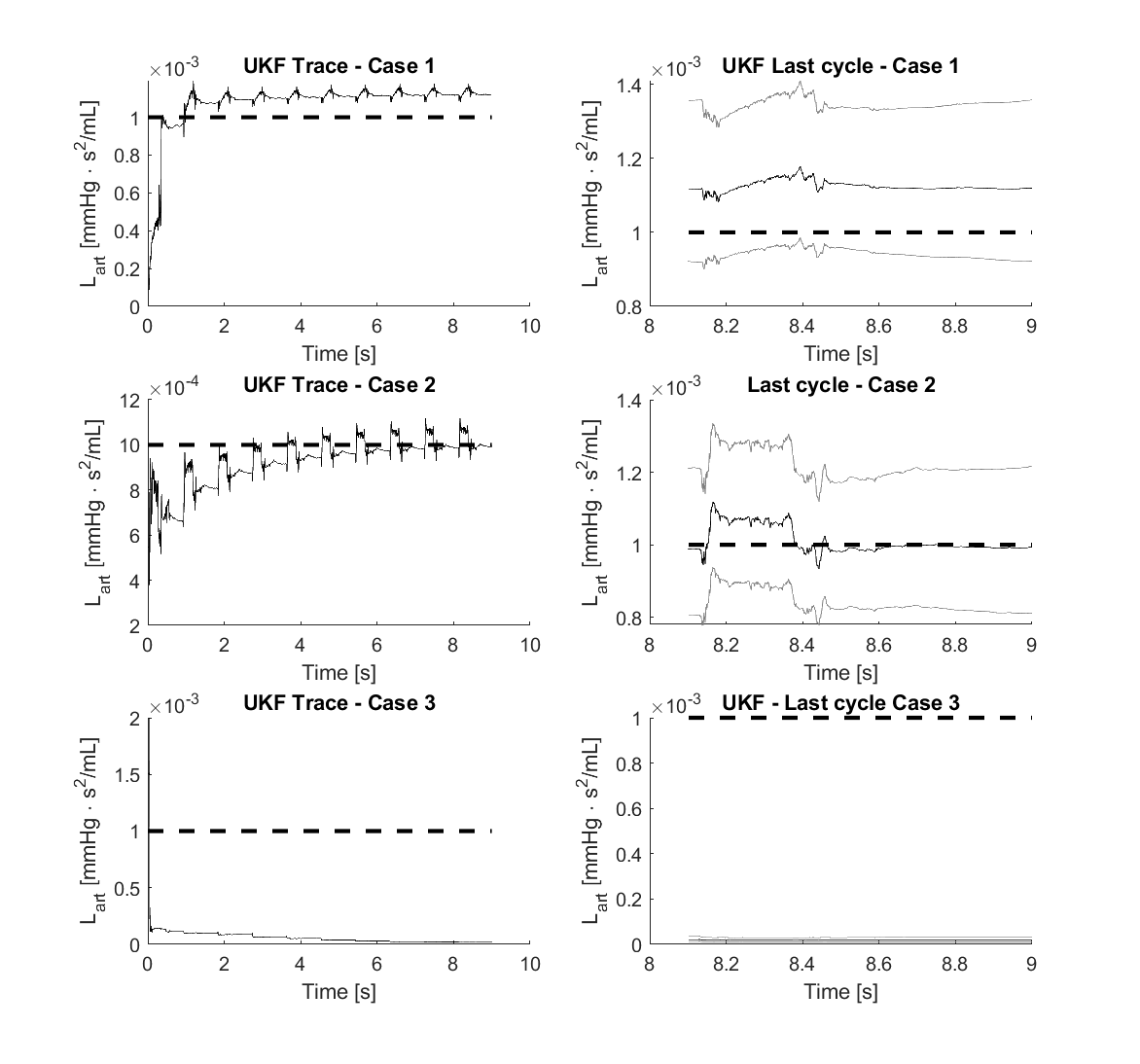

Supplement: Supplementary file 1 — Data S1. Supporting information. [file CNM-36-e3388-s001.zip › Supplementary Images/UKFParam5.png]

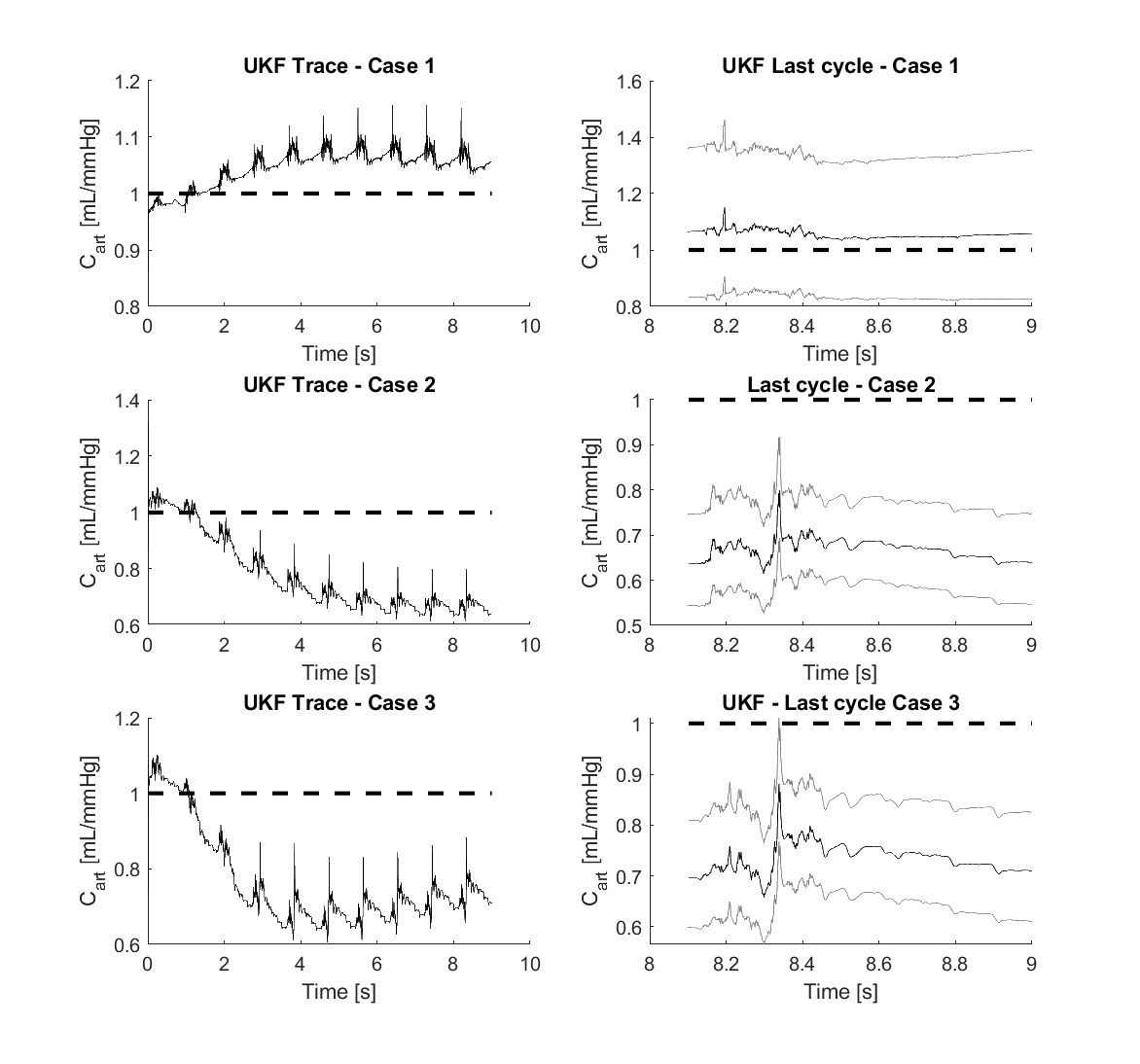

Supplement: Supplementary file 1 — Data S1. Supporting information. [file CNM-36-e3388-s001.zip › Supplementary Images/UKFParam6.png]

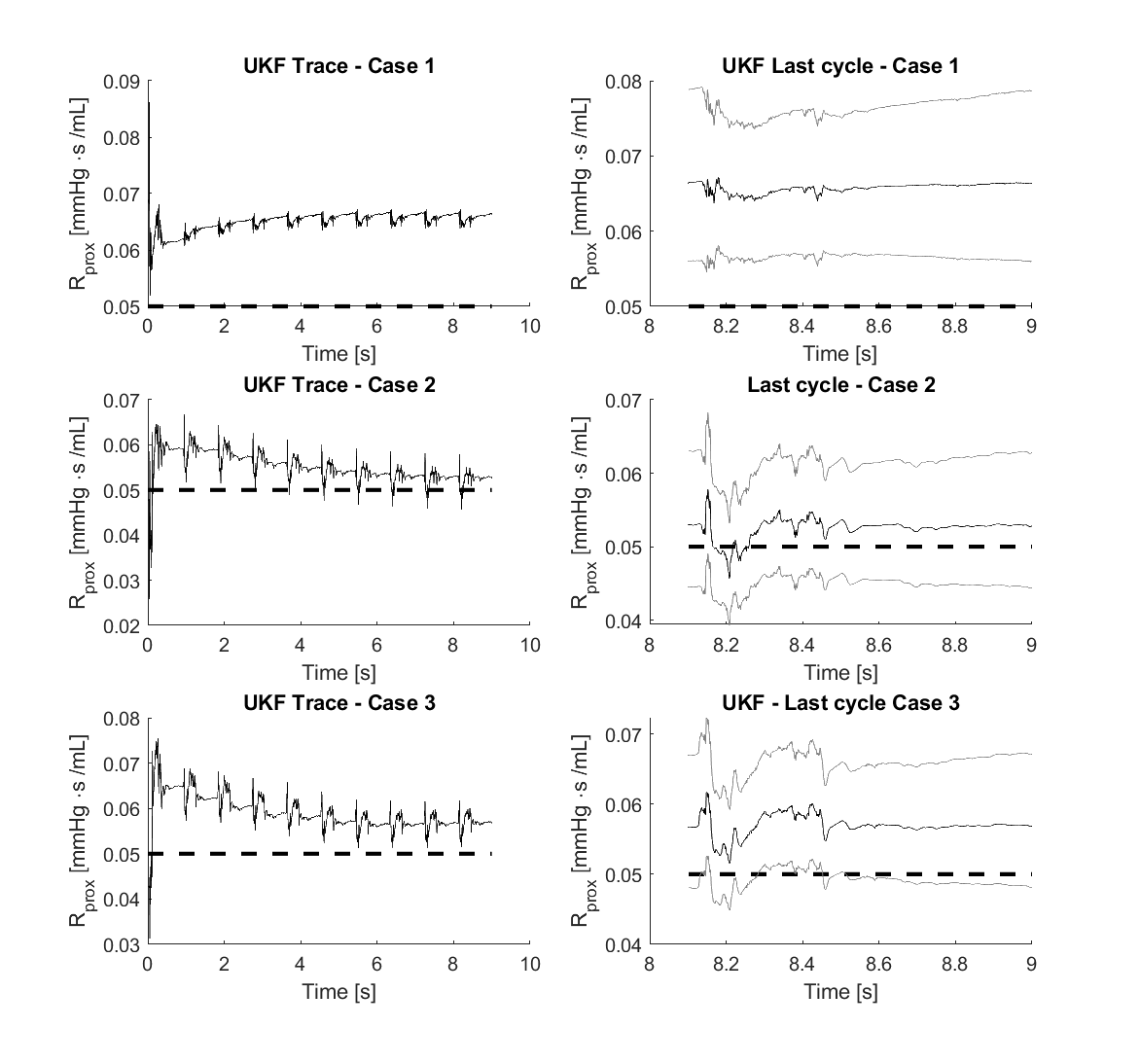

Supplement: Supplementary file 1 — Data S1. Supporting information. [file CNM-36-e3388-s001.zip › Supplementary Images/UKFParam7.png]

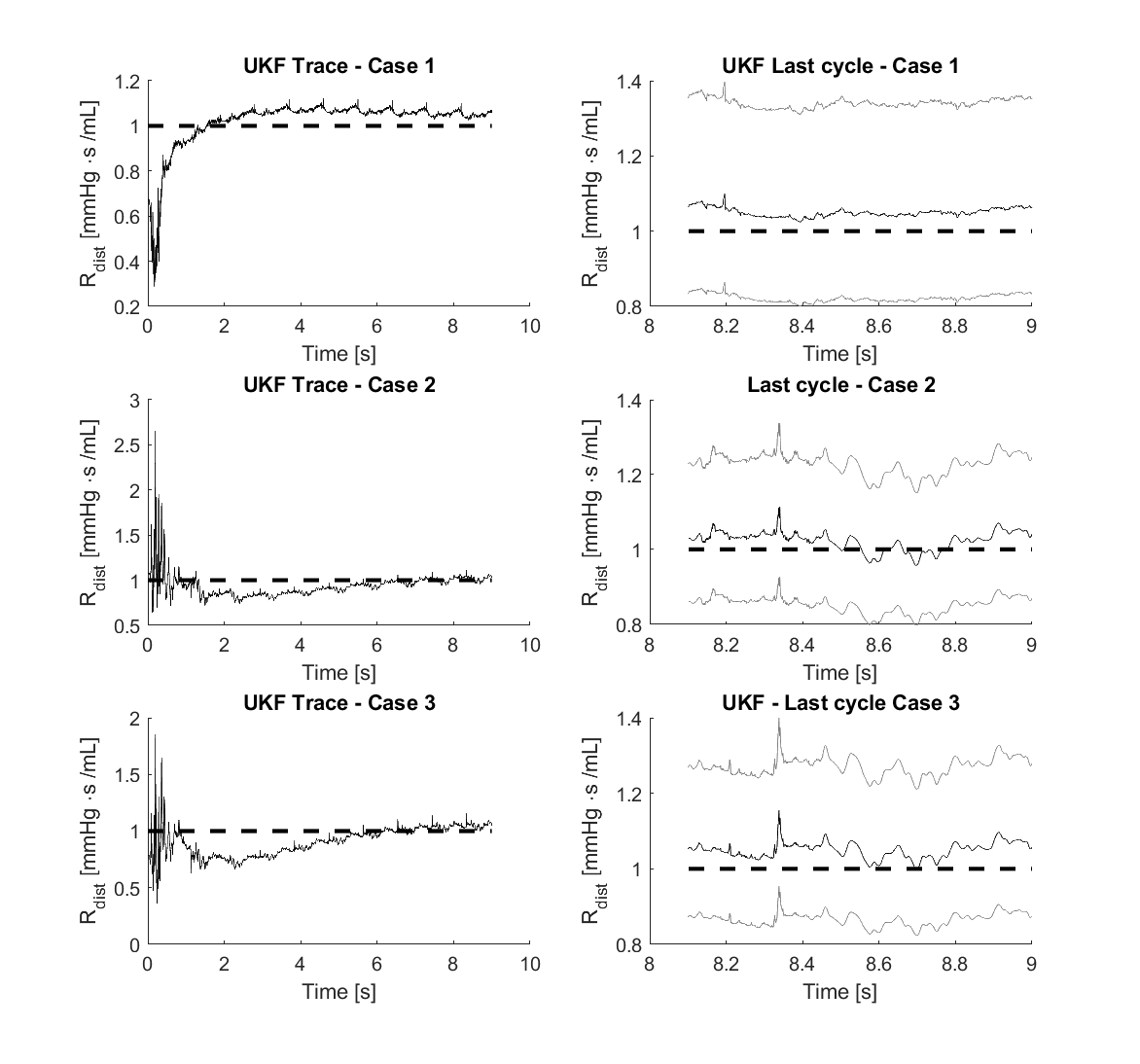

Supplement: Supplementary file 1 — Data S1. Supporting information. [file CNM-36-e3388-s001.zip › Supplementary Images/UKFParam8.png]

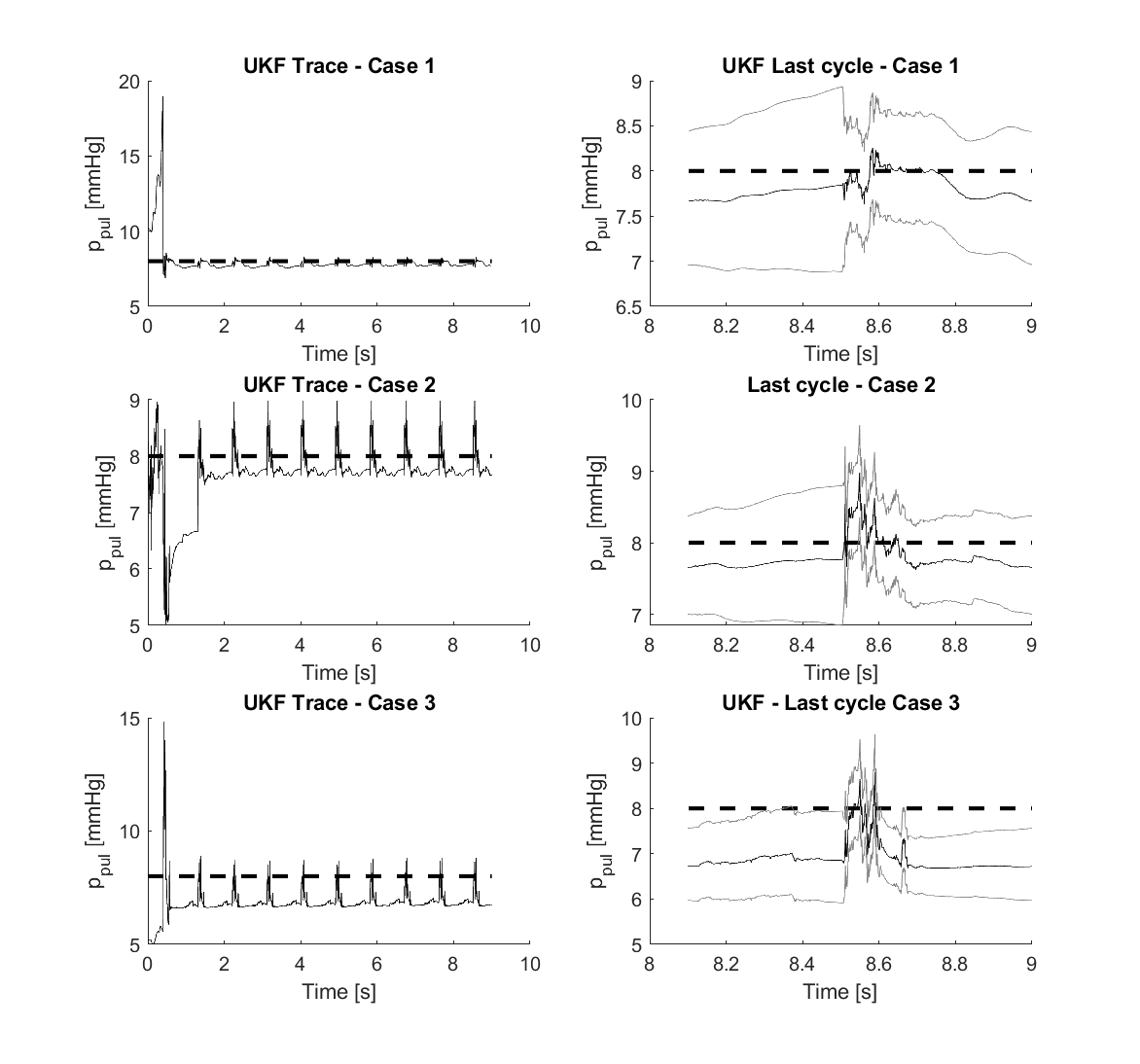

Supplement: Supplementary file 1 — Data S1. Supporting information. [file CNM-36-e3388-s001.zip › Supplementary Images/UKFParam9.png]
